# Flexible Fragment Growing Boosts Potency of Quorum‐Sensing Inhibitors against *Pseudomonas aeruginosa* Virulence

**DOI:** 10.1002/cmdc.201900621

**Published:** 2019-11-28

**Authors:** Michael Zender, Florian Witzgall, Alexander Kiefer, Benjamin Kirsch, Christine K. Maurer, Andreas M. Kany, Ningna Xu, Stefan Schmelz, Carsten Börger, Wulf Blankenfeldt, Martin Empting

**Affiliations:** ^1^ Drug Design and Optimization (DDOP) Helmholtz-Institute for Pharmaceutical Research Saarland (HIPS)-Helmholtz Centre for Infection Research (HZI) Campus E8.1 66123 Saarbrücken Germany; ^2^ Department of Pharmacy Saarland University Campus E8.1 66123 Saarbrücken Germany; ^3^ Structure and Function of Proteins (SFPR) Helmholtz Centre for Infection Research (HZI) Inhoffenstr. 7 38124 Braunschweig Germany; ^4^ Biotechnology and Bioinformatics, Institute for Biochemistry Technische Universität Braunschweig Spielmannstr. 7 38106 Braunschweig Germany; ^5^ Lehrstuhl für Biochemie Universität Bayreuth Universitätsstr. 30 95447 Bayreuth Germany; ^6^ PharmBioTec GmbH Science Park 1 66123 Saarbrücken Germany

**Keywords:** Quorum sensing, *Pseudomonas aeruginosa*, Pathoblocker, Fragment-based drug discovery, Enthalpic efficiency

## Abstract

Hit‐to‐lead optimization is a critical phase in drug discovery. Herein, we report on the fragment‐based discovery and optimization of 2‐aminopyridine derivatives as a novel lead‐like structure for the treatment of the dangerous opportunistic pathogen *Pseudomonas aeruginosa*. We pursue an innovative treatment strategy by interfering with the *Pseudomonas* quinolone signal (PQS) quorum sensing (QS) system leading to an abolishment of bacterial pathogenicity. Our compounds act on the PQS receptor (PqsR), a key transcription factor controlling the expression of various pathogenicity determinants. In this target‐driven approach, we made use of biophysical screening via surface plasmon resonance (SPR) followed by isothermal titration calorimetry (ITC)‐enabled enthalpic efficiency (EE) evaluation. Hit optimization then involved growth vector identification and exploitation. Astonishingly, the latter was successfully achieved by introducing flexible linkers rather than rigid motifs leading to a boost in activity on the target receptor and anti‐virulence potency.

## Graphical abstract

**Growth vector identification and exploitation**: Enthalpic efficiency‐driven fragment prioritization led to 2‐aminopyridines as starting points for the generation of novel pathoblockers against *P. aeruginosa*. Allowing flexibility in the hit optimization process facilitated successful fragment growing accompanied by a thousand‐fold boost in activity relative to the initial fragment.

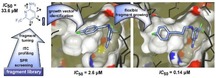

## Introduction

*Pseudomonas aeruginosa* is an opportunistic Gram‐negative pathogen. It provokes different acute and chronic infections especially in immune‐compromised and hospitalized patients.^1^ Alarmingly, the occurrence of multi‐resistant and pan‐resistant strains renders currently available antibiotics ineffective and leads to an urgent need for novel treatment options.^2^ *P. aeruginosa* employs an arsenal of virulence‐associated factors that allow this pathogen to be effective in various host organisms and environments.^3^ The release of many virulence factors is controlled and synchronized by a process called quorum sensing (QS).^4^ QS allows bacteria to collectively regulate gene expression depending on their population density. Small diffusible molecules (auto‐inducers) are secreted from the cells and once a certain threshold concentration has been achieved, transcriptional regulators are activated. This leads to a population‐wide alteration of gene expression, resulting in concerted phenotypic actions.^5^ This ability is essential during the course of acute and chronic infections^6^ as well as for lowered antibiotic susceptibility.^7^

**Figure 1:**
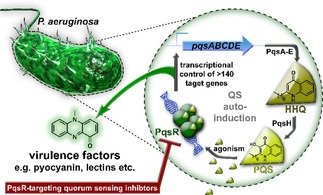
Schematic representation of the *pqs* quorum sensing system in *Pseudomonas aeruginosa* highlighting PqsR as an attractive point‐of‐intervention. Activation of PqsR by native agonists PQS (and to a lower extend HHQ) leads to autoinduction of the biosynthetic enzyme cascade PqsA−E as well as regulation of bacterial virulence like e. g. production of pyocyanin.

Respective cell‐to‐cell communication in *P. aeruginosa* is mainly based on four distinct QS circuitries. The *las* 8 and the *rhl* 9 QS systems use different *N*‐acylated homoserine lactones (AHLs) as signaling molecules, while recently discovered *iqs* relies on 2‐(2‐hydroxyphenyl)‐thiazole‐4‐carbaldehyde (Integrated Quorum Sensing Signal, IQS).^10^ AHL‐based communication is most widespread ‘language’ found in Gram‐negative bacteria.^11^ On the contrary, the forth system called *pqs* 12 employs alkylquinolones (AQs)^13^, ^14^ and occurs only in *Pseudomonas* and *Burkholderia* species (Figure 1).^15^ The signaling molecules PQS (*Pseudomonas* Quinolone Signal; 2‐heptyl‐3‐hydroxy‐4(1H)‐quinolone) and its precursor HHQ (2‐heptyl‐4(1H)‐quinolone) activate the receptor PqsR (also referred to as MvfR).^13^, ^14^ PqsR is a LysR‐type transcriptional regulator that controls a subset of genes that is responsible for the production of various virulence factors like pyocyanin, elastase, and lectins.^16^ Moreover, it drives the expression of the *pqsABCDE* operon.^13^, ^17^ This operon encodes the enzymes PqsABCDE required for the biosynthesis of HHQ,^18^ which is converted by the monooxygenase PqsH to the more potent agonist PQS. PqsR‐deficient strains showed reduced pathogenicity in several *in vivo* infection models^16^, ^19^ demonstrating its central role during the infection process. Therefore, PqsR is a potential drug target to attenuate *P. aeruginosa* virulence without affecting bacterial viability. This approach promises only a low selection pressure towards resistance development.^20^

Our group has previously obtained the first PqsR‐targeting quorum sensing inhibitor (QSI) by chemical modification of the natural ligand HHQ.^21^ These compounds were further improved regarding their efficacy in *P. aeruginosa*.^22^, ^23^ We revealed that most potent QSI of this class act as inverse agonists rather than antagonists on the target receptor.^24^ In a similar approach, QSI containing a quinazolinone scaffold was discovered and helped resolving the crystal structure of the ligand binding domain of PqsR.^25^ Furthermore, an HTS campaign led to benzamide‐benzimidazole compounds showing efficacy in different mouse models, which emphasizes the *in vivo* relevance of targeting PqsR in infectious diseases.^26^ To overcome the poor physicochemical profiles of the HHQ‐derived QSI, we initiated two fragment screenings using surface plasmon resonance (SPR) technology.^27^, ^28^ These approaches led to the hydroxamic acid **1** and the 2‐amino‐oxadiazole **2** (Table 1). First attempts to enlarge these structures have not been successful so far or led to compounds lacking activity in *P. aeruginosa* (data not shown).

**Table 1:** Thermodynamic profiling of fragment‐sized PqsR ligands guiding the selection of the optimal starting point.

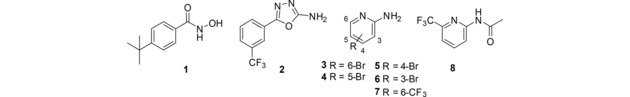						
Compound	*K*_D (ITC)_ [μM]	Δ*G* [kcal mol^−1^]	Δ*H* [kcal mol^−1^]	−*T*Δ*S* [kcal mol^−1^]	EE^[a]^ [kcal mol^−1^]	LE^[b]^ [kcal mol^−1^]
1	4.1±0.6	−7.4±0.1	−8.9±0.2	1.5±0.3	0.63	0.53
2	1.3±0.3	−8.0±0.1	−8.5±0.5	0.4±0.6	0.53	0.50
3	10.0±1.3	−6.8±0.1	−9.3±0.4	2.5±0.4	1.17	0.85
4	>50^[c]^	–	–	–	–	–
5	21.3±3.4	−6.4±0.1	−5.8±0.1	−0.6±0.2	0.72	0.80
6	>50^[c]^	–	–	–	–	–
7	3.1±0.5	−7.5±0.1	−11.5±0.9	3.7±1	1.05	0.70

ITC titrations were performed at 298 K. Data represent mean±SD from at least two independent experiments; [a] EE=−ΔH/(heavy atom count); [b] LE=−ΔG/(heavy atom count). [c] no heat release detectable at solubility maximum.

Here, we applied enthalpic efficiency^29^, ^30^ as a metric to select screening hit **3** as an alternative starting point. During initial fragment–growing efforts, we were successful in stepwise enlarging the fragment structure. Notably, we determined the crystal structure of a ligand‐receptor complex (PDB ID 6Q7 V). This enabled us to devise an elegant structure‐guided optimization strategy considering the binding pose of the natural ligand HHQ. The introduction of a flexible linker finally led to compounds with nanomolar inverse‐agonistic activities in an *E. coli* reporter gene assay and complete pyocyanin inhibition in *P. aeruginosa*.

## Results and Discussion

**Selection of an optimal starting point**. Selecting the best starting point is a pivotal decision in the early stage of a drug discovery project. In previous projects, ligand efficiency^30^ (LE) was used as major criterion for hit selection.^27^ The LE values represent the mean binding contribution per heavy atom (LE=−ΔG/N) allowing to compare hits of different sizes.^30^ Taken into account that the ligand binding site of PqsR is highly lipophilic,^25^ the engineering of well‐placed hydrogen bonds will be a difficult task. Hence, an ideal screening hit should establish most effective non‐covalent interactions. Enthalpic contribution (ΔH) is a convenient indicator for the establishment and the breaking of specific interactions during the formation of the protein‐ligand complex.^29^ In a simple model, ΔH describes the sum of bond breaks between solvent and ligand and on the other hand bond formation between ligand and protein.^29^ Hence, enthalpic efficiency (EE) was used as guideline to derive an alternative starting point. EE normalizes the enthalpic contribution for the heavy atom count (EE=−ΔH/N). Hence, this concept allows comparing ligands of different sizes.

During two previously reported fragment‐optimization approaches, compounds **1** 27 and **2** 28 (Table 1) were discovered. The thermodynamic profiles of these optimized fragments were compared to the best screening hits derived from previously reported SPR screenings.^27^, ^28^ Initially, compound **3** was ranked lower due to its lower affinity.^28^ A re‐evaluation of these three fragment scaffolds making use of the above‐mentioned ranking metrics showed that **3** possesses strikingly better EE and LE values (Table 1).

In the light of these considerations, fragment **3** was evaluated as a promising alternative starting point.

**Fragment growing**. Ideally, a fragment‐based screening campaign is supported by structure‐based drug design strategies as this improves the chances to develop highly active lead molecules tremendously.^31^ However, for **3** no crystal structure in complex with PqsR could be obtained. In order to derive a first SAR impression and to find a potential vector for growing the fragment, close commercial analogues (**4**–**8**) were investigated for their affinity to PqsR by ITC analysis (Table 1). This revealed that the position of the bromine substituent was fundamental for affinity. A bromo substituent in 3‐ and 5‐position (**4** and **6**) abolished the affinity, whereas in 4‐position (**5**) the affinity was slightly decreased, which promoted the 4‐position as potential growth vector. The exchange of the bromine for a sterically more demanding trifluoromethyl group in 6‐position (**7**) led to a threefold increase of affinity caused by a gain in the enthalpic contribution. Candidate compounds were tested in an *E. coli* lacZ reporter gene system for their ability to antagonize/inverse agonize PqsR.^32^ This heterologous system provides higher sensitivity and a clear‐cut readout due to the absence of the entire pqs system present in *P. aeruginosa*. Our previous studies indicated that the system employing an *E.coli* laboratory strain poses a less restrictive biological barrier to small molecules for reaching the intracellular target.^22^ Hence, it facilitates a straightforward evaluation and comparison of PqsR‐targeting QSI regarding their on‐target activities. Noteworthy, this assay system provides a more reliable estimation of cellular effectivity than cell‐free affinity measurements. For example, the reported affinity of the native agonist PQS towards the ligand binding domain is rather low (*K*_D_=1.2±0.3 μM).^33^ In contrast, the PQS‐mediated effect in *E.coli*‐based assays and *P. aeruginosa* occurs at drastically lower concentrations (*EC*_50(*E.coli)*_=6.3 nM,^22^ *EC*_50(*P.aeruginosa*)_=24 nM). This discrepancy might be explained by the fact that functional PqsR as a LysR‐type transcriptional regulator exists as a homotetramer capable of binding to DNA and presumably able to adopt different conformational states.^34^ This higher‐ordered architecture of the bacterial target is not resembled in cell‐free assays employing only a monomeric *N*‐terminally truncated protein.

**Figure 2:**
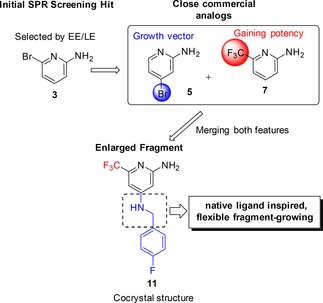
Schematic illustration of the optimization pathway starting from SPR hit **3** leading to optimized fragment **7** and identification of a growth vector (**5**) followed by fragment growing. Enlarged fragment **11** finally enabled further structure‐guided optimization through flexible fragment growing.

In contrast to the congeners (**3**–**6**; data not shown), compound **7** showed a moderate antagonistic activity (Table 2). The acetylated analog **8** was tested in order to assess the amine function as a possible handle to grow the fragment. However, the attached acetyl moiety abolished the affinity and antagonistic activity completely. Taken together, these findings led to the decision to keep scaffold **7** constant and to introduce further substituents into the 4‐position (Figure 2). At first, rigid cyclopropyl‐ethynyl (**9**) and 4‐fluorophenyl (**10**) moieties were attached as molecular probes. Both modifications abolished the antagonistic activity almost completely.

**Table 2:** Activity of compounds **7**–**22** on the target receptor PqsR.

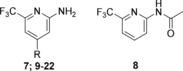			
Compd	Structure	IC_50_ [μM]^[a]^	clog*D*_7.4_^[b]^
7	H	33.6±8.2	1.7
8		n.i.	1.6
9	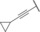	n.i.	3.1
10	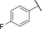	29 % @ 100 μM	
11	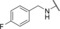	2.6±0.8	3.7
12	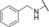	5.1±0.5	3.5
13	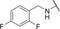	5.9±0.9	3.1
14	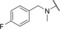	4.9±0.9	3.6
15	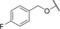	18.5±7.3	3.9
16	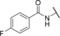	35±5 % @ 100 μM	3.6
17	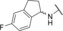	42.8±19.5	
18	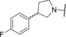	n.i.	3.3
19	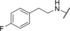	3.4±0.2	4.2
20	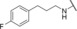	0.14±0.04	4.3
21	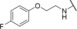	3.6±1.1	3.4
22	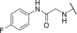	0.49±0.17	3.8

[a] Inverse agonistic/antagonistic activity was determined in the presence of 50 nM PQS; n.i.=no inhibition (activity≤15 %). IC_50_ represents the concentration of the half maximal activity; otherwise activity at a given concentration is provided. [b] Calculated using ACD/Percepta 2015.

A closer look at the thermodynamic profile of **9** disclosed a dramatic loss in the enthalpic contribution (SI Table S1: **7**: ΔH=−11.5 kcal/mol vs. **9**: ΔH=−3.7 kcal/mol) that is partially compensated by a gain in entropy. These findings suggest that the formation of specific interactions were hindered due to the attached moiety.

**Figure 3:**
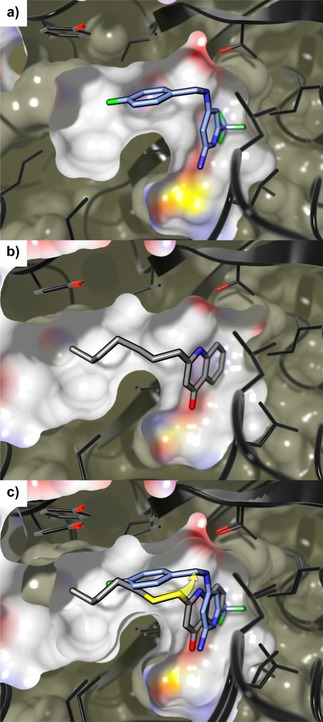
a) Crystal structure of **11** (light blue carbon) in complex with PqsR_91‐319_ (ribbon: black carbon; surface: white carbon; PDB ID 6Q7V). b) Crystal structure of HHQ (grey carbon) in complex with PqsR_91‐319_ (ribbon: black carbon; surface: white carbon; PDB ID 6Q7U). c) Overlay of a) and b). The yellow arrow indicates the applied rational for linker modification toward **20**. Fluorine: green, nitrogen: blue, oxygen: red, sulfur: yellow. Hydrogen omitted for clarity.

Hence, we introduced a more flexible linker into the 4‐position with the aim to regain specific interactions of the amino‐pyridine headgroup. For this purpose, several compounds enlarged with different benzylamine moieties (**11**–**13**) were synthesized. Indeed, these compounds (**11**–**12**) displayed up to 12‐fold increase in the on‐target activities. Overall, the 4‐fluoro substituted compound **11** evolved as the most potent QSI of this series so far. Notably, also the affinity measured by ITC was improved up to 5‐fold. (SI, Table S1). Taken together, the following general concept was derived from these previous findings (Figure 2): the amino‐pyridine headgroup should be connected via a flexible linker part to another aromatic moiety. We were able to solve a co‐crystal structure of **11** in complex with the ligand binding domain of PqsR (Figure 3a), clearly showing that the linker establishes an angled connection between the 2‐amino‐pyridine headgroup, which occupies the space of the quinolone core of the natural ligands while the 4‐fluorophenyl ring which points into the alkyl‐chain pocket. Both aromatic systems of **11** are flanked by the alkyl side chains of isoleucine residues enabling CH‐π interactions. The 2‐amino moiety can form an H‐bond interaction with the backbone carbonyl of Leu207 and the NH of the benzylamine linker interacted with the hydroxyl moiety of Thr265. However, a comparison of the thermodynamic signatures of **11** binding to PqsR_wt_ and PqsR_T265A_, respectively (SI, Figure S3) displayed no significant difference in the enthalpic contribution, which argues against a beneficial H‐bond interaction. Notably, we detected an additional putative H‐bond between the pyridine nitrogen lone pair and a water molecule that had been observed in ligand‐receptor‐complexes of the congener **22** (described below) and PDB entry 4JVC.^25^

After obtaining this structural knowledge it became clear, why linear compounds **9** and **10** showed low or even no activity although they were enlarged along the correct growth vector. We concluded that a certain flexibility in the linker part is essential for the compound to adopt an angled binding conformation. Hence, in the further proceeding, modifications of this region were prioritized (Figure 2) in order to optimize compound **11**.

Following this strategy, we evaluated the role of the NH within the linker through synthesis of *N*‐methylated derivative **14** and benzyl alcohol **15**. Compound **14** showed a slightly decreased on‐target activity corroborating the notion that the H‐bond observed in the X‐ray structure of **11** does not translate into a significant gain in binding affinity. Interestingly, the exchange of NH to O (compare **11** and **15**) led to a more significant drop in potency.

Usually, unfavorable entropic penalties resulting from ligand flexibility are addressed by rigidification of compounds through reduction of rotatable bonds.^35^ Hence, we made several efforts to introduce less flexible and cyclic structures into the linker region (**16**–**18**). However, none of these modifications led to improved potency. For example, the rigidified amide linker (**16**) is not able to adapt the angled conformation of **11**. As a consequence, the antagonistic activity was almost abolished. The same trend was observed for cyclized derivatives **17** and **18**. Especially in the case of the indane derivative **17**, this loss of activity was surprising. Based on the crystal structure of **11** we assumed that neither the angled conformation nor the observed interactions would be affected by introduction of this 5‐membered ring (SI, Figure S2). These findings underline that the optimal geometric arrangement of the pyrimidine headgroup and the second aromatic system is difficult to be mimicked by rigid ligand structures. In this regard, we used the crystal structure in complex with the native ligand HHQ (Figure 3b).

**Figure 4:**
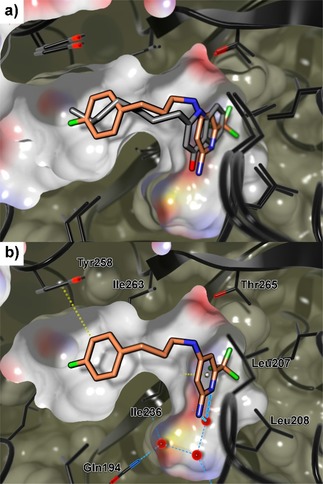
a) Overlay of crystal structures of 20 (orange carbon) and HHQ (grey carbon) in complex with PqsR91‐319 (ribbon: black carbon; surface: white carbon; PDB ID 6Q7 W). b) Minimal energy structure of 20 derived from a constrained molecular dynamics simulation based on solved X‐ray structure. The main interactions of 20 with PqsR observed within 300 ps of simulation are shown (hydrogen bonds: light blue, CH‐π interactions: yellow). Fluorine: green, nitrogen: blue, oxygen: red, sulfur: yellow. Hydrogen omitted for clarity.

A superimposition with HHQ and **11** (Figure 3c) raised the idea to introduce a prolonged, even more flexible linker to mimic the alkyl sidechain of these compounds. Therefore, compounds **19**–**22** were synthesized. The activity of **19** against PqsR was unchanged compared to **11**. Interestingly, introduction of four‐atom linkers resulted in remarkable potencies in the heterologous reporter gene assay (**20**–**22**). The *N*‐propyl amine linker (**20**) showed a 20‐fold boost in potency (Table 2; **11** IC_50_=2.6±0.8 μM vs. **20** IC_50_=0.14±0.04 μM). A co‐crystal structure of **20** in complex with PqsR_91‐319_ showed that the extended linker pointed deeper into the pocket occupied by the alkyl chain of the natural ligand, enabling additional CH‐π interactions with Tyr258 while the interactions of the aminopyridine head group were retained (Figure 4).

Lastly, we exchanged the alkyl chain (**20**) by an ethanolamine (**21**) and glycine (**22**) linker moiety to lower the clog*D* values. Compound **22** showed only a slightly reduced antagonistic activity, whereas **21** showed a 20‐fold drop. Overall, **20** emerged as the frontrunner compound from this fragment‐growing approach, showing a superior combination of nanomolar inverse agonistic/antagonistic activity, enthalpy‐driven binding (EE=0.50 kcal/mol, SI, Table S1) and reasonable physicochemical properties (clog*D*=3.8). Remarkably, in contrast to the usual drug optimization process relying on reducing the amount of rotatable bonds, we gained potency through allowing ligand flexibility.

**Effects in** ***P. aeruginosa***. Pyocyanin is one of the most prominent and characteristic virulence factors released by *P. aeruginosa* during acute and chronic infections.^36^ The redox‐active blue pigment has cytotoxic and immunomodulating properties.^36^ Moreover, it has been clearly demonstrated that pyocyanin promotes the development of a pulmonary pathophysiology in mice similar to the lung of infected cystic fibrosis patients.^37^ Hence, it was of major interest to demonstrate that 2‐amino‐pyridines were able to translate the PqsR‐directed activity measured in *E.coli* into antivirulence activity in *P. aeruginosa*. Compounds **7**, **11** and **20**–**22** were evaluated for the inhibition of pyocyanin in the highly virulent clinical isolate PA14 (Table 3). In this regard, it is important to point out that *P. aeruginosa* shows low intrinsic outer‐membrane permeability for xenobiotics and applies a multitude of different efflux pumps.^36^

**Table 3:** Effects on pyocyanin in *P. aeruginosa*.

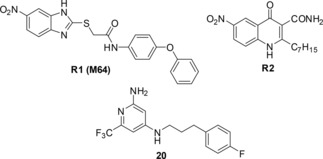			
	Pyocyanin^[a]^ IC_50_ [μM]	PEI^[b]^	MW
R1	0.12	0.0165	420.4
R2	2.0	0.0172	331.4
7	43 % @ 250 μM	–	162.1
11	102	0.0140	285.2
20	5.9	0.0168	313.3
21	58 % @ 100 μM	–	315.3
22	37 % @ 100 μM	–	328.3

[a] Photometric quantification of pyocyanin from PA14 cultures; given values represent the mean of at least two independent experiments; SD<25 %. [b] PEI Pyocyanin Efficiency Index PEI=pIC_50_/MW.

Astonishingly, fragment **7** already showed an inhibitory significant effect on pyocyanin, albeit at high concentration.

Compound **11** showed low micromolar activity in the *E.coli* assay (IC_50_=2.6±0.8 μM) and inhibited pyocyanin with an IC_50_ of 105±13 μM. Optimized hit **20** displayed the highest cellular efficacy of this new class of QSI (IC_50_=5.9±0.7 μM). The literature‐known compounds **R1**–**R2** were included as references in Table 3. The PqsR antagonist **R1** originated from an HTS screening.^26^ **R2** was discovered in‐house by modification of HHQ.^22^ We established a metric called PEI (pyocyanin efficiency index; Table 3), which normalized the effect on pyocyanin for the molecular weight of the inhibitors to enable a better comparison. The optimized and lead‐like hit **20** displayed efficiency in the range of the benchmark compounds **R1** and **R2**. This clearly emphasized the potential of newly discovered QSI **20**. In contrast, slightly less active compounds **21** and **22** were not able to inhibit pyocyanin to the same extent. This might be due to efflux or permeation problems. The phenomenon that even small changes within a molecule can have drastic effects on the activity in *P. aeruginosa* was also observed in other projects and emphasizes the need for further insights on the requirement for intracellular activity, especially in the case of Gram‐negative pathogens.^22^, ^38^

The activation of PqsR drives the expression of the HHQ biosynthesis operon *pqsA‐E*, leading to enhanced alkyl quinolone (AQ) levels and finally to an auto‐inductive loop. *P. aeruginosa* produces more than 50 different AQ congeners.^39^ Among others HHQ and HQNO are most prevalent.^39^ HQNO showed Gram‐positive antibacterial activity that promotes a growth advantage for *P aeruginosa* in mixed microbial communities.^40^ Subsequently, the most interesting compounds were also evaluated for their effect on HHQ and HQNO levels in a PA14*pqsH* strain. QSI **11** was able to affect HHQ (48±4 % inhibition at 200 μM) and HQNO (36±3 % inhibition at 200 μM). The enlarged compound **20** showed an improved efficacy (56±4 % HHQ inhibition at 100 μM and 48±1 HQNO inhibition at 100 μM). These results were in line with already reported data showing that the expression of virulence factors and the levels of AQs are differently sensitive to the antagonism/inverse agonism of PqsR.^22^, ^26^

## Conclusion

The rapid development and spreading of resistances against conventional antibiotics has raised the awareness of a broader public in recent years. This ability of pathogens demonstrates the power of evolutionary adaption processes.^41^ The development of PqsR‐targeting QSI pursues the strategy to limit the virulence of *P. aeruginosa* without affecting bacterial fitness. Hence, these antivirulence compounds might be more robust to adaptive evolution because they avoid exerting high selective pressure.^42^

Herein, we report on the discovery and optimization of 2‐amino‐pyridines as promising PqsR‐targeting QSI. Starting from an SPR fragment screening^28^ ligand **3** was selected based on its unique EE, although it did not show significant antagonistic activity. Successfully growing and improving a fragment‐screening hit without structural information is a challenging approach. Therefore, close commercial analogs were investigated, leading to compound **7**, which showed improved affinity and comparable EE. Furthermore, this intermediate compound possessed moderate antagonistic activity. By the introduction of a flexible benzyl‐amine linker into the 4‐position (**11**) affinity and on‐target activity were improved drastically. The solution of the crystal structure of **11** in complex with its bacterial target PqsR facilitated further structure‐guided optimization. Notably, the ligand assumed an angled conformation spanning over the quinolone‐ and alkyl chain‐pocket. Inspired by the alkyl chain of the natural ligand HHQ, we extended the linker moiety, which led to the top candidate **20** showing inverse agonistic/antagonistic activity in the nanomolar range. The rather unusual approach of allowing more flexibility instead of rigidifying the ligand might be also applicable to a broader scope of druggable proteins. Especially in cases where the biological function of the drug target depends on different conformational states (e. g. inducible transcriptional regulators), this flexible fragment‐growing strategy might be a valuable tool to rapidly generate lead‐like molecules. Usually, a selected fragment‐sized hit already provides efficient directed interactions with the target. Allowing compound flexibility during the enlargement process could help to retain the beneficial binding geometry of the fragment core while granting access to additional sites of the binding pocket resulting in enhanced potency.

The discovered PqsR‐targeting QSI is a remarkably small compound and efficiently reduces pyocyanin in *P. aeruginosa* without affecting bacterial viability. Compound **20** represents an excellent starting point for further lead generation and optimization studies.

## Supporting information

As a service to our authors and readers, this journal provides supporting information supplied by the authors. Such materials are peer reviewed and may be re‐organized for online delivery, but are not copy‐edited or typeset. Technical support issues arising from supporting information (other than missing files) should be addressed to the authors.

Supplementary

## References

[1] G. P. Bodey , R. Bolivar , V. Fainstein , L. Jadeja , Rev. Infect. Dis. 1983, 5, 279. 6405475 10.1093/clinids/5.2.279

[2] V. Aloush , S. Navon-Venezia , Y. Seigman-Igra , S. Cabili , Y. Carmeli , Antimicrob. Agents Chemother. 2006, 50, 43. 16377665 10.1128/AAC.50.1.43-48.2006 PMC1346794

[3] L. G. Rahme , F. M. Ausubel , H. Cao , E. Drenkard , B. C. Goumnerov , G. W. Lau , S. Mahajan-Miklos , J. Plotnikova , M. W. Tan , J. Tsongalis , Proc. Natl. Acad. Sci. USA 2000, 97, 8815. 10922040 10.1073/pnas.97.16.8815 PMC34017

[4] C. van Delden , B. H. Iglewski , Emerging Infect. Dis. 1998, 4, 551. 9866731 10.3201/eid0404.980405 PMC2640238

[5] N. A. Whitehead , A. M. Barnard , H. Slater , N. J. Simpson , G. P. Salmond , FEMS Microbiol. Rev. 2001, 25, 365. 11524130 10.1111/j.1574-6976.2001.tb00583.x

[6a] M. Kesarwani , R. Hazan , J. He , Y.-A. Que , Y. Que , Y. Apidianakis , B. Lesic , G. Xiao , V. Dekimpe , S. Milot , PLoS Pathog. 2011, 7, e1002192; 21829370 10.1371/journal.ppat.1002192 PMC3150319

[6b] C. T. Parker , V. Sperandio , Cell. Microbiol. 2009, 11, 363. 19068097 10.1111/j.1462-5822.2008.01272.x PMC2786497

[7] T. Bjarnsholt , P. Ø. Jensen , M. Burmølle , M. Hentzer , J. A. J. Haagensen , H. P. Hougen , H. Calum , K. G. Madsen , C. Moser , S. Molin , Microbiology (Reading, England) 2005, 151, 373. 10.1099/mic.0.27463-0 15699188

[8] L. Passador , J. M. Cook , M. J. Gambello , L. Rust , B. H. Iglewski , Science (New York, N. Y.) 1993, 260, 1127. 10.1126/science.8493556 8493556

[9] U. A. Ochsner , A. K. Koch , A. Fiechter , J. Reiser , J. Bacteriol. 1994, 176, 2044. 8144472 10.1128/jb.176.7.2044-2054.1994 PMC205310

[10a] J. Lee , J. Wu , Y. Deng , J. Wang , C. Wang , J. Wang , C. Chang , Y. Dong , P. Williams , L.-H. Zhang , Nat. Chem. Biol. 2013, 9, 339; 23542643 10.1038/nchembio.1225

[10b] U. A. Ochsner , J. Reiser , Proc. Natl. Acad. Sci. USA 1995, 92, 6424; 7604006 10.1073/pnas.92.14.6424 PMC41530

[10c] J. P. Pearson , K. M. Gray , L. Passador , K. D. Tucker , A. Eberhard , B. H. Iglewski , E. P. Greenberg , Proc. Natl. Acad. Sci. USA 1994, 91, 197. 8278364 10.1073/pnas.91.1.197 PMC42913

[11] S. Schauder , B. L. Bassler , Genes Dev. 2001, 15, 1468. 11410527 10.1101/gad.899601

[12] E. C. Pesci , J. B. Milbank , J. P. Pearson , S. McKnight , A. S. Kende , E. P. Greenberg , B. H. Iglewski , Proc. Natl. Acad. Sci. USA 1999, 96, 11229. 10500159 10.1073/pnas.96.20.11229 PMC18016

[13] D. S. Wade , M. W. Calfee , E. R. Rocha , E. A. Ling , E. Engstrom , J. P. Coleman , E. C. Pesci , J. Bacteriol. 2005, 187, 4372. 15968046 10.1128/JB.187.13.4372-4380.2005 PMC1151766

[14] G. Xiao , E. Déziel , J. He , F. Lépine , B. Lesic , M.-H. Castonguay , S. Milot , A. P. Tampakaki , S. E. Stachel , L. G. Rahme , Mol. Microbiol. 2006, 62, 1689. 17083468 10.1111/j.1365-2958.2006.05462.x

[15] S. P. Diggle , P. Lumjiaktase , F. Dipilato , K. Winzer , M. Kunakorn , D. A. Barrett , S. R. Chhabra , M. Cámara , P. Williams , Chem. Biol. 2006, 13, 701. 16873018 10.1016/j.chembiol.2006.05.006

[16] E. Déziel , S. Gopalan , A. P. Tampakaki , F. Lépine , K. E. Padfield , M. Saucier , G. Xiao , L. G. Rahme , Mol. Microbiol. 2005, 55, 998. 15686549 10.1111/j.1365-2958.2004.04448.x

[17] G. Xiao , J. He , L. G. Rahme , Microbiology (Reading, England) 2006, 152, 1679. 10.1099/mic.0.28605-0 16735731

[18a] E. Déziel , F. Lépine , S. Milot , J. He , M. N. Mindrinos , R. G. Tompkins , L. G. Rahme , Proc. Natl. Acad. Sci. USA 2004, 101, 1339; 14739337 10.1073/pnas.0307694100 PMC337054

[18b] C. E. Dulcey , V. Dekimpe , D.-A. Fauvelle , S. Milot , M.-C. Groleau , N. Doucet , L. G. Rahme , F. Lépine , E. Déziel , Chem. Biol. 2013, 20, 1481. 24239007 10.1016/j.chembiol.2013.09.021 PMC3877684

[19] H. Cao , G. Krishnan , B. Goumnerov , J. Tsongalis , R. Tompkins , L. G. Rahme , Proc. Natl. Acad. Sci. USA 2001, 98, 14613. 11724939 10.1073/pnas.251465298 PMC64730

[20] J. P. Gerdt , H. E. Blackwell , ACS Chem. Biol. 2014, 9, 2291. 25105594 10.1021/cb5004288 PMC4201345

[21] C. Lu , B. Kirsch , C. Zimmer , J. C. de Jong , C. Henn , C. K. Maurer , M. Müsken , S. Häussler , A. Steinbach , R. W. Hartmann , Chem. Biol. 2012, 19, 381. 22444593 10.1016/j.chembiol.2012.01.015

[22] C. Lu , C. K. Maurer , B. Kirsch , A. Steinbach , R. W. Hartmann , Angew. Chem. Int. Ed. 2014, 53, 1109. 10.1002/anie.201307547 24338917

[23] C. Lu , B. Kirsch , C. K. Maurer , J. C. de Jong , A. Braunshausen , A. Steinbach , R. W. Hartmann , Eur. J. Med. Chem. 2014, 79, 173. 24735643 10.1016/j.ejmech.2014.04.016

[24] A. A. M. Kamal , L. Petrera , J. Eberhard , R. W. Hartmann , Org. Biomol. Chem. 2017, 15, 4620. 28513746 10.1039/c7ob00263g

[25] A. Ilangovan , M. Fletcher , G. Rampioni , C. Pustelny , K. Rumbaugh , S. Heeb , M. Cámara , A. Truman , S. R. Chhabra , J. Emsley , PLoS Pathog. 2013, 9, e1003508. 23935486 10.1371/journal.ppat.1003508 PMC3723537

[26] M. Starkey , F. Lepine , D. Maura , A. Bandyopadhaya , B. Lesic , J. He , T. Kitao , V. Righi , S. Milot , A. Tzika , PLoS Pathog. 2014, 10, e1004321. 25144274 10.1371/journal.ppat.1004321 PMC4140854

[27] T. Klein , C. Henn , J. C. de Jong , C. Zimmer , B. Kirsch , C. K. Maurer , D. Pistorius , R. Müller , A. Steinbach , R. W. Hartmann , ACS Chem. Biol. 2012, 7, 1496. 22765028 10.1021/cb300208g

[28] M. Zender , T. Klein , C. Henn , B. Kirsch , C. K. Maurer , D. Kail , C. Ritter , O. Dolezal , A. Steinbach , R. W. Hartmann , J. Med. Chem. 2013, 56, 6761. 23919758 10.1021/jm400830r

[29] J. E. Ladbury , G. Klebe , E. Freire , Nature reviews. Drug discovery 2010, 9, 23. 19960014 10.1038/nrd3054

[30] A. L. Hopkins , C. R. Groom , A. Alex , Drug Discovery Today 2004, 9, 430. 15109945 10.1016/S1359-6446(04)03069-7

[31] P. J. Hajduk , J. Greer , Nature reviews. Drug discovery 2007, 6, 211. 17290284 10.1038/nrd2220

[32] C. Cugini , M. W. Calfee , J. M. Farrow , D. K. Morales , E. C. Pesci , D. A. Hogan , Mol. Microbiol. 2007, 65, 896. 17640272 10.1111/j.1365-2958.2007.05840.x

[33] M. Welch , J. T. Hodgkinson , J. Gross , D. R. Spring , T. Sams , Biochemistry 2013, 52, 4433. 23713667 10.1021/bi400315s

[34] S. E. Maddocks , P. C. F. Oyston , Microbiology (Reading, England) 2008, 154, 3609. 10.1099/mic.0.2008/022772-0 19047729

[35] A. R. Khan , J. C. Parrish , M. E. Fraser , W. W. Smith , P. A. Bartlett , M. N. James , Biochemistry 1998, 37, 16839. 9836576 10.1021/bi9821364

[36] G. W. Lau , D. J. Hassett , H. Ran , F. Kong , Trends Mol. Med. 2004, 10, 599. 15567330 10.1016/j.molmed.2004.10.002

[37] C. C. Caldwell , Y. Chen , H. S. Goetzmann , Y. Hao , M. T. Borchers , D. J. Hassett , L. R. Young , D. Mavrodi , L. Thomashow , G. W. Lau , The American Journal of Pathology 2009, 175, 2473. 19893030 10.2353/ajpath.2009.090166 PMC2789600

[38] M. P. Storz , G. Allegretta , B. Kirsch , M. Empting , R. W. Hartmann , Org. Biomol. Chem. 2014, 12, 6094. 24909330 10.1039/c4ob00707g

[39] F. Lépine , S. Milot , E. Déziel , J. He , L. G. Rahme , J. Am. Soc. Mass Spectrom. 2004, 15, 862. 15144975 10.1016/j.jasms.2004.02.012

[40] Z. A. Machan , G. W. Taylor , T. L. Pitt , P. J. Cole , R. Wilson , The Journal of Antimicrobial Chemotherapy 1992, 30, 615. 1493979 10.1093/jac/30.5.615

[41] D. Hughes , D. I. Andersson , Nature reviews. Genetics 2015, 16, 459. 10.1038/nrg3922 26149714

[42a] R. C. Allen , R. Popat , S. P. Diggle , S. P. Brown , Nature reviews. Microbiology 2014, 12, 300; 24625893 10.1038/nrmicro3232

[42b] A. Ross-Gillespie , R. Kümmerli , Evolution, medicine, and public health 2014, 2014, 134. 10.1093/emph/eou020 PMC4202175 25125554

[43] W. Kabsch , Acta Crystallogr. Sect. D 2010, 66, 125. 20124692 10.1107/S0907444909047337 PMC2815665

[44] P. R. Evans , G. N. Murshudov , Acta Crystallogr. Sect. D 2013, 69, 1204. 23793146 10.1107/S0907444913000061 PMC3689523

[45] M. D. Winn , C. C. Ballard , K. D. Cowtan , E. J. Dodson , P. Emsley , P. R. Evans , R. M. Keegan , E. B. Krissinel , A. G. W. Leslie , A. McCoy , Acta Crystallogr. Sect. D 2011, 67, 235. 21460441 10.1107/S0907444910045749 PMC3069738

[46] I. J. Tickle, C. Flensburg, P. Keller, W. Paciorek, A. Sharff, C. Vonrhein, G. Bricogne, *STARANISO (*http://staraniso.globalphasing.org/cgi-bin/staraniso.cgi*)*, Cambridge, United Kingdom: Global Phasing Ltd., **2018**.

[47] A. J. McCoy , R. W. Grosse-Kunstleve , P. D. Adams , M. D. Winn , L. C. Storoni , R. J. Read , J. Appl. Crystallogr. 2007, 40, 658. 19461840 10.1107/S0021889807021206 PMC2483472

[48] A. A. Vagin , R. A. Steiner , A. A. Lebedev , L. Potterton , S. McNicholas , F. Long , G. N. Murshudov , Acta Crystallogr. Sect. D 2004, 60, 2184. 15572771 10.1107/S0907444904023510

[49] P. Emsley , B. Lohkamp , W. G. Scott , K. Cowtan , Acta Crystallogr. Sect. D 2010, 66, 486. 20383002 10.1107/S0907444910007493 PMC2852313

[50] P. V. Afonine , R. W. Grosse-Kunstleve , N. Echols , J. J. Headd , N. W. Moriarty , M. Mustyakimov , T. C. Terwilliger , A. Urzhumtsev , P. H. Zwart , P. D. Adams , Acta Crystallogr. Sect. D 2012, 68, 352. 22505256 10.1107/S0907444912001308 PMC3322595

[51] P. D. Adams , P. V. Afonine , G. Bunkóczi , V. B. Chen , I. W. Davis , N. Echols , J. J. Headd , L.-W. Hung , G. J. Kapral , R. W. Grosse-Kunstleve , Acta Crystallogr. Sect. D 2010, 66, 213. 20124702 10.1107/S0907444909052925 PMC2815670

