# Supplementary material for: Flexible Fragment Growing Boosts Potency of Quorum‐Sensing Inhibitors against Pseudomonas aeruginosa Virulence
Source: ChemMedChem. 2019 Nov 28;15(2):188–94. doi: 10.1002/cmdc.201900621 (PMC7004148; doi:10.1002/cmdc.201900621)
Supplement: Supplementary file 1 — Supplementary [file CMDC-15-188-s001.pdf]

CHEMMEDCHEM

Supporting Information

© Copyright Wiley-VCH Verlag GmbH & Co. KGaA, 69451 Weinheim, 2020

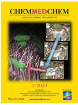

# Flexible Fragment Growing Boosts Potency of Quorum-Sensing Inhibitors against *Pseudomonas aeruginosa* Virulence

Michael Zender, Florian Witzgall, Alexander Kiefer, Benjamin Kirsch, Christine K. Maurer, Andreas M. Kany, Ningna Xu, Stefan Schmelz, Carsten Börger, Wulf Blankenfeldt, and Martin Empting*© 2019 The Authors. Published by Wiley-VCH Verlag GmbH & Co. KGaA. This is an open access article under the terms of the Creative Commons Attribution License, which permits use, distribution and reproduction in any medium, provided the original work is properly cited.

# Content

1. Chemical and Analytical Methods. ..... 3
2. Ligand Interaction Studies ..... 16
3. Isothermal Titration Calorimetry ..... 19
3.1 Site-directed Mutagenesis: $PqsR_{T265A}$ mutant ..... 19
3.2 Expression and Purification of $H_6SUMOPqsR^{c87}$ ..... 19
3.3 Titration Procedure ..... 19
3.4 Thermodynamic profiles of 11 binding to $PqsR_{wt}$ and $PqsR_{T265A}$ ..... 20
3.5 Representative ITC titration curves ..... 21
3.6 Thermodynamic profiles of selected compounds ..... 21
4. X-Ray Crystallography ..... 23
4.1 Expression and Purification of $PqsR_{91-319}$. ..... 23
4.2 Crystallization conditions and data analysis ..... 23
5. *E. coli* reporter-gene assay & dose-response curves ..... 30
6. Effects in *P. aeruginosa* ..... 32
7. Synthesis and Characterization of Intermediates ..... 34
7.1 Synthesis of intermediates 4a-4g ..... 34
7.2 Synthesis of protected precursors 15a and 16a ..... 38
7.3 Synthesis of intermediates 19a, 20a and 21a ..... 40
7.4 Synthesis of intermediates 22a and 22b ..... 42
8. High Resolution Mass Spectroscopy ..... 44
9. Purity of Final Compounds (LC/MS Determination) ..... 44
10. UV traces HPLC ..... 46
11. $^1$H NMR spectra ..... 53
12. References ..... 67

# 1. Chemical and Analytical Methods.

$^{1}$H and $^{13}$C NMR spectra were recorded as indicated on a Bruker DRX-500 or a Bruker Fourier 300 instrument. Chemical shifts are given in parts per million (ppm), and referenced against the residual solvent peak. Coupling constants (*J*) are given in hertz. Low resolution mass analytics and purity control of final compounds was carried out either using a SpectraSystems-MSQ LCMS system (Thermo Fisher Scientific) consisting of a pump, an autosampler, VWD detector and a ESI quadrupole mass spectrometer or a Waters LCMS-Sytsem consisting of a 767 sample Manager, a 2545 binary gradient pump, a 2998 PDA detector and a 3100 electron spray mass spectrometer. High resolution mass spectra were recorded on a maXis 4G hr-ToF mass spectrometer (Bruker Daltonics). Reagents were used as obtained from commercial suppliers without further purification. Procedures were not optimized regarding yield. Microwave assisted synthesis was carried out in a Discover microwave synthesis system (CEM). Column chromatography was performed using the automated flash chromatography system CombiFlash Rf 150 (Teledyne Isco) equipped with RediSepRf silica columns. Final products were dried at high vacuum.

**Chemistry.** Compounds **3-8** (Table 1) were obtained from commercial suppliers. The synthesis to access the key intermediate **4e** is outlined in Scheme 1. The de-novo construction of the 2,4,6-substituted pyridine ring followed a modified protocol described by Adam et al.[1] starting with the acetylation of ethyl-3-amino-4,4,4-trifluorocrotonate with ethyl-malonyl-chloride.

Ring closing was achieved using potassium *tert*-butoxide to obtain **4a**. Hydrolysis and decarboxylation of **4a** by hydrochloric acid and reflux provided **4b**. **4b** was reacted with triflic

anhydride to give **4c**, which was further converted into the corresponding 2,4-diiodo-pyridine (**4d**) by substitution with iodide after protonation of the pyridine with triflic acid. Both steps can also be performed as a one-pot procedure.[2]

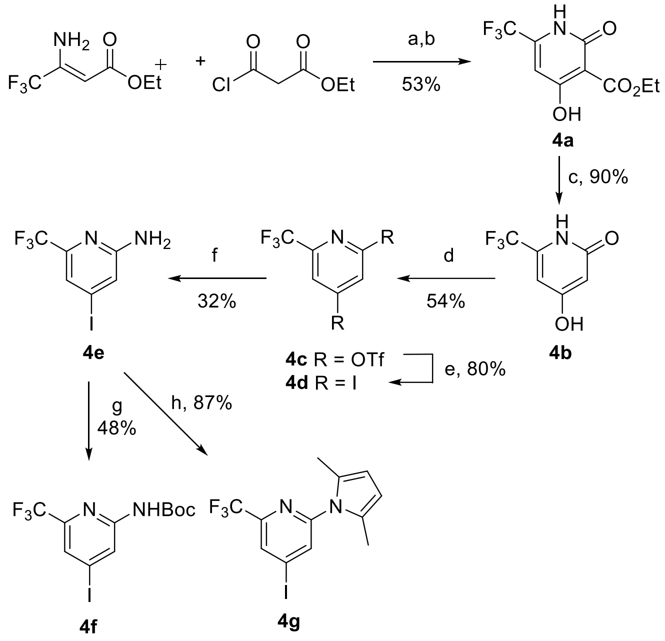

**Scheme S1.** Synthesis of key intermediates. Reagents and Conditions: (a) Pyridine, DCM, RT; (b) potassium *tert*-butoxide, EtOH, 70°C to RT; (c) 6 M HCl, RF; (d) Triflic anhydride, pyridine, acetonitrile 5°C to RT (e) KI, triflic acid, RT; (f) $NH_3$ aq, 1,10-phenanthroline, $Cu_2O$, DMSO, 15°C to RT (f) Boc anhydride, $Et_3N$, DMAP, *t*-BuOH; 35°C (g) hexane-2,5-dione, *p*TsOH, toluene, RF.

For the introduction of the amino group in position two of the pyridine ring, aqueous ammonia and copper/phenanthroline as catalyst were applied, which yielded compound **4e** in 65% isolated yield, while the reaction provided a 84:16 regioselectivity for the desired product. Additionally, the protected precursors **4f** and **4g** were synthesized by standard protocols. Key building block **4e** was suitable for palladium- and copper-catalyzed coupling reactions as shown in Scheme 2. **4e** and 4-fluorophenylboronic acid were reacted under standard Suzuki coupling

conditions using Pd(dppf)$Cl_2$ as catalyst to obtain **9**. **4e** and cyclopropylacetylene were coupled by modified Sonogashira coupling protocol[3] using a combination of $Pd(PPh_3)_2Cl_2$ and TBAF to give **10**. The introduction of the particular amines was achieved using Buchwald-Hartwig conditions. 2-amino pyridines are known to be challenging substrates for most palladium-catalyst reactions. Hence various protocols had to be tested. However, the procedure of Marion et al.[4] using a Pd NHC catalyst allowed us to gain access to amines **11-14** as well as **17** and **18**. Compound **22** was synthesized via a copper-catalyzed amination reaction.[5]

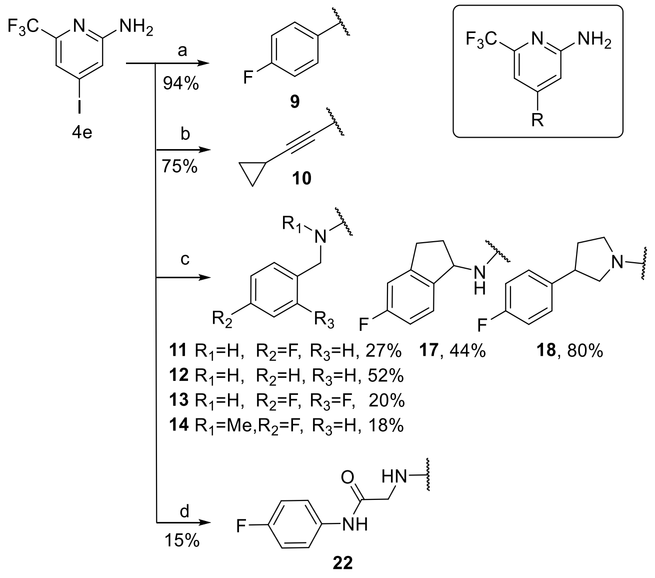


**Scheme S2.** Reactions applied for fragment growing. Reagents and Conditions: (a) (4-fluorophenyl) boronic acid, Pd(dppf)$Cl_2$, $Na_2CO_3$, DME/$H_2O$ 1:1 (v/v), 80°C; (b) cyclo-propylacetylene, $Pd(PPh_3)_2Cl_2$, TBAF (neat), 80°C; (c) amine, (SIPR)Pd(cinnamyl)Cl, KO*t*Bu, DME, 80°C (d) 2-amino-N-(4-fluorophenyl)acetamide, CuI, dimethylaminoethanol, $K_3PO_4$, ACN/$H_2O$ (2/1), 90°C.

As outlined in Scheme 3, the benzyl alcohol was attached to **4g** by SNAr reaction using sodium hydride as base to give **15a**. Deprotection was achieved using hydroxylamine hydrochloride and triethylamine to afford **15**.[6] The synthesis of **16** required the use of the Boc-

protected precursor **4f** and modified Buchwald-Hartwig conditions. **16** was obtained following a standard deprotection procedure using TFA/DCM. Compounds **19-21** were not accessible with the Pd NHC catalyst mentioned before. The particular amines were introduced to **4d** by a microwave assisted SNAr reaction (Scheme 4). Copper-mediated amination of the aryl iodines (**19a-21a**) led to final products (**19-21**).

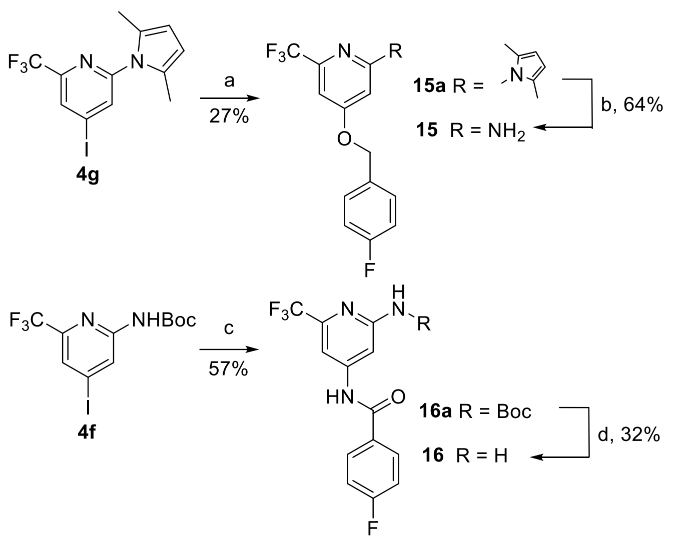


**Scheme S3.** Synthesis of **15** and **16**. Reagents and Conditions: (a) (4-fluorphenyl)methanol, NaH, DMF, 50°C (b) hydroxylamine·HCl, $Et_3N$, $EtOH/H_2O$, RF (c) 4-fluorobenzamide, $Cs_2CO_3$; $Pd_2(dba)_3$, Xantphos, dioxane, 80°C (d) TFA, DCM, 0°C to RT.

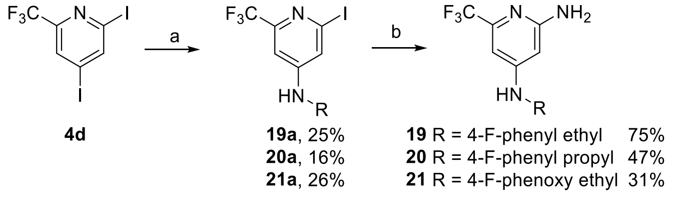


**Scheme S4.** Synthetic route to compounds **19-21**. Reagents and Conditions: (a) amine, Huenig's base, ACN, MW 130°C; (b) $NH_3$ aq, 1,10-phenanthroline, $Cu_2O$, $NMP/H_2O$ RT.

**Method A: General Procedure for Buchwald-Hartwig cross coupling reactions.**[7] Heteroaryl halide (0.35 mmol), potassium tert-butoxide (1.1 mmol) and (SIPr)Pd(cinnamyl)Cl

(3 mol%) were combined into MW-vial (Biotage). The vial was evacuated and flushed with argon (3 times). Dry dimethoxyethane (1 ml) was added. The reaction mixture was purged with argon and the amine (1.1 mmol) was slowly added. The reaction was stirred over night at 80°C. The mixture quenched with diethyl ether, filtered through Cellite and the filtrate was concentrated to dryness. The crude was further purified by preparative HPLC or chromatography on silica gel.

**Method B: Copper-mediated amination reaction.** 2-Iodo-6-(trifluoromethyl)pyridin-4-amine (0.3 mmol) was dissolved in NMP/water (3ml; 2/1(V/V)) using crimp reaction vial. (Biotage). 1,10-phenanthroline (0.1 mmol) and copper (I) oxide (0.1 mmol) were added. The reaction mixture was purged with argon and $NH_{3(aq)}$ 28% (~5 mmol) was added. The mixture was stirred over night at RT. The crude product was purified by automated flash chromatography.

**4-(cyclopropylethynyl)-6-(trifluoromethyl)pyridin-2-amine (9).** 4-Iodo-6-(trifluoromethyl)pyridin-2-amine (**4e**; 100 mg, 0.415 mmol), $PdCl_2(PPh_3)_2$ (7 mg, 10 µmol), tetrabutylammonium fluorid (1.0 mmol) and ethynylcyclopropane (34 mg, 0.52 mmol) were filled into a crimp vial (neat reation). The vial was evacuated and purged with argon (3 times). The reaction mixture was heated at 80°C overnight. The reaction was quenched by addition of water. The aqueous layer was extracted with ethylacetat (3 times). Combined organics were washed with brine, dried over $MgSO_4$ and concentrated. The crude product was purified by automated flash chromatography (PE:EA 95:5 to PE:EA 80:20) to give the titled compound (74 mg, 0.33 mmol, 94 % yield) as pale yellow solid. $^1H$ NMR (500 MHz, CHLOROFORM-*d*) δ ppm 0.82 - 0.87 (m, 2 H), 0.91 - 0.96 (m, 2 H), 1.42 - 1.48 (m, 1 H), 4.75 (br. s., 2 H), 6.59 (d,

J=0.6 Hz, 1 H), 6.95 (d, J=0.9 Hz, 1 H); $^{13}$C NMR (126 MHz, CHLOROFORM-*d*) δ ppm 1.00, 8.94 (2 C), 73.19, 99.37, 112.76 (d, $^{3}J(C,F)$=3.7 Hz, 1 C), 113.34, 121.35 (q, $^{1}J(C,F)$=274.0 Hz, 1 C), 134.77, 146.53 (q, $^{2}J(C,F)$=33.9 Hz, 1 C), 158.46; MS (ESI+) *m/z* 268 (M+ACN+H)$^{+}$.

**(4-fluorophenyl)-6-(trifluoromethyl)pyridin-2-amine (10).** 4-Iodo-6-(trifluoromethyl)pyridin-2-amine (**4e**; 100 mg, 0.35 mmol), (4-fluorophenyl)boronic acid (73 mg, 0.52 mmol) and Pd(dppf)$Cl_2$ (12 mg, 0.016 mmol) were filled into a crimp reaction vial. The vial was evacuated and flushed with argon (2 times). $Na_2CO_3$ (3 ml, 3.0 mmol) 1M solution were added. The mixture was purged with argon and stirred at 80°C for overnight. The mixture was quenched by addition of water and extracted by ether. The combined organics were washed with brine, dried over $MgSO_4$ and concentrated. The crude was attached purified by automated flash chromatography (PE to PE:EA 80:20) to give the titled compound (67 mg, 0,262 mmol, 75 % yield) as a white solid. $^{1}$H NMR (300 MHz, CHLOROFORM-*d*) δ ppm 4.92 (br. s., 2 H), 6.78 (s, 1 H), 7.07 - 7.24 (m, 3 H), 7.49 - 7.65 (m, 2 H); $^{13}$C NMR (75 MHz, CHLOROFORM-*d*) δ ppm 108.89, 108.92 (q, $^{3}J(C,F)$=3.0 Hz, 1 C), 116.16 (d, $^{2}J(C,F)$=22.4 Hz, 2 C), 121.61 (q, $^{1}J(C,F)$=274.2 Hz, 1 C), 128.74 (d, $^{3}J(C,F)$=8.9 Hz, 2 C), 133.80 (d, $^{4}J(C,F)$=3.7 Hz, 1 C), 147.24 (q, $^{2}J(C,F)$=33.5 Hz, 1 C), 150.51, 159.12, 163.60 (d, $^{2}J(C,F)$=249.6 Hz, 1 C); MS (ESI+) m/z 257 (M+H)$^{+}$; 298 (M+ACN+H)$^{+}$.

***N*4-(4-fluorobenzyl)-6-(trifluoromethyl)pyridine-2,4-diamine (11)** was synthesized according to Method A from 4-iodo-6-(trifluoromethyl)pyridin-2-amine (**4e**; 100 mg, 0.35 mmol) and (4-fluorophenyl)methanamine (130 mg, 1.04 mmol). The crude was purified by combyflash (PE:dieethylether 80:20 to 60:40) to give a pale brown solid. The solid was titurated with diethyl ether/pentane and filtered to give the titled compound (27 mg, 0.095 mmol, 27%

yield) as a brown-white solid. $^{1}$H NMR (500 MHz, CHLOROFORM-*d*) δ ppm 4.33 (d, *J*=5.4 Hz, 2 H), 4.46 (br. s., 2 H), 4.56 (br. s., 1 H), 5.68 (d, *J*=1.6 Hz, 1 H), 6.37 (d, *J*=1.9 Hz, 1 H), 7.00 - 7.12 (m, 2 H), 7.20 - 7.35 (m, 2 H); $^{13}$C NMR (126 MHz, CHLOROFORM-*d*) δ ppm 46.17, 90.66, 98.52, 115.56 (d, $^{2}J(C,F)$=21.1 Hz, 2 C), 121.36 (d, $^{1}J(C,F)$=273.1 Hz, 1 C), 128.62 (d, $^{3}J(C,F)$=8.2 Hz, 2 C), 132.83 (d, $^{4}J(C,F)$=3.7 Hz, 1 C), 146.65 (q, $^{2}J(C,F)$=33.0 Hz, 1 C), 154.83, 159.57, 162.04 (d, $^{1}J(C,F)$=246.5 Hz, 1 C); MS (ESI+) *m/z* 286 $(M+H)^{+}$.

***N*4-benzyl-6-(trifluoromethyl)pyridine-2,4-diamine (12)** was synthesized according to Method A from 4-iodo-6-(trifluoromethyl)pyridin-2-amine (**4e**; 100 mg, 0.35 mmol) and phenylmethanamine (112 mg, 1.04 mmol). The crude product was purified by combyflash (PE:diethylether gradient, elution 30:70 PE:ether) to give the titled compound as pale yellow solid (48 mg, 0.18 mmol, 52% yield). $^{1}$H NMR (500 MHz, CHLOROFORM-*d*) δ ppm 4.34 (d, J=5.7 Hz, 2 H), 4.43 - 4.93 (m, 1 H), 5.68 (d, *J*=1.9 Hz, 1 H), 6.35 (d, *J*=1.9 Hz, 1 H), 7.28 - 7.34 (m, 3 H), 7.34 - 7.41 (m, 2 H); $^{13}$C NMR (126 MHz, CHLOROFORM-*d*) δ ppm 47.01, 90.83, 98.68 (d, $^{3}J(C,F)$=3.7 Hz, 1 C), 121.71 (q, $^{1}J(C,F)$=274.9 Hz, 1 C), 127.21 (2 C), 127.68, 128.84 (2 C), 137.48, 146.78 (q, $^{2}J(C,F)$=33.0 Hz, 1 C), 155.20, 159.85; MS (ESI+) m/z 259 $(M+H)^{+}$ 299 $(M+ACN+H)^{+}$

***N*4-(2,4-difluorobenzyl)-6-(trifluoromethyl)pyridine-2,4-diamine (13)** was synthesized according to Method A from 4-iodo-6-(trifluoromethyl)pyridin-2-amine (**4e**; 100 mg, 0.35 mmol) and (2,4-difluorophenyl)methanamine (149 mg, 1.04 mmol). The crude product was purified by preparative HPLC to give the titled compound (21 mg, 0,069 mmol, 20 % yield) as brown oil. $^{1}$H NMR (500 MHz, METHANOL-$d_4$) δ ppm 4.37 (s, 2 H), 5.78 (d, *J*=1.6 Hz, 1 H), 6.42 (d, *J*=1.9 Hz, 1 H), 6.87 - 7.02 (m, 2 H), 7.36 (td, *J*=8.2, 6.6 Hz, 1 H); $^{13}$C NMR (126

MHz, METHANOL-$d_4$) δ ppm 40.61 (d, $^3J(C,F)$=4.6 Hz, 1 C), 90.70, 99.84, 104.68 (t, $^2J(C,F)$=25.7 Hz, 1 C), 112.30 (dd, $^2J(C,F)$=20.2, 3.7 Hz, 1 C), 123.07 (q, $^1J(C,F)$=273.1 Hz, 1 C), 122.55 (dd, $^3J(C,F)$=14.7, 3.7 Hz, 1 C), 131.47 (dd, $^3J(C,F)$=9.6, 6.0 Hz, 1 C), 145.77 (q, $^2J(C,F)$=33.9 Hz, 1 C), 157.12, 162.26 (dd, $^1J(C,F)$=243.8, 11.9 Hz, 1 C), 161.74, 163.84 (dd, $^1J(C,F)$=246.5, 12.8 Hz, 1 C); MS (ESI +) *m/z* 304 $(M+H)^+$.

**N4-(4-fluorobenzyl)-N4-methyl-6-(trifluoromethyl)pyridine-2,4-diamine (14)** was synthesized according to Method A from 4-iodo-6-(trifluoromethyl)pyridin-2-amine (**4e**; 100 mg, 0.35 mmol) and 1-(4-fluorophenyl)-N-methylmethanamine (145 mg, 1,042 mmol). The crude product was purified by automated flash chromatography using a gradient of Hex:EA (8:2 to 6:4) to give the titled compound (19 mg, 0.063 mmol, 18% yield) as white solid. $^1$H NMR (300 MHz, CHLOROFORM-*d*) δ ppm 3.04 (s, 3 H), 4.52 (s, 4 H), 5.76 (d, *J*=2.0 Hz, 1 H), 6.48 (d, *J*=2.1 Hz, 1 H), 6.97 - 7.07 (m, 2 H), 7.08 - 7.16 (m, 2 H); $^{13}$C NMR (75 MHz, CHLOROFORM-*d*) δ ppm 37.94, 54.57, 90.92, 96.73 (d, $^3J(C,F)$=3.7 Hz, 1 C), 115.80 (d, $^2J(C,F)$=20.9 Hz, 1 C), 121.88 (q, $^1J(C,F)$=274.2 Hz, 1 C), 127.99 (d, $^3J(C,F)$=8.2 Hz, 2 C), 132.48 (d, $^4J(C,F)$=3.7 Hz, 1 C), 147.34 (q, $^2J(C,F)$=32.8 Hz, 1 C), 156.08, 159.78, 162.14 (d, $^1J(C,F)$=245.9 Hz, 1 C); MS (ESI +) m/z 300 $(M+H)^+$.

**4-((4-fluorobenzyl)oxy)-6-(trifluoromethyl)pyridin-2-amine (15).** 2-(2,5-Dimethyl-1H-pyrrol-1-yl)-4-((4-fluorobenzyl)oxy)-6-(trifluoromethyl)pyridine (**15a**; 80 mg, 0.22 mmol), triethylamine (0.061 ml, 0.44 mmol) and hydroxylamine hydrochloride (153 mg, 2.20 mmol) were dissolved in EtOH/water 3/1 and the solution was refluxed overnight. An additional amount of hydroxylamine hydrochloride (153 mg, 2.20 mmol) and triethylamine (0.061 ml, 0.44 mmol) was added and again refluxed overnight. The mixture was quenched by addition of

EA and water. The aqueous phase was extracted (EA 3 times). Combined organcis were washed with brine, dried ($MgSO_4$) filtered and concentrated. The crude was purified by column chromatography (step gradient 9:1 to 1:1 PE:EA) to give the titled compound (40 mg, 0.14 mmol, 64 % yield) as pale yellow crystals. $^1$H NMR (300 MHz, DMSO-$d_6$) δ ppm 5.12 (s, 2 H), 6.22 (d, *J*=1.8 Hz, 1 H), 6.44 (s, 2 H), 6.59 (d, *J*=2.0 Hz, 1 H), 7.13 - 7.33 (m, 2 H), 7.50 (dd, *J*=8.7, 5.5 Hz, 1 H); $^{13}$C NMR (75 MHz, DMSO-$d_6$) δ ppm 68.58, 94.36, 98.49 (d, $^4J(C,F)$=3.0 Hz, 1 C), 115.31 (d, $^2J(C,F)$=22.4 Hz, 2 C), 121.61 (q, $^1J(C,F)$=274.2 Hz, 1 C), 130.13 (d, $^3J(C,F)$=8.2 Hz, 2 C), 132.21 (d, $^4J(C,F)$=3.0 Hz, 1 C), 146.25 (q, $^2J(C,F)$=33.5 Hz, 1 C), 161.89 (d, $^1J(C,F)$=244.4 Hz, 1 C), 161.80, 165.70; MS (ESI+) m/z 287 (M+H)+; 328 $(M+ACN+H)^+$.

**N-(2-amino-6-(trifluoromethyl)pyridin-4-yl)-4-fluorobenzamide (16).** Tert-butyl (4-(4-fluorobenzamido)-6-(trifluoromethyl)pyridin-2-yl)carbamate (**16a**; 46 mg, 0.11 mmol) was dissolved in DCM (4 ml) and cooled on an ice bath. Trifluoroacetic acid (1 ml) was added and the mixture was aged at RT for 3 h. The reaction was quenched by addition of DCM and $Na_2CO_3$ saturated solution. The aqueous layer was extracted with DCM (3 times) and EtOAc (3 times). Both organics were dried over $MgSO_4$, filtered and concentrated. The crude was purified by automated flash chromatography (PE:EA 75:25) to give the titled compound (11 mg, 0.04 mmol, 32% yield) as white solid. $^1$H NMR (300 MHz, DMSO-$d_6$) δ ppm 6.53 (br. s, 2 H), 7.26 - 7.33 (m, 2 H), 7.33 - 7.44 (m, 2 H), 7.93 - 8.12 (m, 2 H), 10.50 (br. s, 1 H); $^{13}$C NMR (75 MHz, DMSO-$d_6$) δ ppm 100.07, 101.06 (q, $^3J(C,F)$=3.0 Hz, 1 C), 115.93 (d, $^2J(C,F)$=21.6 Hz, 2 C), 122.28 (q, $^1J(C,F)$=273.4 Hz, 1 C), 131.06, 131.13 (d, $^3J(C,F)$=8.9 Hz, 1 C), 145.91 (q,

${}^{2}J(C,F)$=32.0 Hz, 1 C), 147.88, 161.61, 164.84 (d, ${}^{1}J(C,F)$=248.9 Hz, 1 C), 165.77; MS (ESI+) m/z 300 $(M+H)^{+}$, 341 $(M+ACN+H)^{+}$

**N4-(5-fluoro-2,3-dihydro-1H-inden-1-yl)-6-(trifluoromethyl)pyridine- 2,4-diamine (17)** was synthesized according to Method A from 4-iodo-6-(trifluoromethyl)pyridin-2-amine (**4e**; 100 mg, 0.35 mmol) and and 5-fluoro-2,3-dihydro-1H-inden-1-amine (157 mg, 1.04 mmol). The crude product was purified by preparative HPLC to give the titled compound (48 mg, 0.15 mmol, 44 % yield) as brown solid. $^{1}$H NMR (300 MHz, CHLOROFORM-*d*) δ ppm 1.87 - 2.08 (m, 1 H), 2.53 - 2.69 (m, 1 H), 2.80 - 3.11 (m, 2 H), 4.41 (d, J=7.8 Hz, 1 H), 4.53 (br. s., 2 H), 4.97 (q, J=6.9 Hz, 1 H), 5.83 (d, *J*=1.8 Hz, 1 H), 6.37 (d, *J*=1.9 Hz, 1 H), 6.82 - 7.04 (m, 2 H), 7.16 - 7.37 (m, 1 H); $^{13}$C NMR (75 MHz, CHLOROFORM-*d*) δ ppm 30.21, 33.84, 57.04, 91.04, 98.78 (d, ${}^{3}J(C,F)$=3.7 Hz, 1 C), 112.03 (d, ${}^{2}J(C,F)$=23.1 Hz, 1 C), 114.05 (d, ${}^{2}J(C,F)$=23.1 Hz, 1 C), 121.70 (d, ${}^{1}J(C,F)$=273.4 Hz, 1 C), 125.28 (d, ${}^{3}J(C,F)$=8.9 Hz, 1 C), 138.39 (d, ${}^{4}J(C,F)$=2.2 Hz, 1 C), 145.89 (d, ${}^{3}J(C,F)$=8.2 Hz, 1 C), 147.33 (q, ${}^{2}J(C,F)$=32.0 Hz, 1 C), 154.73, 159.80, 163.24 (d, ${}^{1}J(C,F)$=245.9 Hz, 1 C); MS (ESI+) m/z 312 (M+H)+.

**4-(3-(4-fluorophenyl)pyrrolidin-1-yl)-6-(trifluoromethyl)pyridin-2-amine (18)** was synthesized according to Method A from 4-iodo-6-(trifluoromethyl)pyridin-2-amine (**4e**, 100 mg, 0.35 mmol) and and 3-(4-fluorophenyl)pyrrolidine (172 mg, 1.04 mmol). The crude product was purified by automated flash chromatography (PE:Diethylether gradient) to give the titled compound (90 mg, 0.28 mmol, 80 % yield) as pale yellow solid. $^{1}$H NMR (300 MHz, CHLOROFORM-*d*) δ ppm 2.02 - 2.21 (m, 1 H), 2.43 (m, 1 H), 3.33 (t, J=9.0 Hz, 1 H), 3.38 - 3.61 (m, 3 H), 3.74 (dd, J=9.3, 7.7 Hz, 1 H), 4.49 (br. s., 2 H), 5.65 (d, J=1.8 Hz, 1 H), 6.34 (d, J=1.9 Hz, 1 H), 6.93 - 7.11 (m, 2 H), 7.14 - 7.26 (m, 2 H); $^{13}$C NMR (75 MHz, CHLOROFORM-

*d*) δ ppm 32.47, 42.64, 46.70, 53.42, 90.23 (d, *J*=6.0 Hz, 1 C), 96.76 (d, $^{3}J(C,F)$=3.7 Hz, 1 C), 115.11 (d, $^{2}J(C,F)$=18.6 Hz, 2 C), 121.49 (d, $^{1}J(C,F)$=274.2 Hz, 1 C), 128.03 (d, J=7.4 Hz, 2 C), 136.52 (d, $^{3}J(C,F)$=3.7 Hz, 1 C), 146.63 (q, $^{2}J(C,F)$=32.8 Hz, 1 C), 153.15, 158.97, 161.37 (d, $^{1}J(C,F)$=245.1 Hz, 1 C); MS (ESI+) m/z 326 $(M+H)^{+}$, 367 $(M+ACN+H)^{+}$

**N4-(4-fluorophenethyl)-6-(trifluoromethyl)pyridine-2,4-diamine (19)** was synthesized according to Method B from N-(4-fluorophenethyl)-2-iodo-6-(trifluoromethyl)- pyridin-4-amine (100 mg, 0.24 mmol). The crude product was purified by automated flash chromatography (PE:DE 7:3 to 3:7) to give the titled compound (55 mg, 0.18 mmol, 75 % yield) as white solid.$^{1}$H NMR (500 MHz, DMSO-$d_6$) δ ppm 2.80 (t, *J*=7.4 Hz, 2 H), 3.18 - 3.26 (m, 2 H), 5.72 (d, *J*=1.6 Hz, 1 H), 5.95 (br. s., 2 H), 6.27 (d, *J*=1.6 Hz, 1 H), 6.67 (br. s., 1 H), 7.08 - 7.16 (m, 2 H), 7.27 - 7.34 (m, 2 H); $^{13}$C NMR (126 MHz, DMSO-d6) δ ppm 33.43, 43.65, 88.92, 97.12, 115.02 (d, $^{2}J(C,F)$=20.2 Hz, 2 C), 122.09 (q, $^{1}J(C,F)$=274.0 Hz, 1 C), 130.53 (d, $^{3}J(C,F)$=8.2 Hz, 2 C), 135.51 (d, $^{3}J(C,F)$=2.8 Hz, 1 C), 144.59 (q, $^{2}J(C,F)$=30.2 Hz, 1 C), 154.92, 160.92 (d, $^{1}J(C,F)$=241.9 Hz, 1 C), 160.70; MS (ESI+) *m/z* 300 $(M+H)^{+}$; HRMS found 300.11091 calc. 300.11184 $(M+H)^{+}$.

**N4-(3-(4-fluorophenyl)propyl)-6-(trifluoromethyl)-pyridine-2,4-diamine (20)** was synthesized according to Method B from N-(3-(4-fluorophenyl)propyl)-2-iodo-6-(trifluoromethyl)pyridin-4-amine (130 mg, 0.31 mmol). The crude product was purified by purified by automated flash chromatography (gradient PE:diethylether) to give the titled compound (45 mg, 0.14 mmol, 47 % yield) as pale yellow solid $^{1}$H NMR (500 MHz, DMSO-$d_6$) δ ppm 1.80 (quin, *J*=7.3 Hz, 1 H), 2.65 (t, *J*=7.3 Hz, 2 H), 2.93 - 3.03 (m, 2 H), 5.62 (d, *J*=1.3 Hz, 1 H), 5.88 (s, 2 H), 6.25 (d, *J*=1.9 Hz, 1 H), 6.55 (t, *J*=5.2 Hz, 1 H), 7.07 - 7.14 (m,

2 H), 7.21 - 7.28 (m, 2 H); $^{13}C$ NMR (126 MHz, DMSO-$d_6$) δ ppm 30.00, 31.57, 41.20, 88.82, 96.79, 114.93 (d, $^{2}J(C,F)$=21.1 Hz, 2 C), 122.15 (q, $^{1}J(C,F)$=274.0 Hz, 1 C), 129.97 (d, $^{3}J(C,F)$=7.3 Hz, 2 C), 137.63 (d, $^{3}J(C,F)$=3.7 Hz, 1 C), 144.95 (q, $^{2}J(C,F)J$=32.1 Hz, 1 C), 155.03, 160.58 (d, $^{1}J(C,F)$=241.0 Hz, 1 C), 160.88; MS (ESI+) 314 $(M+H)^+$; HRMS found 314.12708 calc 314.12749 $(M+H)^+$.

**N4-(2-(4-fluorophenoxy)ethyl)-6-(trifluoromethyl)-pyridine-2,4-diamine (21)** was synthesized according to Method B from N-(2-(4-fluorophenoxy)ethyl)-2-iodo-6-(trifluoromethyl)pyridin-4-amine (**21a**; 30,0 mg, 0.07 mmol). The crude product was purified by automated flash chromatography (PE:EA gradient). The isolated product was further purified by preparative HPLC to give the titled compound (7 mg, 0.022 mmol, 31% yield) as white solid. $^{1}H$ NMR (500 MHz, METHANOL-$d_4$) δ ppm 3.52 (t, $J$=5.4 Hz, 2 H), 4.12 (t, $J$=5.5 Hz, 2 H), 5.88 (d, $J$=1.9 Hz, 1 H), 6.41 (d, $J$=1.9 Hz, 1 H), 6.91 - 6.97 (m, 2 H), 6.97 - 7.04 (m, 2 H); $^{13}C$ NMR (126 MHz, METHANOL-$d_4$) δ ppm 43.01, 68.05, 90.74, 99.48, 116.69 (d, $^{2}J(C,F)$=22.9 Hz, 2 C), 116.82 (d, $^{3}J(C,F)$=7.8 Hz, 2 C), 123.34 (q, $^{1}J(C,F)$=273.1 Hz, 1 C), 146.79 (q, $^{2}J(C,F)$=33.0 Hz, 1 C), 156.43 (d, $^{4}J(C,F)$=1.8 Hz, 1 C), 157.39, 158.81 (d, $^{1}J(C,F)$=236.4 Hz, 1 C); MS (ESI+) 316 $(M+H)^+$; HRMS found 316.10667 calc. 316.10675 $(M+H)^+$.

**2-((2-amino-6-(trifluoromethyl)pyridin-4-yl)amino)-N-(4-fluorophenyl)acetamide (22).** 4-Iodo-6-(trifluoro methyl)pyridin-2-amine (**22a**; 150 mg, 0.52 mmol), 2-amino-N-(4-fluorophenyl)acetamide (131 mg, 0.78 mmol), copper (I) iodide (99 mg, 0.52 mmol), $K_3PO_4$ (221 mg, 1.04 mmol) and 2-(dimethylamino)ethanol (46 mg, 0.5 mmol) were filled into a crimp vial. The vial was evacuated and backfilled with argon (3 times). Acetonitrile (4 ml) and water

(2 ml) were added and the mixture degassed with argon .The reaction was heated at 90 °C over night. The mixture was poured into saturated $NH_4Cl$ solution. The aqueous phase was extracted using EtOAc (3 times). The combined organics were washed with brine, dried ($MgSO_4$), filtered and adsorbed onto silica gel. The crude product was first purified by automated flash chromatography (PE:EA gradient) and further by prep HPLC to give the titled compound (25 mg, 0,076 mmol, 15 % yield) as white solid. $^{1}H$ NMR (500 MHz, DMSO-$d_6$) δ ppm 3.89 (d, *J*=6.0 Hz, 2 H), 5.62 (s, 1 H), 5.97 (s, 2 H), 6.37 (d, *J*=1.9 Hz, 1 H), 6.87 (t, *J*=6.1 Hz, 1 H), 7.05 - 7.22 (m, 2 H), 7.54 - 7.66 (m, 2 H), 10.12 (s, 1 H). $^{13}C$ NMR (126 MHz, DMSO-$d_6$) δ ppm 45.79, 89.55, 97.15, 115.28 (d, *J*=20.2 Hz, 2 C), 122.11 (q, $^{1}J(C,F)$=274.0 Hz, 1 C), 121.03 (d, *J*=7.3 Hz, 2 C), 135.14 (d, *J*=2.8 Hz, 1 C), 144.99 (q, *J*=32.1 Hz, 1 C), 155.07, 158.01 (d, $^{1}J(C,F)$=239.2 Hz, 1 C), 160.80, 167.91; MS (ESI+) 329.2 $(M+H)^+$. HRMS found 329.10171 calc. 329.10200 $(M+H)^+$

## 2. Ligand Interaction Studies

Complex structures of HHQ (PDB ID: 6Q7U) as well as compounds **11** (PDB ID: 6Q7V) and **20** (PDB ID: 6Q7W) bound to the ligand-binding domain of PqsR ($PqsR_{91-319}$) were analyzed using Molecular Operating Environment MOE (Chemical Computing Group) after applying the "QuickPrep" protocol using the MMFF94x force field (see Figure S1 in the Supporting Information). Default parameters were used, while receptor, ligands, and solvents were tethered ("strength" parameter of 10). A prominent water molecule observed in PDB ID 6Q7W (compound **20**) was also considered when investigating the interactions of compound **11**. Figures 3 and 4 of the main text were rendered with PovRay.

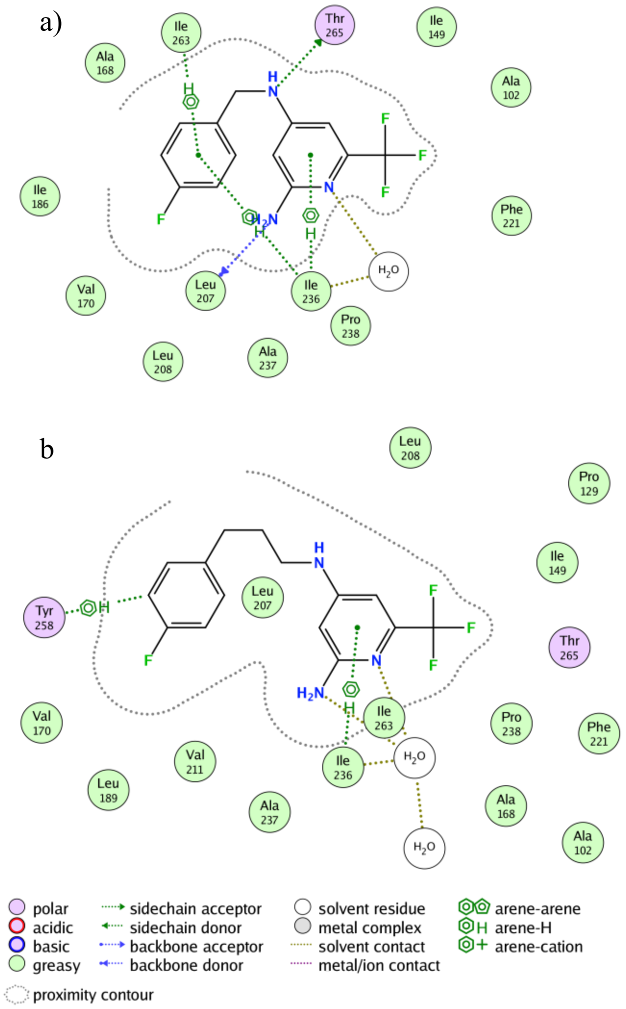


Figure S1: 2D schematic representation of compounds 11 (A) and 20 (B) in complex with $PqsR_{c91}$. Protein-ligand interactions after energy minimization using the "Quickprep" application included in the MOE software package were indicated as described in the legend.

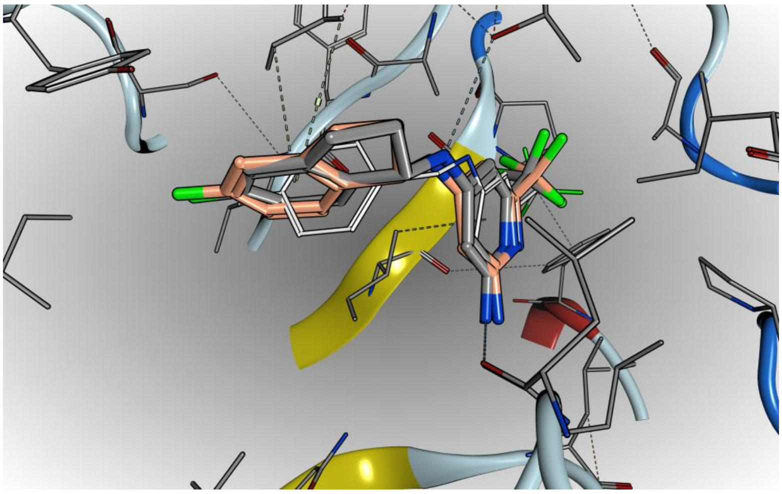

**Figure S2:** Modeled binding pose of compound **17.**

Binding pose of **17** (before energy minimization with LigX rose fat line and after grey fat line) was modeled based on the crystal structure of $PqsR_{c91}$ in complex with **11** (thin white line).

# 3. Isothermal Titration Calorimetry

## 3.1 Site-directed Mutagenesis: $PqsR_{T265A}$ mutant

The plasmid pSUMO3_ck4_T265A*pqsR*$^{C87}$ was generated as previously described[8] using QuikChange Site-Directed Mutagenesis Kit (Stratagene) following the manufacturer's instructions. The following primer were used: forward 5' CGAACCGGGCGGCATCGACGCGAAGGTGTATTGC 3' and reverse 5' GCAATACACCTTCGCGTCGATGCCGCCCGGTTCG 3. PCR condition to amplify the T265A gene: 16 cycles with 35s denaturation at 95 °C, 60 s annealing at 55 °C, and 13 min extension. The generated plasmid was transformed via electroporation into E. coli BL21

## 3.2 Expression and Purification of $H_6SUMOPqsR^{c87}$

$H_6SUMOPqsR^{c87}$ and T265A mutant $H_6SUMOPqsR^{c87}$ for ITC studies was expressed and purified as previously reported.[8,9] Briefly, *E. coli* BL21 (λDE3) transfected with the pSUMO3_ck4_*pqsR*$^{C87}$ or pSUMO3_ck4_T265A*pqsR*$^{C87}$ plasmid[8] were grown in LB medium containing 50 µg mL$^{-1}$ kanamycin at 37 °C to an $OD_{600}$ of approximately 0.8 and expression was induced with 0.2 mM IPTG for 16 h at 16 °C. The protein was purified using nickel affinity chromatography in a one step gradient.

## 3.3 Titration Procedure

ITC titrations were performed as described before.[8,9] Briefly, ITC experiments were carried out using an $ITC_{200}$ instrument (Microcal Inc., GE Healthcare). The titrations were performed on 50-150 µM $H_6SUMO$-$PqsR^{C87}$ in the 200 µL sample cell using 19 injections of 2 µL ligand solution (0.5-1.5 mM) every 180 s. The data were collected and the area under each peak was integrated. To correct for heats of dilution and mixing the final base line consisting of small peaks of the same size at the end of the titration were substracted.

Ligand efficiency (LE) was calculated according to formula 1 and enthalpic efficiency (EE) was calculated according to formula 2:

$LE = -\Delta G/(\text{heavy atom count})$ (1)

$EE = - \Delta H/(\text{heavy atom count})$ (2)

where $\Delta G$ is the change in free energy, $\Delta H$ is the enthalpic contribution and heavy atom count is the number of non-hydrogen atoms of the compound.

## 3.4 Thermodynamic profiles of 11 binding to $PqsR_{wt}$ and $PqsR_{T265A}$

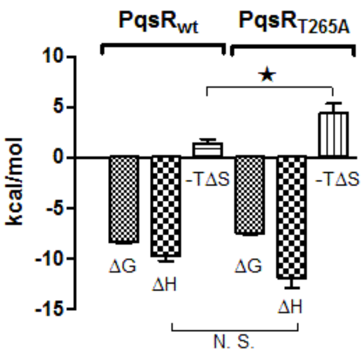


**Figure S3:** Comparison of the thermodynamic profiles of **11** binding to PqsRwt (left part) and $PqsR_{T265A}$ (right part). T-test of ΔH values (PqsRwt/$PqsR_{T265A}$) showed no significant difference ($P > 0.05$). T-test of -TΔS elucidated a significant difference ($P < 0.05$).

## 3.5 Representative ITC titration curves

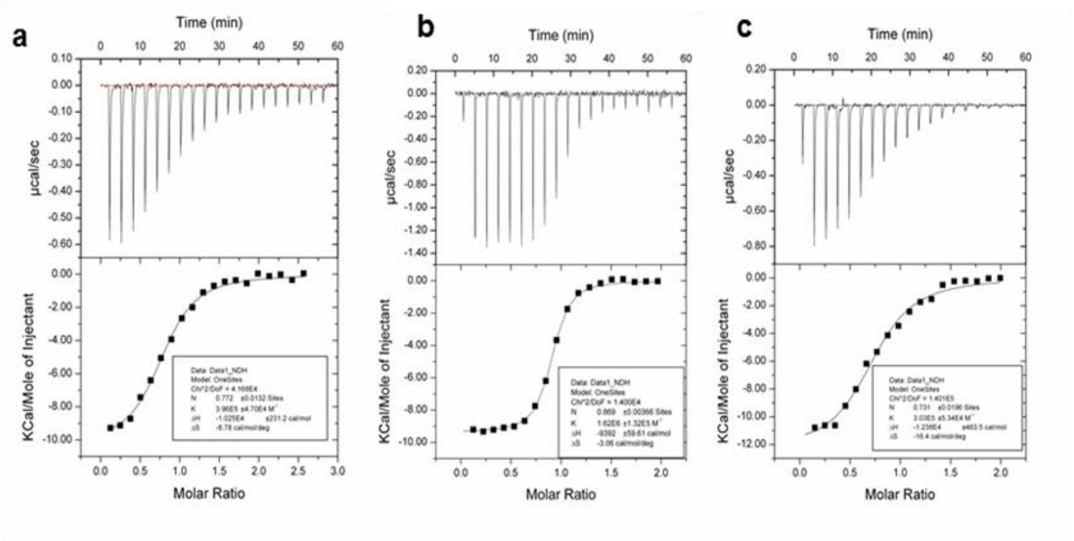


**Figure S4:** Representative ITC titrations against $H_6$SUMO-PqsR$^{C87}$ (50 µM) **a) 7** (500 µM) **b) 11** (500 µM) **c)** (500 µM) **c) 20** (500 µM). The upper row shows the heat change recorded over time in µcal/sec for 19-injections of the ligand. The lower row shows the integrated heats (■) plotted against the molar ratio of the binding reaction. Data were fitted to a 1:1 binding model continuous black line. The continuous line represents the results of the non-linear least squares fitting of the

## 3.6 Thermodynamic profiles of selected compounds

Table S1: Thermodynamic evaluation of selected antagonists

| Comp | $K_{D\ (ITC)}$ [µM] | ΔG [kcal mol$^{-1}$] | ΔH [kcal mol$^{-1}$] | -TΔS [kcal mol$^{-1}$] | EE$^a$ [kcal mol$^{-1}$] | LE$^b$ [kcal mol$^{-1}$] |
|---|---|---|---|---|---|---|
| 9 | 7.5±0.1 | -6.9±0.0 | -3.7±0.6 | -3.8±0.6 | 0.23 | 0.43 |
| 11 | 0.6±0.2 | -8.4±0.2 | -9.8±0.8 | 1.0±0.8 | 0.49 | 0.42 |
| 12 | 1.4±0.5 | -8.1±0.3 | -11.3±0.3 | 3.2±0.5 | 0.57 | 0.43 |
| 13 | 1.3±0.3 | -8.0±0.1 | -11.6±0.9 | 3.5±0.8 | 0.55 | 0.40 |
| 19 | 4.7±2.3 | -7.4±0.3 | -13.5±0.6 | 6.1±0.7 | 0.64 | 0.35 |

| | | | | | | |
|---|---|---|---|---|---|---|
| 20 | 2.8±0.5 | -7.6±0.1 | -11.4±0.9 | 3.8±1.0 | 0.50 | 0.35 |
| 22 | 1.0±0.0 | -8.2±0.2 | -12.9±0.1 | 4.7±0.1 | 0.56 | 0.36 |

ITC titrations were performed at 298 K. Data represent mean ± SD from at least two independent experiments; [a]EE = -Δ*H*/(heavy atom count); [b]LE = –Δ*G*/(heavy atom count)

# 4. X-Ray Crystallography

## 4.1 Expression and Purification of $PqsR_{91\text{-}319}$.

The ligand-binding domain of PqsR comprising residues 91-319 ($PqsR_{91\text{-}319}$) was expressed and purified as reported by Xu *et al.*[10] with some modifications. The plasmid pOPIN-$His_6$-SUMO-$PqsR_{91\text{-}319}$ was transformed into *E. coli* BL21-CodonPlus(DE3)-RIL and protein expression was induced with 0.5 mM IPTG for 16 h at 20°C, when the culture reached an $OD_{600}$ of 2.5. After centrifugation, the cells were resuspended in buffer A (150 mM $Na_2HPO_4$/$NaH_2PO_4$, 300 mM NaCl, pH 8.0) supplemented with a EDTA-free protease inhibitor cocktail tablet (Roche Life Science). The cells were lysed with an Emulsiflex-C3 homogenizer (Avestin) with two cycles and the supernatant was applied onto a HisTrap HP column (GE Healthcare). $His_6$-SUMO-$PqsR_{91\text{-}319}$ was eluted with a linear gradient of buffer B (150 mM $Na_2HPO_4$/ $NaH_2PO_4$, 300 mM NaCl, 50-200 mM imidazole pH 8.0). After cleavage of the $His_6$-SUMO tag by SUMO protease and a second nickel affinity chromatography, $PqsR_{91\text{-}319}$ was finally purified by size exclusion chromatography in 20 mM HEPES, 150 mM NaCl, 1 mM DTT, pH 8.0. The protein was concentrated to 33 mg/ml, flash-frozen in liquid nitrogen and stored at - 80°C.

## 4.2 Crystallization conditions and data analysis

**Protein Crystallization, Data Collection and Structure Solution.** Initial crystals of $PqsR_{91\text{-}319}$ in complex with HHQ, fragment **11** or **20** were obtained by co-crystallization in a sitting drop vapor diffusion setup using the commercially available JCSG Core Suites I-IV (Qiagen), Cryos Suite (Qiagen) and Index HT screen (Hampton Research) at 20°C. Afterwards, initial screening hits were optimized in 96-well plates using a Formulator liquid handling station (Formulatrix). Crystallization drops consisted of 0.2 µl protein-ligand solution and 0.2 µl mother liquor and were prepared with a Honeybee 961 robot (Digilab). The morphology of

$PqsR_{91-319}$-HHQ crystals was improved with the Additive Screen (Hampton Research) by the addition of 0.1 M CsCl. Protein/ligand concentrations and final crystallization conditions that yielded diffraction quality crystals are described in Table S2 for each ligand. The crystals were flash-frozen in liquid nitrogen, and X-ray diffraction data were collected at 100 K at beamlines BL14.1/BL14.2 of the BESSY II synchrotron and at beamline X06DA of the Swiss Light Source (Table S3). Diffraction data were processed with XDS[11] and AIMLESS[12] from the CCP4 suite.[13] The dataset of $PqsR_{91-319}$ in complex with compound **11** was corrected for anisotropy using the STARANISO server[14] (http://staraniso.globalphasing.org) with default settings. The structures were determined by molecular replacement with PHASER[15] or rigid-body refinement with REFMAC5[16] using the PDB entry 4JVC.[17] Model building and refinement were performed with COOT[18] and with phenix.refine[19] from the PHENIX suite,[20] respectively.

Table S2: Crystallization conditions of $PqsR_{91-319}$ in complex with HHQ/11/20

| Protein - ligand complex | [Protein] (mM) | [Ligand] (mM) | Precipitant | Cryoprotectant |
|---|---|---|---|---|
| $PqsR_{91-319}$ - HHQ | 0.58 | 5 | 0.1 M Tris-HCl (pH 8.5), 50% (v/v) ethylene glycol, 0.2 M $MgCl_2$, 0.1 M CsCl | - |

| | | | | |
|---|---|---|---|---|
| PqsR$_{91-319}$ - **11** | 0.78 | 4 | 0.1 M MES (pH 5.7), 0.1 M $NaH_2PO_4$, 0.1 M $K_2HPO_4$, 2.4 M NaCl | 20% (v/v) glycerol |
| PqsR$_{91-319}$ - **20** | 0.39 | 2 | 0.085 M tri-sodium citrate (pH 5.6), 29.8% (v/v) 2-methyl-2-propanol, 15% (v/v) glycerol | - |

Table S3: Data Collection and Statistics

| | $PqsR_{91-319}$ – HHQ | $PqsR_{91-319}$ – 11 | $PqsR_{91-319}$ – 20 |
|---|---|---|---|
| Data collection statistics | | | |
| Beamline[a] | BL 14.2, BESSY II | X06DA, SLS | BL 14.1, BESSY II |
| Wavelength (Å) | 0.9184 | 1.0000 | 1.0000 |
| Space group | $P6_522$ | $C222_1$ | $P6_522$ |
| Unit cell dimensions | | | |
| a, b, c (Å) | 116.6, 116.6, 115.3 | 109.4, 119.3, 114.4 | 121.2, 121.2, 114.6 |
| α, β, γ (°) | 90.0, 90.0, 120.0 | 90.0, 90.0, 90.0 | 90.0, 90.0, 120.0 |
| Resolution range (Å) | 46.25-3.14 (3.36-3.14) | 46.66-2.54 (2.82-2.55) | 47.72-2.82 (2.97-2.82) |
| Ellipsoidal[a] resolution (Å) (direction)[c] | - | 3.32 (a*)<br>3.15 (b*)<br>2.43 (c*) | - |
| Mosaicity[d] (°) | 0.28 | 0.19 | 0.08 |
| Total No. of measured reflections | 192919 (36515) | 201577 (11221) (ellipsoidal) | 298343 (44364) |

| | | | |
|---|---|---|---|
| unique reflections | 8549 (1501) | 15248 (822) (ellipsoidal) | 12501 (1764) |
| Mean I/σ(I) | 15.9 (2.0) | 22.7 (1.7) (ellipsoidal) | 28.7 (2.0) |
| Multiplicity | 22.6 (24.3) | 13.2 (13.7) (ellipsoidal) | 23.9 (25.1) |
| Completeness (spherical) (%) | 99.8 (99.6) | 61.3 (12.5) | 99.9 (99.8) |
| Completeness (ellipsoidal) (%) | - | 94.4 (79.5) | - |
| $R_{meas}$[e] (%) | 16.4 (220.5) | 7.6 (186.7) | 13.0 (202.3) |
| $R_{p.i.m.}$[f] (%) | 3.5 (44.4) | 2.1 (50.3) | 2.6 (40.1) |
| $CC_{1/2}$[g] | 99.9 (77.0) | 99.9 (57.4) | 100.0 (84.2) |
| Solvent content (%) | 72.0 | 66.1 | 73.9 |
| Monomers/ASU | 1 | 2 | 1 |
| Refinement statistics | | | |
| Resolution range (Å) | 46.25-3.14 (3.59-3.14) | 46.66-2.56 (2.76-2.56) | 47.73-2.82 (3.04-2.82) |
| $R_{work}$ (%) | 23.3 (29.9) | 21.5 (38.4) | 23.1 (31.5) |
| $R_{free}$ (%) | 27.1 (36.6) | 24.4 (35.0) | 26.2 (34.6) |
| No. of non H-atoms | | | |

| | | | |
|---|---|---|---|
| Protein | 1778 | 3497 | 1833 |
| Ligand/Ion | 18 | 40 | 34 |
| Water | 6 | 28 | 26 |
| R.m.s. deviations | | | |
| Bonds (Å) | 0.002 | 0.003 | 0.002 |
| Angles (°) | 0.394 | 0.561 | 0.395 |
| Average B factors ($Å^2$) | | | |
| Protein | 131 | 82 | 91 |
| Ligand | 96 | 70 | 85 |
| Water | 98 | 49 | 77 |
| Ramachandran plot (%) | | | |
| Favored regions | 97.5 | 97.0 | 98.5 |
| Outliers | 0.0 | 0.0 | 0.0 |
| MolProbity score[h] | 0.87 | 1.57 | 0.77 |
| PDB ID | 6Q7U | 6Q7V | 6Q7W |

* Data for $PqsR_{91-319}$ in complex with 11 were submitted to the STARANISO server http://staraniso.globalphasing.org.[14]

Values in parentheses refer to the highest resolution shell.

[a] SLS: Swiss Light Source (Paul Scherrer Institute, Villigen, Switzerland). BESSY II: Berlin Electron Storage Ring Company for Synchrotron Radiation (Helmholtz-Zentrum Berlin, Berlin, Germany).

[b] The statistics are for data that were corrected for anisotropy by STARANISO.[14]

[c] The resolution limits for three directions in reciprocal space (a*, b*, c*) are indicated here. To accomplish this, STARANISO[5] computed an ellipsoid postfitted by least squares to the cutoff surface, removing points where the fit was poor. Note that the cutoff surface is unlikely to be perfectly ellipsoidal, so this is only an estimate.

[d] Values as reported by XDS.[11]

[e] $R_{meas} = \Sigma_{hkl} [N / (N - 1)]^{1/2} \Sigma_i |I_i(hkl) - \langle I(hkl) \rangle| / \Sigma_{hkl} \Sigma_i I_i(hkl)$, where N is the multiplicity, $I_i(hkl)$ is the intensity of the ith measurement of the reflection hkl and $\langle I(hkl) \rangle$ is the mean intensity of multiple observations of the reflection hkl.[21,22]

[f] $R_{p.i.m.} = \Sigma_{hkl} [1 / (N - 1)]^{1/2} \Sigma_i |I_i(hkl) - \langle I(hkl) \rangle| / \Sigma_{hkl} \Sigma_i I_i(hkl)$.[21,23]

[g] $CC_{1/2}$: Correlation coefficient between the intensities of two random half data sets.[24]

[h] Quality criterion that includes the clashscore, the Ramachandran and rotamer statistics for the respective structure reported by the MolProbity server0.[25]

## 5. *E. coli* reporter-gene assay & dose-response curves

**PqsR Reporter gene assay in *E. coli*.** The inverse agonistic/antagonistic activity of potential PqsR ligands was evaluated in a β-galactosidase reporter gene assay in *E. coli* as previously described.[26] In short, test compounds were co-incubated with *E. coli* DH5α cells containing the plasmid pEAL08-2,[27] which expresses PqsR and the β-galactosidase LacZ under the control of the *pqsA* promoter. The inverse agonistic/antagonistic activity of compounds was measured in the presence of 50 nM PQS. After incubation, galactosidase activity was quantified photometrically and expressed as ratio of controls. The given $IC_{50}$ values represent the mean of at least two independent experiments using at least eight different concentrations of test compound with n = 4. Non-linear regression analysis was done using the log (inhibitor) vs response model included in the GrapPad Prism 5.0 software package.

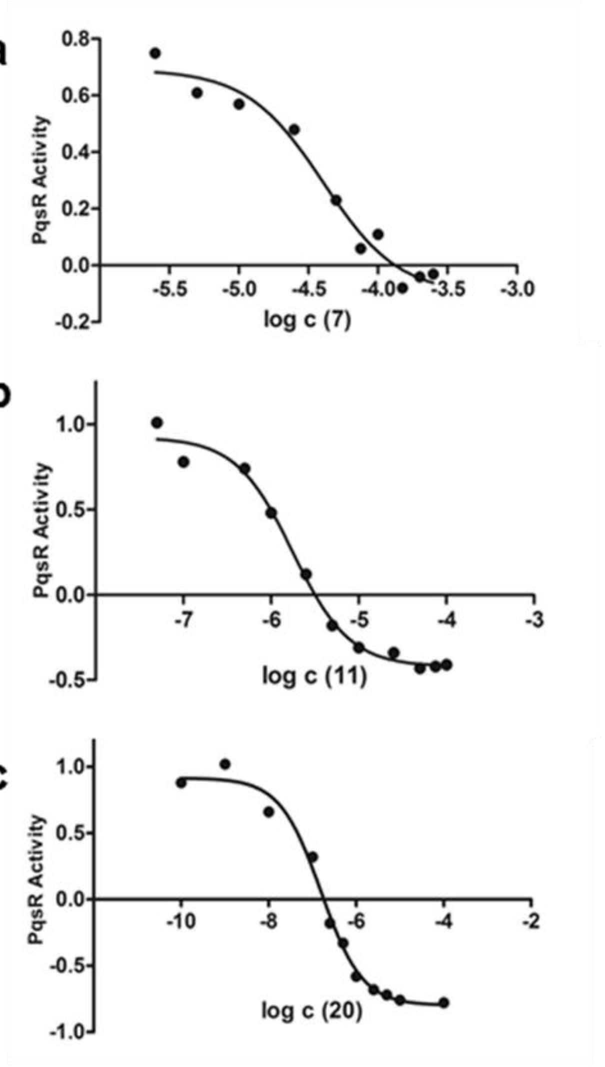


**Figure S3**: Antagonistic activity of different compounds measured in the *E. coli* reporter gene assay. Representative dose-response curves of **a)** compound **7** **b)** compound **11** and **c)** compound **20**. PqsR activity refers to the stimulation of PqsR induced by 50 nM PQS (= 1). Black dotes (•) represent the PqsR activity measured in the presence of a single compound concentration. The continuous black line is the none-linear regression analysis to determine $IC_{50}$ values using a log (inhibitor) vs. response model (Graph Pad Prism 5.04).

# 6. Effects in *P. aeruginosa*

**Pyocyanin Assay.** The inhibition of virulence factor pyocyanin was measured as described previously[26] following a procedure established by Essar et al.[28] Briefly, *Pseudomonas aeruginosa* PA14 cultures were grown in the presence of inhibitors or DMSO as control for 16 h. Pyocyanin was determined photometrically after extraction of the cultures with chloroform and re-extraction with 0.2 M HCl. Protonated red colored pyocyanin was determined by measuring OD520 and normalizing to OD600. None of the compound impaired bacterial viability as observed by the OD600 parameter. Inhibition values were expressed in relation to the DMSO control. Each value is the mean of at least two independent experiments with n=3. The given $IC_{50}$ values represent the mean of at least two experiments using at least five different concentrations. Curve fitting was performed using the log (inhibitor) vs response model with constrained bottom = 0 and top =100 (GraphPad Prism 5.0).

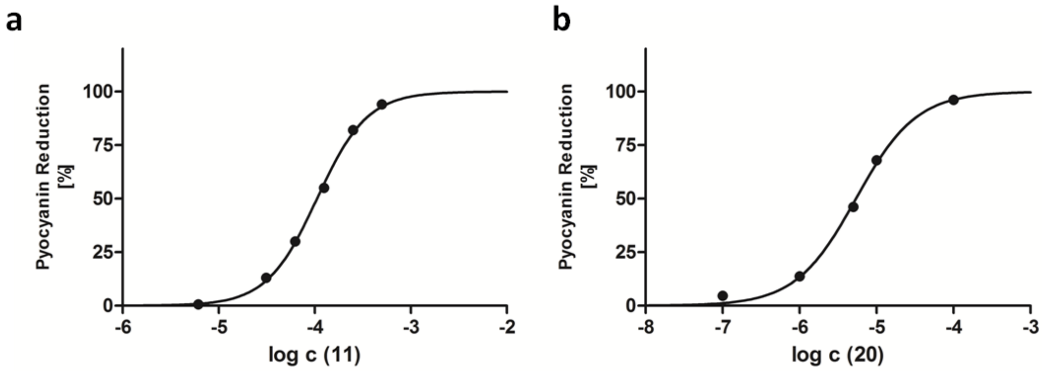


**Figure S4**: Inhibition of virulence factor pyocyanin was evaluated in the clinical isolate PA14. Representative dose-response curves **a)** compound **11** **b)** compound **20**. Black dotes (•) represent the reduction of pyocyanin in presents of a given compound concentration relative to DMSO control (= 0 %). The continuous black line is the none-linear regression analysis to

determine $IC_{50}$ values using a log (inhibitor) vs. response model with constrains (bottom = 0; top =100) (Graph Pad Prism 5.04).

**HHQ and HQNO Quantification.** Extracellular HHQ and HQNO were measured as previously reported[29] following a modified procedure of Lépine et al.:[30] Briefly, *P. aeruginosa* PA14*pqsH* cultures were grown in the presence of DMSO as a control or DMSO solutions of inhibitors for 17 h. HHQ and HQNO were quantified from ethyl acetate extracts using 5,6,7,8-tetradeutero-2-heptyl-4(1H)-quinolone (HHQ-*d4*) as internal standard (IS). UHPLC-MS/MS analysis was performed as described in detail by Storz et al.[31] The following ions were monitored (mother ion [m/z], product ion [m/z], scan time [s], scan width [m/z], collision energy [V], tube lens offset [V]): HHQ: 244, 159, 0.5, 0.01, 30, 106; HQNO: 260, 159, 0.1, 0.01, 25, 88; HHQ-*d4* (IS): 248, 163, 0.1, 0.01, 32, 113. Data acquisition and quantification was performed using Xcalibur software with the use of a calibration curve relative to the area of the IS. Given data represent the mean of at least two independent experiments with n=3 and were expressed as percentage inhibition relative to DMSO control.

# 7. Synthesis and Characterization of Intermediates

## 7.1 Synthesis of intermediates 4a-4g

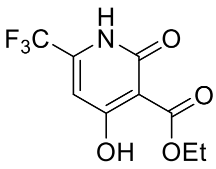

**4a**

**Ethyl-4-hydroxy-2-oxo-6-(trifluoromethyl)-1,2-dihydropyridine-3-carboxylate (4a).** (E)-ethyl 3-amino-4,4,4-trifluorobut-2-enoate (20.1 ml, 137 mmol) and pyridine (13.5 ml, 166 mmol) were dissolved in dry DCM (200 ml). Ethyl-3-chloro-3-oxopropanoate (25.0 g, 166 mmol) was added slowly via syringe while cooling on a water bath. The reaction was stirred at ambient temperature for 4 days. The reaction mixture was diluted with DCM and quenched by addition of water. The aqueous layer was acidified by 2 M HCl. The organic layer was washed with $NaHCO_3$ saturated solution. Both aqueous layers were re-extracted with DCM. The combined organics were washed with brine, dried over MgSO4, filtered and concentrated to yield a brown oil (41 g). Crude product was used in the next step.

The crude (28.2 g, 100 mmol) was dissolved in ethanol (100 ml). Potassium t-butoxide (22.35 g, 199 mmol) was added portionwise while keeping the temperature below 40°C. Afterwards the mixture was heated to 70°C for 2 h and stirred over night at RT. The reaction mixture was concentrated to give a brown solid. Saturated aqueous citric acid solution was added and the suspension stirred for 20 min. The suspension was filtered and the cake washed intensively with water. The resulting solid was dried at vacuum and 50°C to yield ethyl 4-hydroxy-2-oxo-6-(trifluoromethyl)-1,2-dihydropyridine-3-carboxylate as a white solid (12.6 g, 53.1 mmol, 53% yield). $^{1}$H NMR (500 MHz, DMSO-$d_6$) δ ppm 1.25 (t, *J*=7.1 Hz, 3 H), 4.25 (q, *J*=6.9 Hz, 2 H), 6.80 (s, 1 H), 12.07 (br. s., 2 H); $^{13}$C NMR (126 MHz, DMSO-$d_6$) δ ppm 13.94, 60.94, 101.97 (q, *J*=2.7 Hz, 1 C), 105.88, 120.83 (q, *J*=274.9 Hz, 1 C), 162.25, 164.53, 164.56 ( 1C missing). In agreement with the literature.

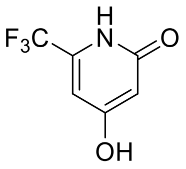

**4b**

**4-hydroxy-6-(trifluoromethyl)pyridin-2(1H)-one (4c)** Ethyl-4-hydroxy-2-oxo-6-(trifluoromethyl)-1,2-dihydropyridine-3-carboxylate (24.9 g, 102.5 mmol) was added portionwise to a stirred solution of 6 M HCl (250 ml) at RT. The suspension was refluxed for 16 h. After cooling cooled to RT, the pH of the reaction mixture was adjusted to 4-6 using 10% aqueous ammonia solution. The suspension was filtered. The resulting cake was slurried in water and filtered again. The solid was dried at high vacuum to give 4-hydroxy-6-(trifluoromethyl)pyridin-2(1H)-one (16.97 g, 92.5 mmol, 90 % yield) as white solid. $^{1}$H NMR (500 MHz, DMSO-$d_6$) δ ppm 6.02 (s, 1 H), 6.59 (s, 1 H); $^{13}$C NMR (126 MHz, DMSO-$d_6$) δ ppm 97.88, 103.17, 121.55 (q, $J$=274.9 Hz, 1 C), 144.23 (q, $J$=33.0 Hz, 1 C), 165.88, 169.20. In agreement with the literatur (see reference)

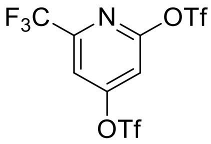

**4c**

**6-(trifluoromethyl)pyridine-2,4-diyl-bis(trifluoromethanesulfonate (4d)** 4-hydroxy-6-(trifluoromethyl)pyridin-2(1H)-one (15.00 g, 84 mmol) was suspended in Acetonitrile (300 ml). Pyridine (14.9 ml, 184 mmol) was added while cooling the mixture on an ice bath. Triflic anhydride (31.1 ml, 184 mmol) was added while keeping the temperature below 10°C. An additional amount of triflic anhydride (10 ml, 59.2 mmol) was added and the reaction stirred over night. The mixture was poured into saturated $Na_2CO_3$ solution and extracted with EA. Combined organic extracts were washed with brine, dried ($MgSO_4$), filtered and concentrated. The crude was purified by coloumn chromatography using PE:EA 95:5 to give 6-(trifluoromethyl)pyridine-2,4-diyl bis(trifluoromethanesulfonate) (12 g, 27.1 mmol, 32.3 % yield) a pale yellow solid $^{1}$H NMR (500 MHz, CHLOROFORM-$d$) δ ppm 7.37 (d, J=1.9 Hz, 1 H), 7.70 (d, J=1.9 Hz, 1 H); $^{13}$C NMR (126 MHz, CHLOROFORM-$d$) δ ppm 111.46, 114.25

(d, J=2.7 Hz, 1 C), 118.53 (q, J=320.8 Hz, 1 C), 118.55 (q, J=320.8 Hz, 1 C), 119.44 (q, J=275.8 Hz, 1 C), 149.70 (q, J=38.5 Hz, 1 C), 156.58, 159.31; MS (APCI-): *m/z* 460 $(M+OH)^{-}$.

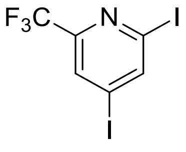


**4d**

**2,4-diiodo-6-(trifluoromethyl)pyridine (4d):** 6-(trifluoromethyl)pyridine-2,4-diyl bis(trifluoromethanesulfonate) (5.00 g, 11.28 mmol) was dissolved in dry acetonitrile (70 ml). KI (11.24 g, 67.7 mmol) was added. Triflic acid (1.50 ml, 16.9 mmol) was added slowly keeping the temperature below 30°C and the reaction was stirred at RT for 24 h under a nitrogen atmosphere. The mixture was quenched by addition of water and $NaS_2SO_3$ 10% (m/V) solution was added dropwise untill the brown color changed to light yellow. ACN was evaporated under reduced pressure and the resulting suspension filtered. The cake was washed intensively with water and dried at high vacuum to give 2,4-diiodo-6-(trifluoromethyl)pyridine (3.6 g, 9.03 mmol, 80 % yield) as pale yellow solid $^{1}$H NMR (500 MHz, CHLOROFORM-d) δ ppm 7.98 (s, 1 H), 8.34 (s, 1 H); 13C NMR (126 MHz, CHLOROFORM-d) δ ppm 106.43, 118.49 (q, J=273.1 Hz, 1 C), 117.69, 129.06 (d, J=2.7 Hz, 1 C), 145.83, 149.13 (q, J=35.7 Hz, 1 C); MS (ESI+): *m/z* 400 $(M+H)^{+}$.

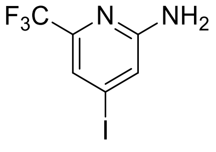


**4e**

**4-iodo-6-(trifluoromethyl)pyridin-2-amine (4e)**. A crimp vial was charged with 2,4-diiodo-6-(trifluoromethyl)pyridine (1.00 g, 2.51 mmol) and DMSO (10 ml). The solution was purged with argon and stirred under an argon atmosphere. 1,10-phenanthroline (0.059 g, 0.251 mmol) and copper(I)oxide (0.018 g, 0.125 mmol) were dissolved in 1 ml DMSO (catalyst solution); this mixture was stirred 5 min at RT under an argon atmosphere. The catalyst solution was transferred to the reaction mixture while cooling on an ice-bath. $NH_{3(aq)}$ (0.35 ml, 2.69 mmol) was slowly added and the reaction stirred at RT for 5 h. An additional amount of $NH_{3(aq)}$

(0.35 ml, 2.69 mmol) was added. DMSO was evaporated under high vacuum at 40°C. The crude was quenched by addition of water and extracted with EA (3 times). The combined organics were washed with $NH_4Cl$ saturated solution and brine, dried over $MgSO_4$, filtered and concentrated. The crude product was purified by chromatography on silica gel (PE:EA 8:2) to give 4-iodo-6-(trifluoromethyl)pyridin-2-amine (390 mg, 1.35 mmol, 54% yield) as pale yellow solid. $^1$H NMR (500 MHz, CHLOROFORM-*d*) δ ppm 4.93 (br. s., 2 H), 7.07 (d, *J*=0.6 Hz, 1 H), 7.32 (d, *J*=0.9 Hz, 1 H); 13C NMR (126 MHz, CHLOROFORM-*d*) δ ppm 105.25, 119.54 (q, J=274.0 Hz, 1 C), 118.11 (q, J=3.7 Hz, 1 C), 119.40, 145.79 (q, J=33.9 Hz, 1 C), 157.60; MS (ESI+): *m/z* 289 $(M+H)^+$.

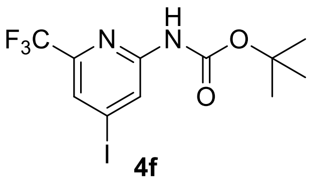


**4f**

**tert-butyl-4-iodo-6-(trifluoromethyl)pyridin-2-yl)-carbamate (4f)** 4-iodo-6-(trifluoromethyl)pyridin-2-amine (200 mg, 0.7 mmol), di-tert-butyl dicarbonate (167 mg, 0.76 mmol), triethylamine (0.10 ml, 0.76 mmol) and DMAP (5 mg, 0.04 mmol) were dissolved in t-butanol (2 ml). The mixture was stirred at 35°C over night. The mixture was quenched with brine and extracted with EA (3 times). The combined organic extracts were washed with brine, dried (MgSO4), filtered and concentrated. The crude was purified by automated flash chromatography (Hex:EA 98:2 to 97:3) to give (4-iodo-6-(trifluoromethyl)pyridin-2-yl)carbamic acid (110 mg, 0,331 mmol, 48 % yield) as a white solid. $^1$H NMR (300 MHz, CHLOROFORM-*d*) δ ppm 1.53 (s, 9 H), 7.32 (br. s., 1 H), 7.67 (d, *J*=1.1 Hz, 1 H), 8.62 (s, 1 H); $^{13}$C NMR (75 MHz, CHLOROFORM-*d*) δ ppm 28.14 (3 c), 82.15, 107.41, 120.24 (d, *J*=273.4 Hz, 1 C), 123.99, 146.53 (d, *J*=33.5 Hz, 1 C), 151.70, 152.09; MS (ESI+) m/z 389 $(M+H)^+$, 333 $(M+H\text{-}tButyl)^+$.

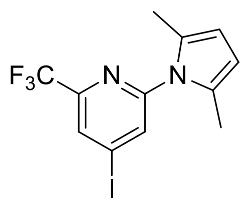

**4g**

**2-(2,5-dimethyl-1H-pyrrol-1-yl)-4-iodo-6-(trifluoromethyl)pyridine (4g)** 4-iodo-6-(trifluoromethyl)pyridin-2-amine (0.50 g, 1.74 mmol) and hexane-2,5-dione (0.24 g, 2.08 mmol) were dissolved in toluol (40 ml). The reaction mixture was heated at reflux using a dean starck trap. The reaction mixture was quenched with $Na_2CO_3$ saturated solution and the aqueous phase was extracted with EA (3 times). The combined organics were washed with brine, dried and concentrated to give a brown oil. The crude product was purified by column chromatography using hexane:EA 90:10 to yield the 2-(2,5-dimethyl-1H-pyrrol-1-yl)- 4-iodo-6-(trifluoromethyl)pyridine (0.55 g, 1.50 mmol, 87 % yield) as brown solid. $^1$H NMR (300 MHz, CHLOROFORM-*d*) δ ppm 2.20 (s, 6 H), 5.93 (s, 2 H), 7.79 (s, 1 H), 7.99 (d, *J*=1.1 Hz, 1 H); MS (ESI+) *m/z* not found.

## 7.2 Synthesis of protected precursors 15a and 16a

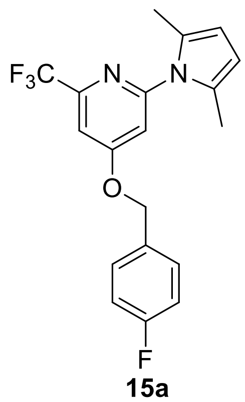

**15a**

**2-(2,5-dimethyl-1H-pyrrol-1-yl)-4-((4-fluorobenzyl)oxy)-6-(trifluoromethyl)pyridine (15a)** (4-fluorophenyl)methanol (517 mg, 4.10 mmol) was slowly added to a suspension of sodium hydride (197 mg, 4.92 mmol) in dry DMF (5 ml). The reaction mixture was heated at 70°C for 10 min and 2-(2,5-dimethyl-1H-pyrrol-1-yl)- 4-iodo-6-(trifluoromethyl)pyridine (300 mg, 0.82 mmol) dissolved in dry DMF (1 ml) was added. The mixture was stirred at 50°C over

night. The reaction mixture was poured into water and EA was added. After stirring for 10 min at RT, the aqueous layer was extracted with EA. The combined organic extracts were washed with $NH_4Cl$ (2 times) and brine (2 times), dried ($MgSO_4$) and concentrated. The crude was purified by column chromatography (Step gradient 99:1 PE:EA to 95:5 PE:EA) to give 2-(2,5-dimethyl-1H-pyrrol-1-yl)-4-((4-fluorobenzyl)oxy)- 6-(trifluoromethyl)- pyridine (80 mg, 0.220 mmol, 26.8 % yield) as yellow oil. $^1$H NMR (300 MHz, CHLOROFORM-*d*) δ ppm 2.15 (s, 6 H), 5.17 (s, 2 H), 5.91 (s, 2 H), 6.87 (d, *J*=2.0 Hz, 1 H), 7.14 (m, 2 H), 7.27 (s, 1 H), 7.35-7.51 (m, 2 H); $^{13}$C NMR (75 MHz, CHLOROFORM-*d*) δ ppm 13.32 (2c), 70.20, 106.92, 107.88, 109.79, 115.99 (d, *J*=21.6 Hz, 2 C), 121.01 (d, *J*=274.9 Hz, 1 C), 128.72, 129.50 (d, *J*=8.2 Hz, 2 C), 130.37 (d, *J*=3.7 Hz, 1 C), 148.88 (q, *J*=34.3 Hz, 1 C), 153.81, 162.94 (d, *J*=250.3 Hz, 1 C), 166.86; MS (ESI+) m/z 365 $(M+H)^+$.

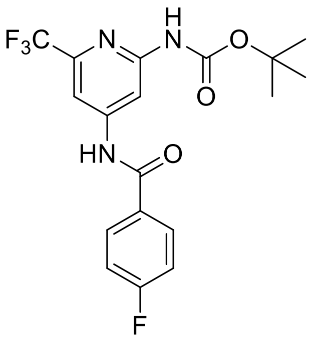


**16a**

**tert-butyl (4-(4-fluorobenzamido)-6-(trifluoromethyl)pyridin-2-yl)carbamate (16a)** A crimp reaction vial was charged with tert-butyl-4-iodo-6-(trifluoromethyl)pyridin-2-yl)-carbamate (75 mg, 0.19 mmol), 4-fluorobenzamide (81 mg, 0.580 mmol) and $Cs_2CO_3$ (189 mg, 0.58 mmol). Dioxane (6 ml) was added and the solution purged with argon. A solution of $Pd_2(dba)_3$ (9 mg, 0.01 mmol) and xantphos (11 mg, 0.02 mmol) was slowly added and the mixture stirred at 50°C for 1 h. An additional amount of Pd2(dba)3 (9 mg, 0.01 mmol) and xantphos (11 mg, 0,02 mmol) were and the temperature was increased to 80°C and stirred at this temperature for 3 h. The reaction mixture was quenched by addition of ether and filtred through cellite. The crude product was adsorbed to silica gel and purified by automated flash chromatography (Hex:EtOAc 100 to 80:20) to give tert-butyl (4-(4-fluorobenzamido)-6-(trifluoromethyl)pyridin-2-yl)carbamate (46 mg, 0,115 mmol, 59,6 % yield) as pale yellow oil.

$^{1}H$ NMR (300 MHz, CHLOROFORM-*d*) δ ppm 1.54 (s, 9 H), 7.15 - 7.26 (m, 2 H), 7.84 - 8.05 (m, 2 H), 8.20 (s, 1 H), 8.27 (s, 1 H); MS (ESI+) *m/z* 400 $(M+H)^+$, 344 $(M+H\text{-t-butyl})^+$.

## 7.3 Synthesis of intermediates 19a, 20a and 21a

**General Procedure for microwave assisted $S_{NAr}$ reactions** 2,4-diiodo-6-(trifluoromethyl)pyridine (0.9 mmol) was dissolved in dry ACN (2 ml). Hunig'sBase (1.8 mmol) and amine (1.0 mmol) were added using a MW vial (CEM®). The mixture was heated for 45 min at 120°C in the microwave. The mixture was poured into $NH_4Cl$ saturated solution and extracted with EA (3 times). Combined organics were washed with brine, dried over $Na_2SO_4$, filtered and concentrated. Regioisomeres and different sideproducts were separated by automated flash chromatography. The 4-substituted regioisomere was identified by 2D NOESY NMR.

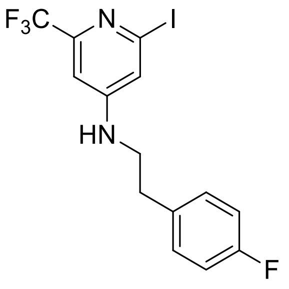


**18a**

**N-(4-fluorophenethyl)-2-iodo-6-(trifluoromethyl)pyridin-4-amine (19a)** was synthesized according to the general procedure from 2,4-diiodo-6-(trifluoromethyl)pyridine (500 mg, 1.25 mmol) and 2-(4-fluorophenyl)ethanamine (192 mg, 1.38 mmol). The crude product was purified by automated flash chromatography using a gradient (PE to PE:EA 75:25) to give N-(4-fluorophenethyl)-2-iodo- 6-(trifluoromethyl)pyridin-4-amine (130 mg, 0.32 mmol, 25% yield) as yellow oil. $^{1}H$ NMR (500 MHz, CHLOROFORM-*d*) d ppm 2.91 (t, J=6.9 Hz, 2 H), 3.44 (td, *J*=6.8, 5.7 Hz, 2 H), 4.42 (br. s., 1 H), 6.73 (d, *J*=1.9 Hz, 1 H), 6.96 (d, *J*=2.2 Hz, 1 H), 7.01 - 7.07 (m, 2 H), 7.14 - 7.19 (m, 2 H); MS (ESI+) *m/z* 411 $(M+H)^+$.

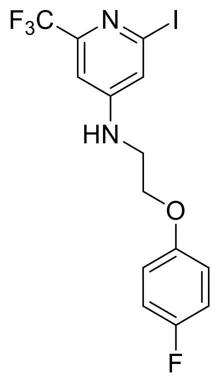

**21a**

**N-(2-(4-fluorophenoxy)ethyl)-2-iodo-6-(trifluoromethyl)pyridin-4-amine (20a)** was synthesized according to the general procedure from (2,4-diiodo-6-(trifluoromethyl)pyridine (350 mg, 0.88 mmol) and 2-(4-fluorophenoxy)ethanamine (150 mg, 0.96 mmol). The crude product was purified by automated flash chromatography using a gradient (PE to PE:EA 55:45) to give N-(2-(4-fluorophenoxy)ethyl)-2-iodo-6-(trifluoromethyl) pyridin-4-amine (60 mg, 0.14 mmol, 16 % yield) as yellow oil. $^1$H NMR (500 MHz, CHLOROFORM-*d*) δ ppm 3.58 (q, *J*=5.4 Hz, 2 H), 4.14 (t, *J*=5.0 Hz, 2 H), 4.88 (br. s., 1 H), 6.81 - 6.90 (m, 3 H), 6.96 - 7.05 (m, 2 H), 7.07 (d, *J*=2.2 Hz, 1 H); MS (ESI+) *m/z* 427 $(M+H)^+$.

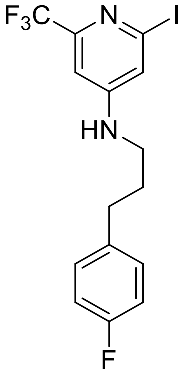

**20a**

**N-(3-(4-fluorophenyl)propyl)-2-iodo-6-(trifluoromethyl)pyridin-4-amine (21a)** was synthesized according to Method B from 2,4-diiodo-6-(trifluoromethyl)pyridine (500 mg, 1.25 mmol) and 3-(4-fluorophenyl)propan-1-amine (211 mg, 1.38 mmol). The crude product was purified by automated flash chromatography using a gradient (PE to PE:EA 75:25) to give N-(3-(4-fluorophenyl)propyl)-2-iodo-6-(trifluoromethyl) pyridin-4-amine (140 mg, 0.33 mmol, 26 % yield) as yellow oil. 1H NMR (500 MHz, CHLOROFORM-*d*) d ppm 1.95 (quin, J=7.4 Hz, 2 H), 2.71 (t, *J*=7.4 Hz, 2 H), 3.17 (td, *J*=7.1, 5.7 Hz, 2 H), 4.40 (br. s., 1 H), 6.70 (d, *J*=2.2

Hz, 1 H), 6.91 (d, $J$=2.2 Hz, 1 H), 6.99 - 7.05 (m, 2 H), 7.11 - 7.17 (m, 2 H); MS (ESI+) *m/z* 425 $(M+H)^{+}$.

## 7.4 Synthesis of intermediates 22a and 22b

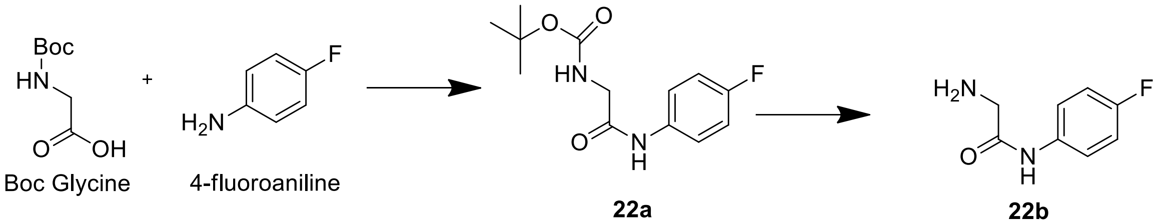


**22a** **22b**

**tert-butyl (2-((4-fluorophenyl)amino)-2-oxoethyl)carbamate (22a)**: BOC Glycine (1.65 g, 9.45 mmol), EDC HCl (3.45 g, 18 mmol) and DMAP (0.110 g, 0.90 mmol) were filled into a three necked flask and suspended in THF (Volume: 20 ml). Et3N (3,76 ml, 27,0 mmol) and 4-fluoroaniline (1.00 g, 9.00 mmol) were added and the white suspension stirred under a nitrogen atmosphere for 8 h at RT. The mixture was poured into 10% citric acid and stirred for 10 min. The white precipitate was filtered off and the resulting cake washed 2 times with water. The white solid was dried at high vacuum over night. $^{1}H$ NMR (300 MHz, CHLOROFORM-*d*) δ ppm 1.49 (s, 9 H), 3.92 (d, $J$=6.1 Hz, 2 H), 5.19 (br. s., 1 H), 6.92 - 7.11 (m, 2 H), 7.35 - 7.55 (m, 2 H), 8.12 (br. s., 1 H); MS (ESI-) *m/z* 267 $(M-H)^{-}$.

**2-amino-N-(4-fluorophenyl)acetamide (22b):** tert-butyl(2-((4-fluorophenyl)amino)-2-oxo- ethyl) carbamate (1.60 g, 5.96 mmol) was supended in DCM (20 ml). TFA (2.5 ml, 32.4 mmol) was added while cooling on an ice bath. The reaction was stirred at RT for 8 h. The mixture was poured into a NaHCO3 saturated solution and extracted with EA (5 times). Combined organics were washed with brine, dried ($MgSO_4$), filtered and concentrated. The product was dried at high vacuum to give 2-amino-N-(4-fluorophenyl)acetamide (0.6 g, 3.57 mmol, 59.8 % yield) as yellow material. $^{1}H$ NMR (500 MHz, DMSO-$d_6$) δ ppm 3.31 (s, 2 H), 7.08 - 7.19 (m, 2 H), 7.59 - 7.70 (m, 2 H); $^{13}C$ NMR (126 MHz, DMSO-$d_6$) δ ppm 44.78, 115.07

(d, *J*=22.9 Hz, 2 C), 120.59 (d, *J*=7.3 Hz, 2 C), 135.01 (d, *J*=2.8 Hz, 1 C), 157.76 (d, *J*=238.3 Hz, 1 C), 171.06; MS (ESI+) *m/z* 169 $(M+H)^{+}$.

## 8. High Resolution Mass Spectroscopy

All measurements were performed on a Dionex Ultimate 3000 RSLC system using a Waters BEH C18, 50 x 2.1 mm, 1.7 µm dp column. Separation of 1 µL sample was achieved by a linear gradient with (A) $H_2O$ + 0.1% FA to (B) ACN + 0.1% FA at a flow rate of 600 µL/min and 45 °C. The gradient was initiated by a 1 min isocratic step at 5% B, followed by an increase to 95% B in 6 min to end up with a 1.5 min step at 95% B before reequilibration with initial conditions. UV spectra were recorded by a DAD in the range from 200 to 600 nm. The LC flow was split to 75 µL/min before entering the maXis 4G hr-ToF mass spectrometer (Bruker Daltonics) using the standard ESI source. Mass spectra were acquired in centroid mode ranging from 50 – 1000 m/z at 2 Hz scan speed.

## 9. Purity of Final Compounds (LC/MS Determination)

Purity control was carried out on two different systems:

SpectraSystems LC system (Thermo Fisher Scientific) consisting of a pump, an autosampler, and a VWD detector. Mass spectrometry was performed on an MSQ electro spray mass spectrometer (Thermo Fisher Scientific). The system was operated by the standard software Xcalibur. An RP-C18 NUCLEODUR 100-5 (125x3 mm) column (Macherey-Nagel GmbH) was used as stationary phase. All solvents were HPLC grade. For purity determination the following methods was used:

Mobil phase, A = water + 0.1% trifluoroacetic acid, B = acetonitrile + 0.1% trifluoroacetic acid; gradient, 0.0-15.0 min, 0-100% B, 15.0-20.0 min, 100% B; flow rate 0.8 mL/min.

LCMS-System (Waters) consisting of a 767 sample Manager, a 2545 binary gradient pump, a 2998 PDA detector and a 3100 electron spray mass spectrometer equipped with a C-18

column (Nucleodur 100-5 C18 ec 150 x 4.6 mm). For purity determination the following methods was used:

Mobil phase, A = water + 0.1% formic acid, B = acetonitrile + 0.1% formic acid; gradient, 0.0-13.0 min, 0-100% B; flow rate 1 mL/min

# 10. UV traces HPLC

Compound 9

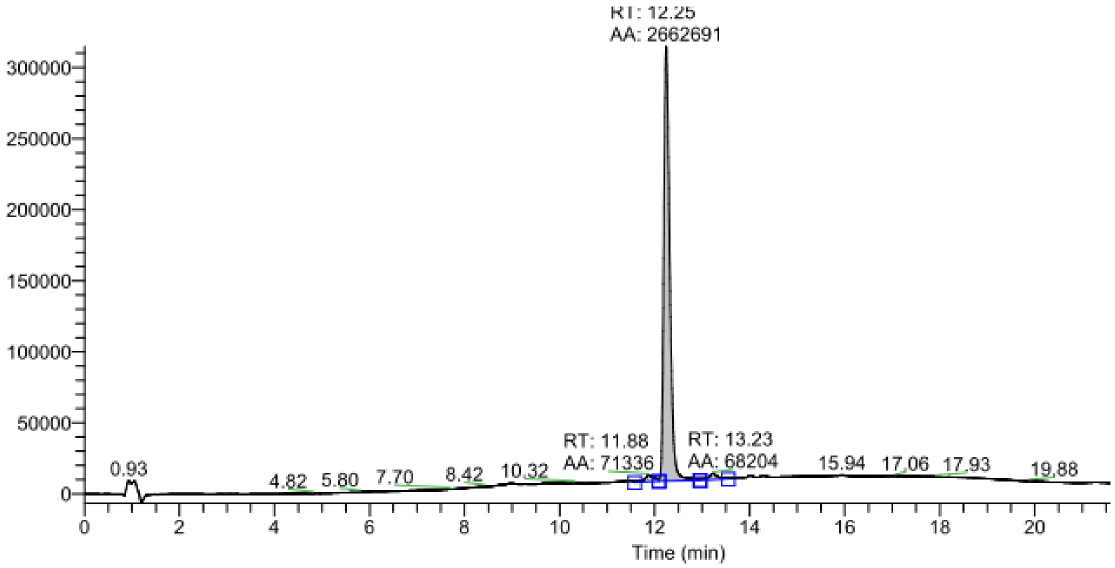


Compound 10

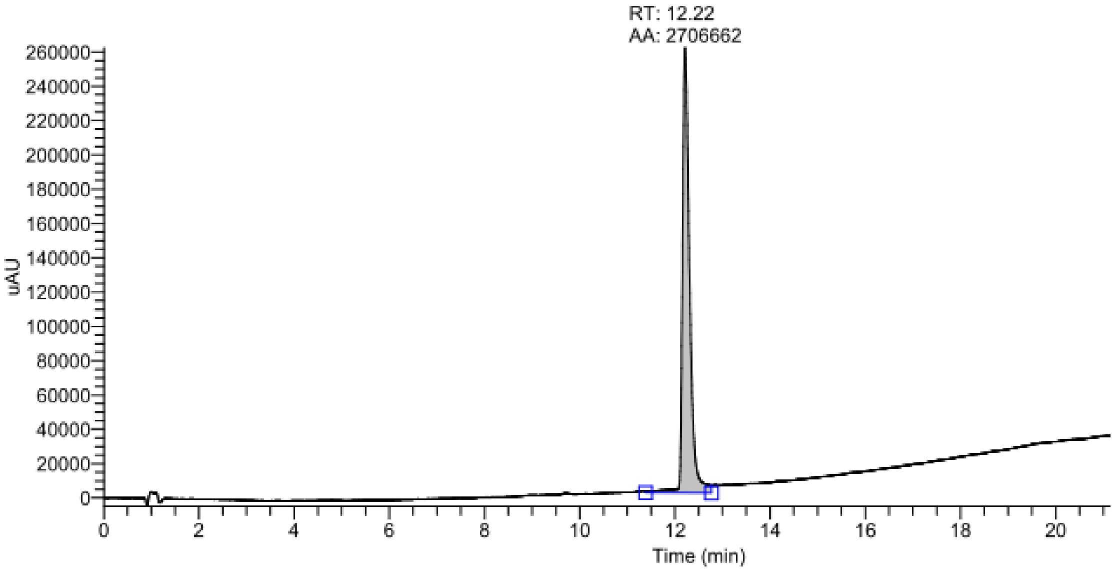

Compound 11

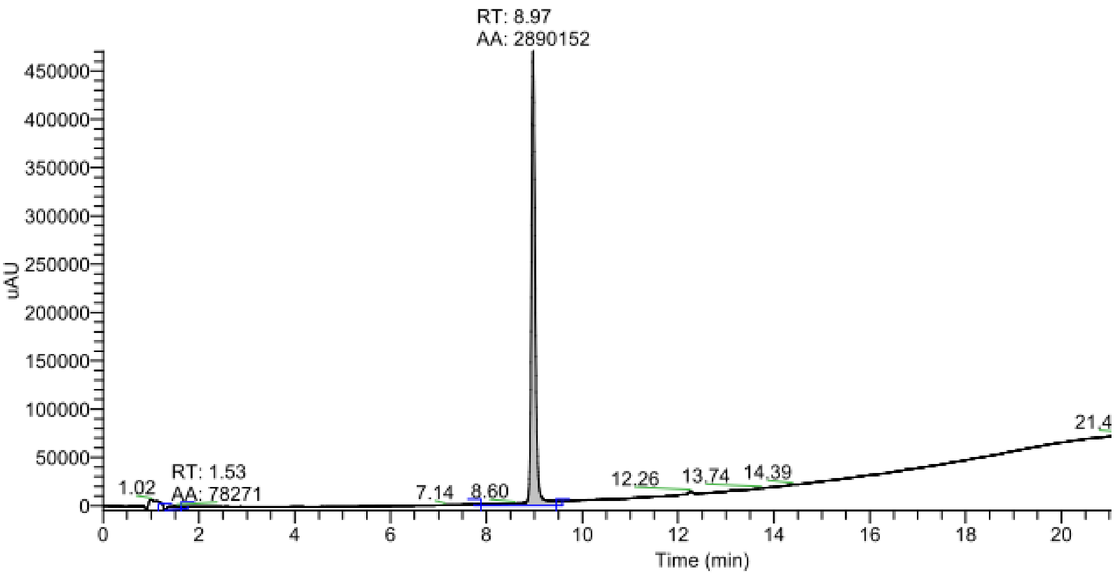


Compound 12

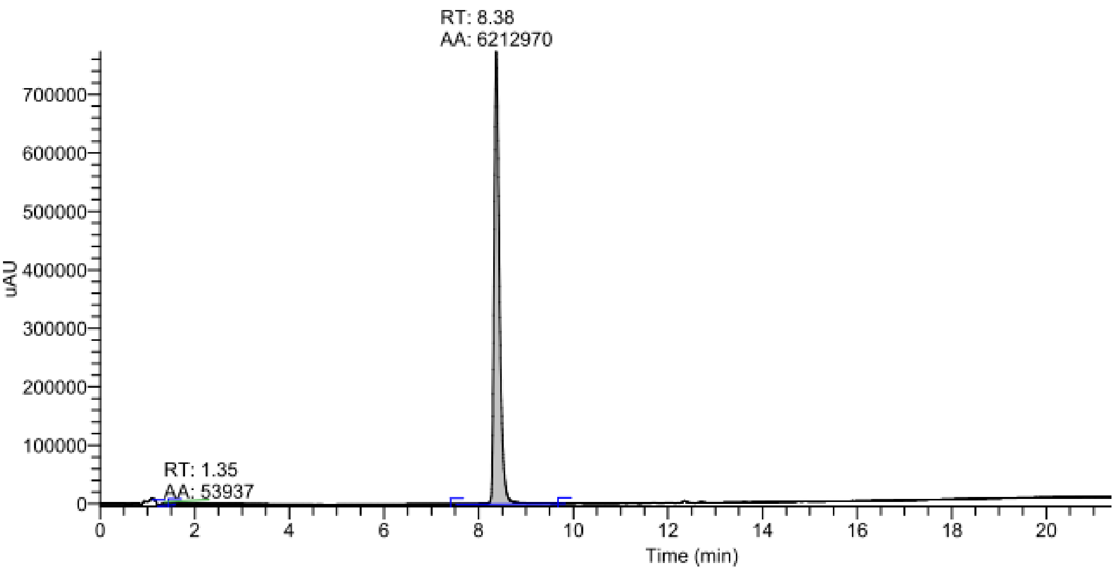

Compound 13

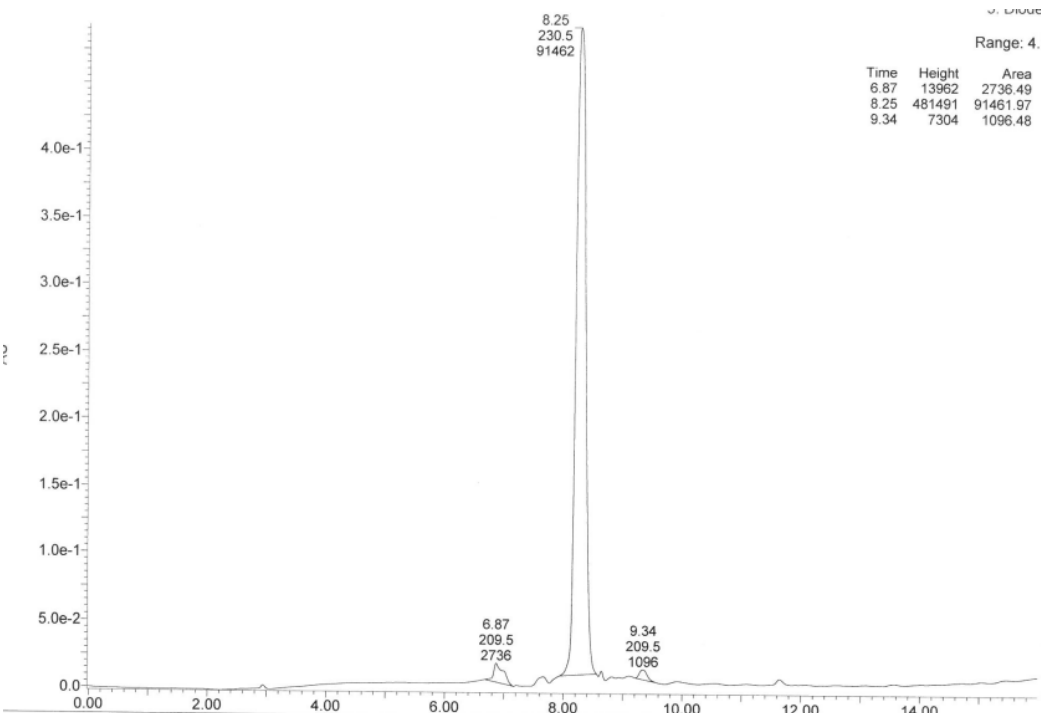


Compound 14

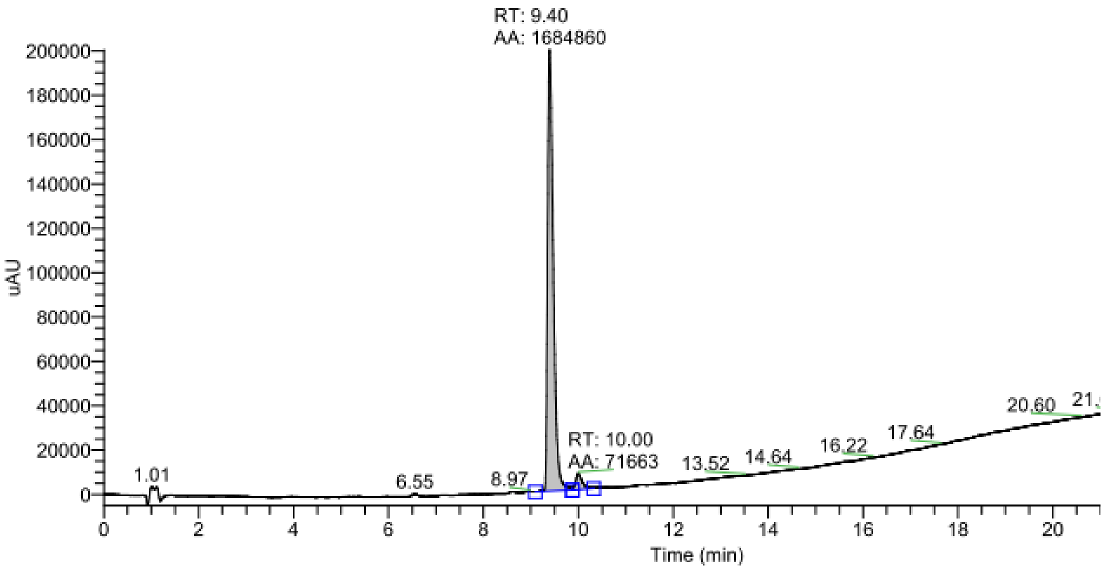
RT: 9.40
AA: 1684860
RT: 10.00
AA: 71663
uAU
Time (min)

Compound 15

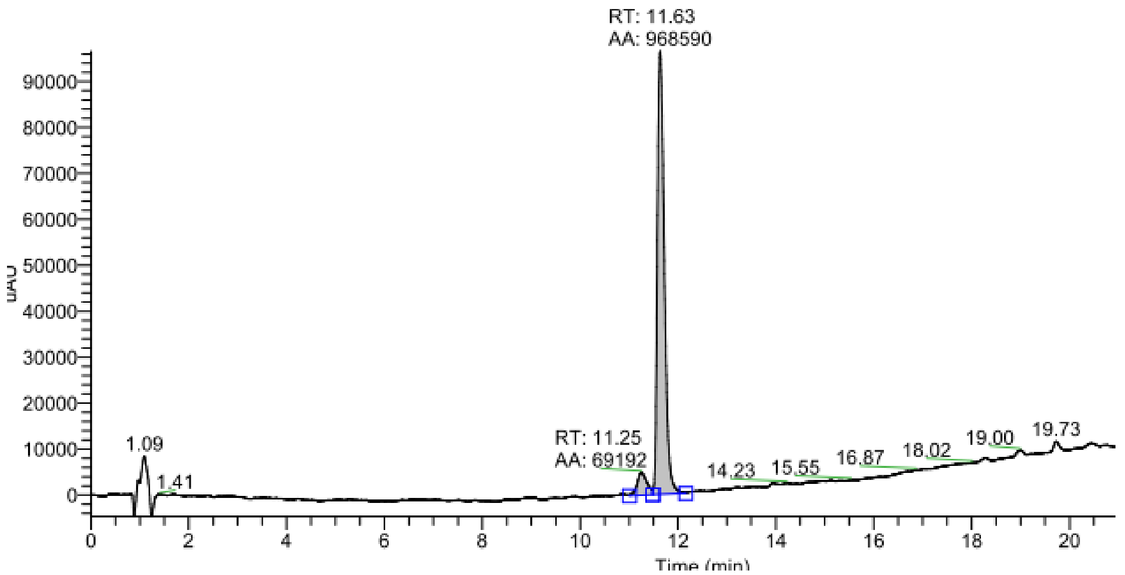
RT: 11.63
AA: 968590
RT: 11.25
AA: 69192

Compound 16

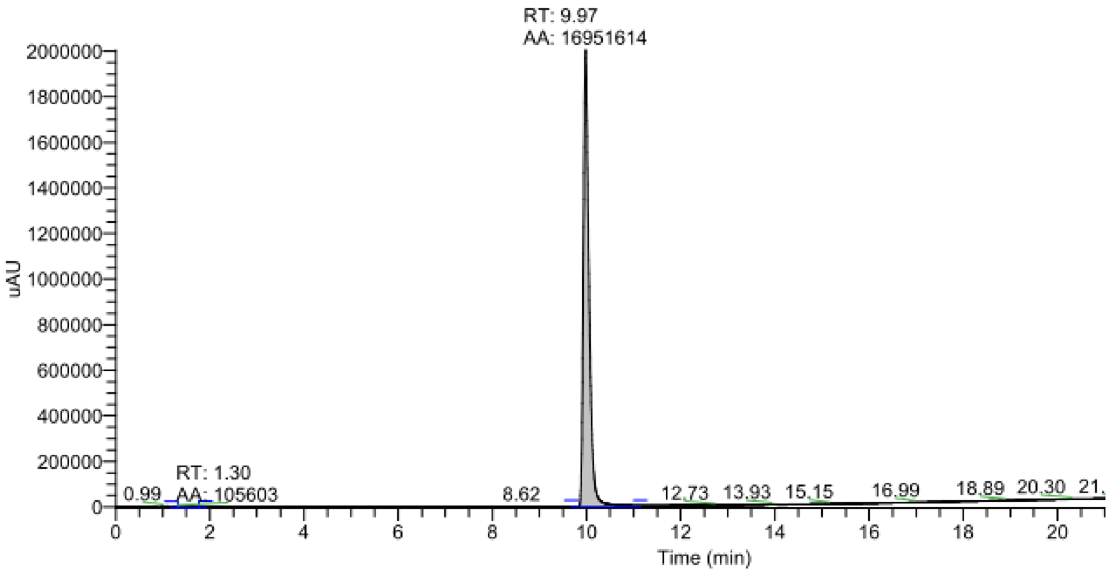


Compound 17

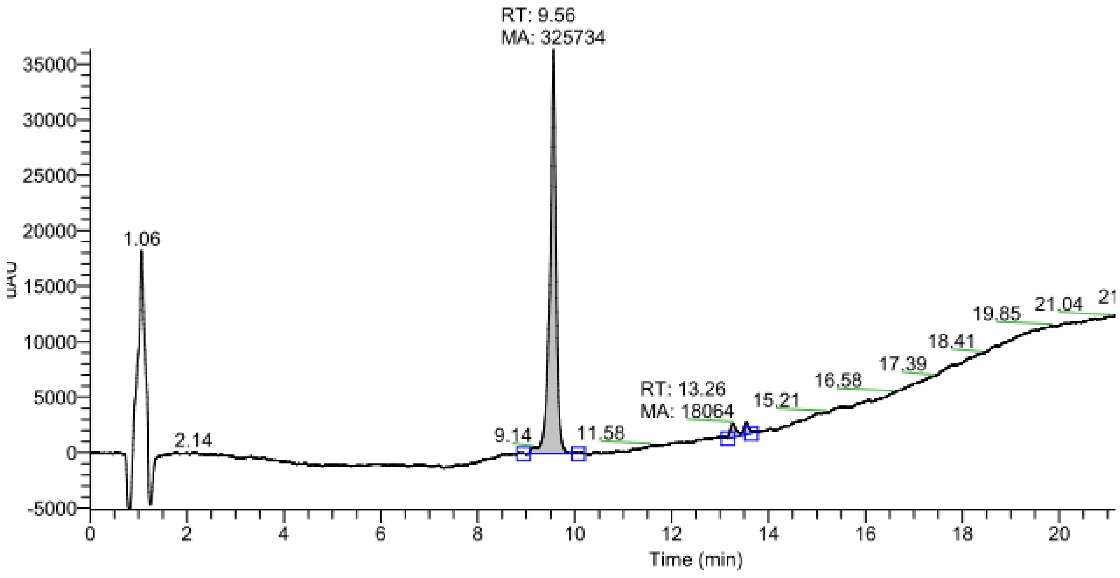


Compound 18

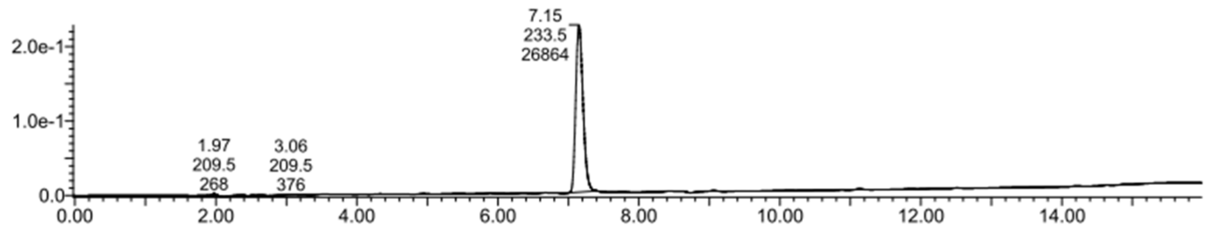


Compound 19

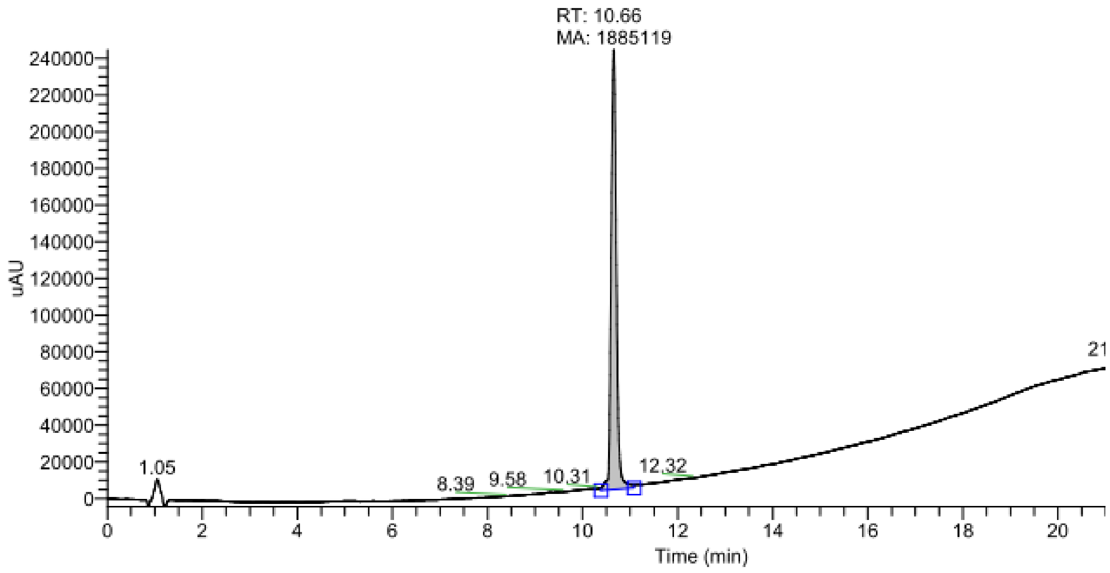

Compound 20

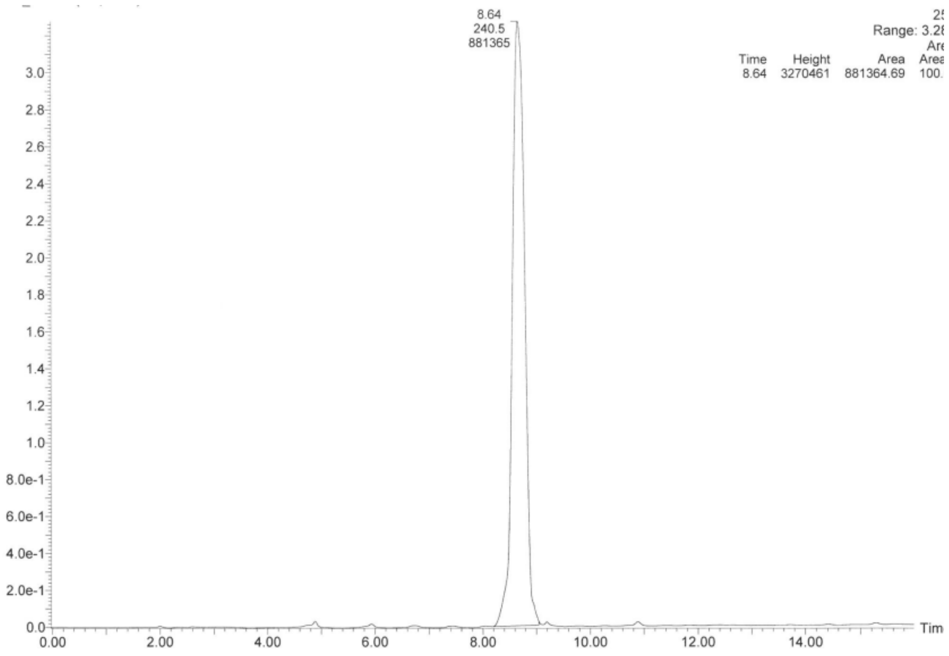


Compound 21

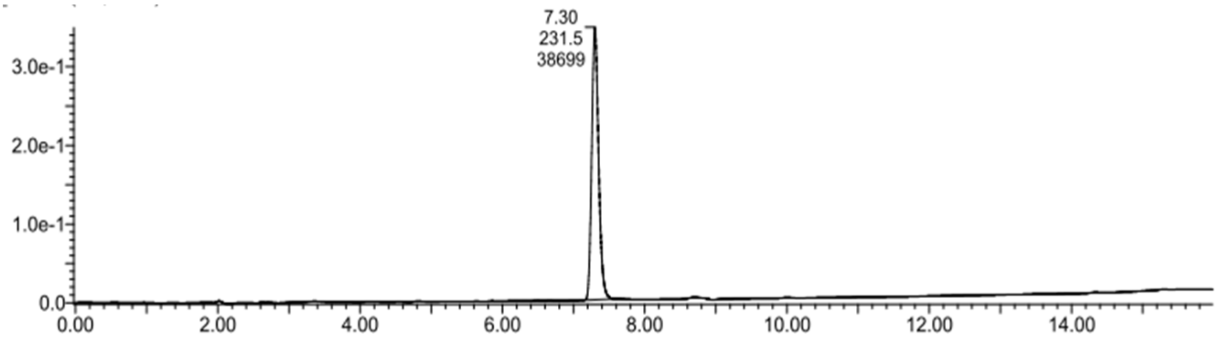


Compound 22

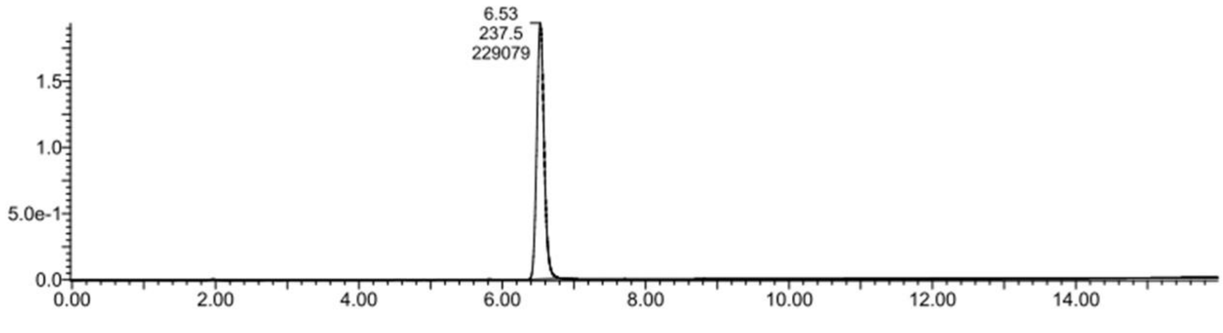

# 11. $^{1}$H NMR spectra

Compound 9

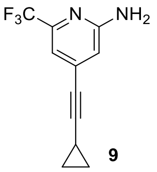


9

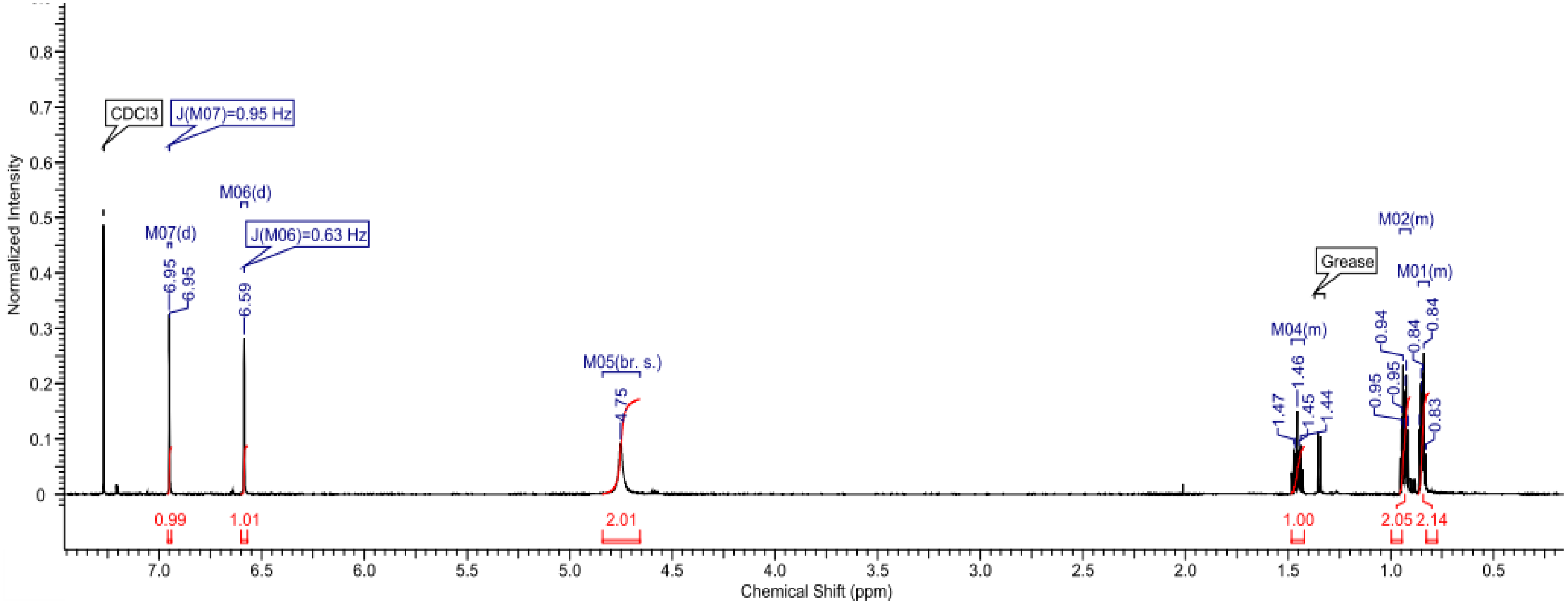

Compound 10

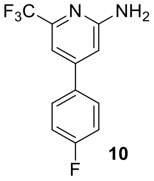


10

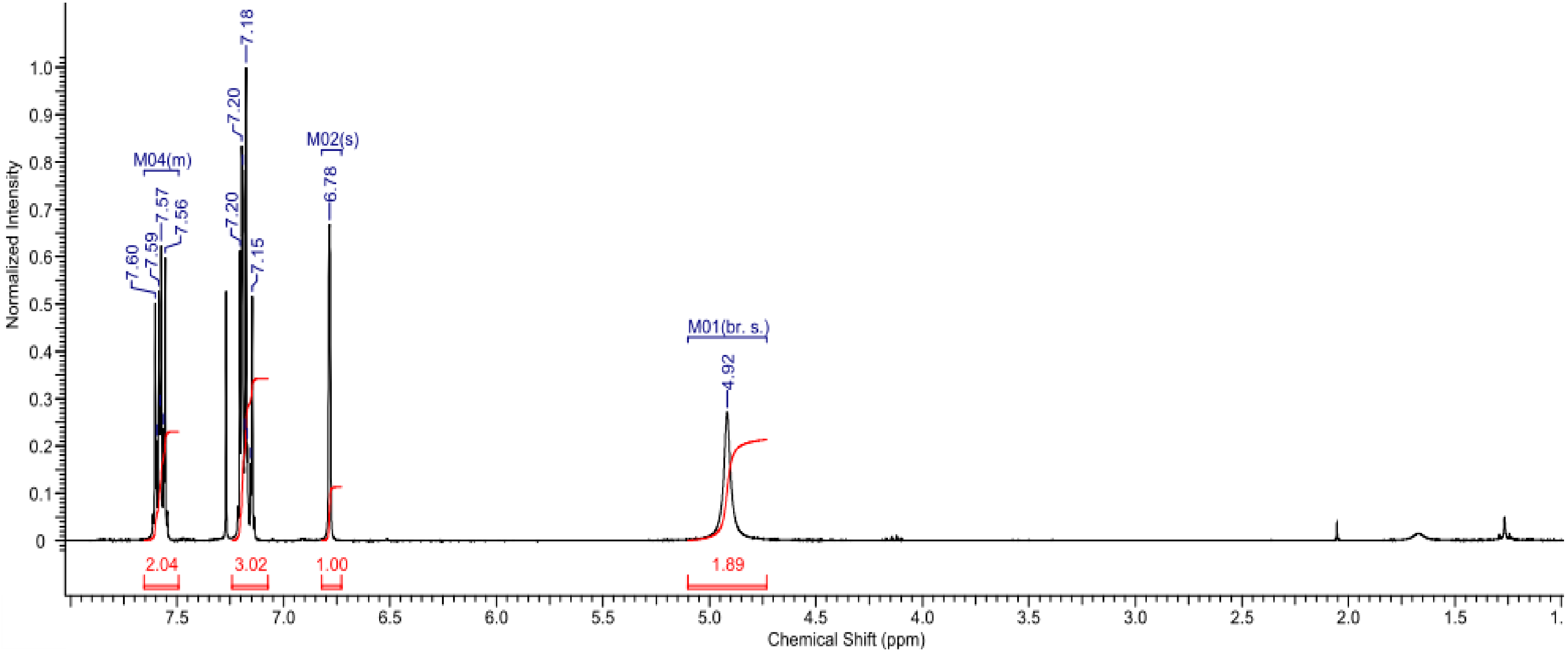

Compound 11

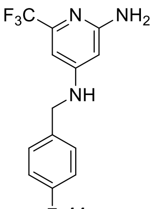
F3C
N
NH2
NH
F
11

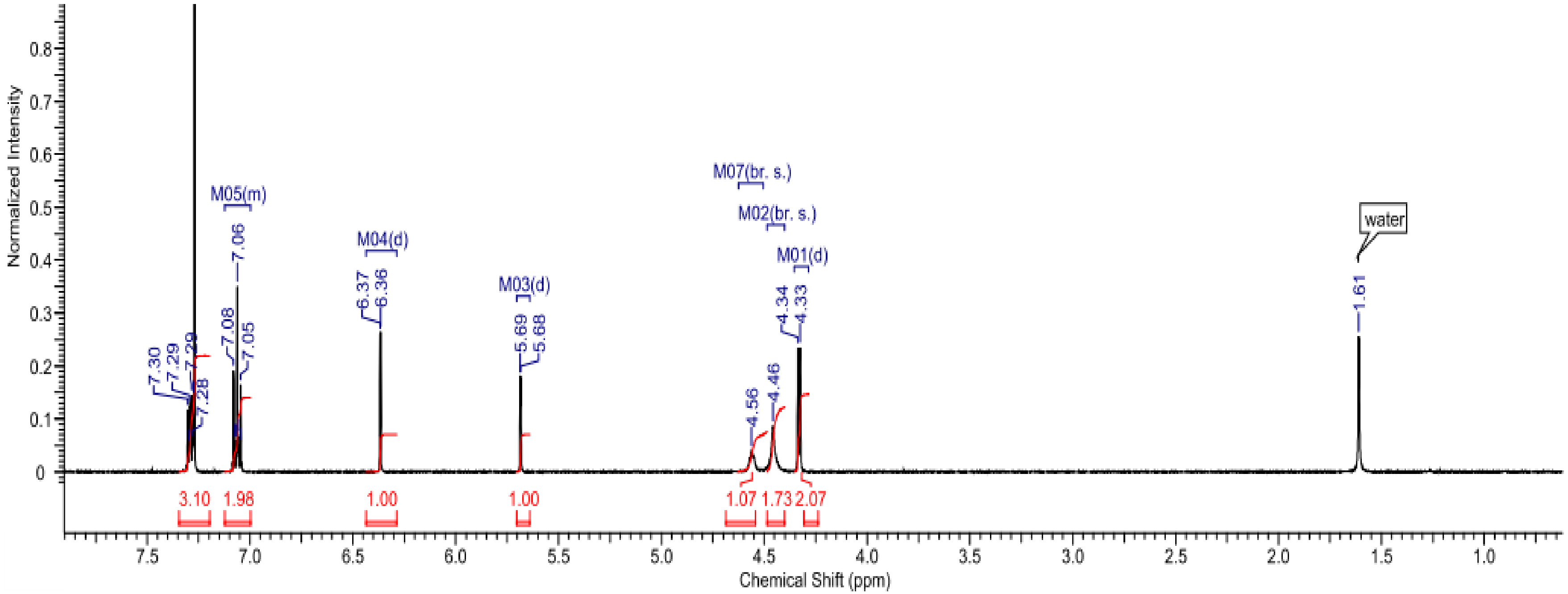
Normalized Intensity
Chemical Shift (ppm)
M05(m)
M04(d)
M03(d)
M07(br. s.)
M02(br. s.)
M01(d)
water
7.30
7.29
7.28
7.08
7.06
7.05
6.37
6.36
5.69
5.68
4.56
4.46
4.34
4.33
1.61
3.10
1.98
1.00
1.00
1.07
1.73
2.07

Compound 12

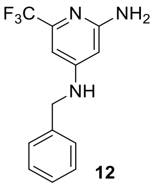

12

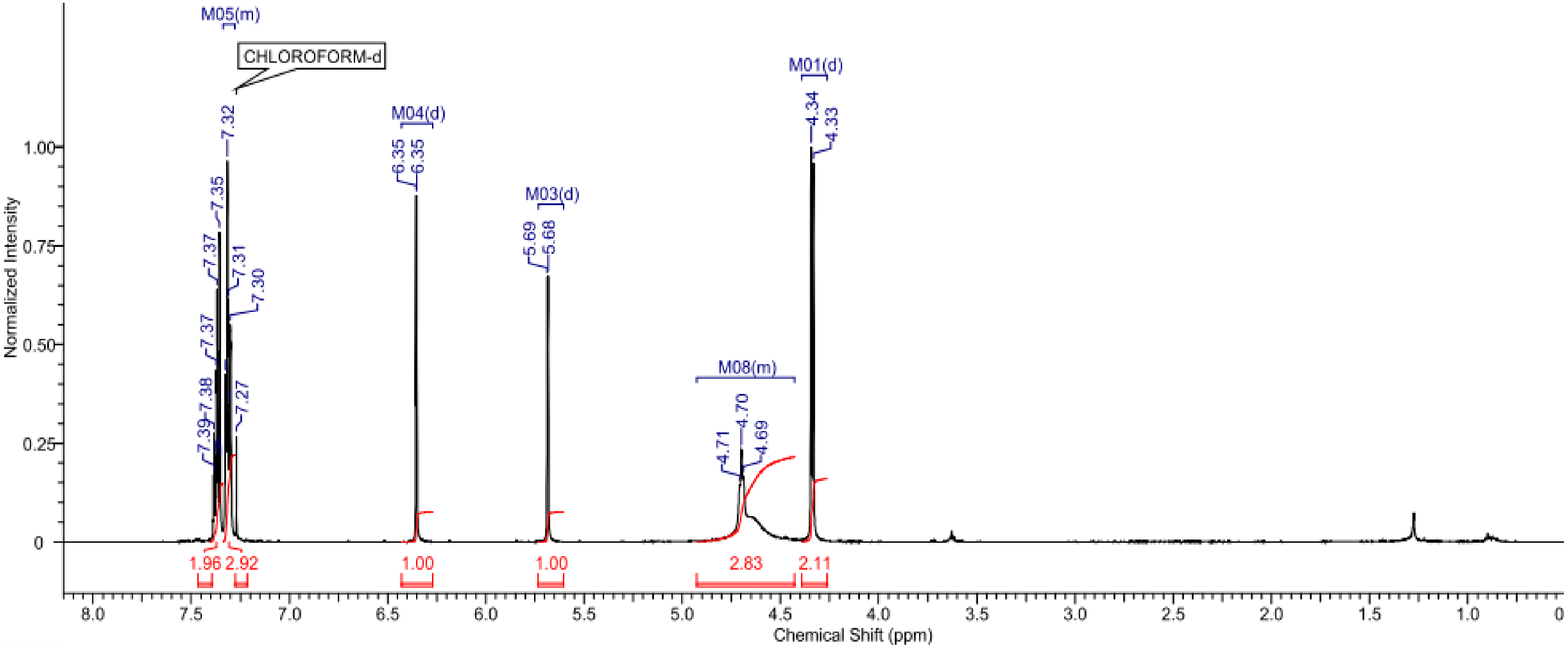

Compound 13

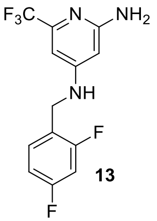


13

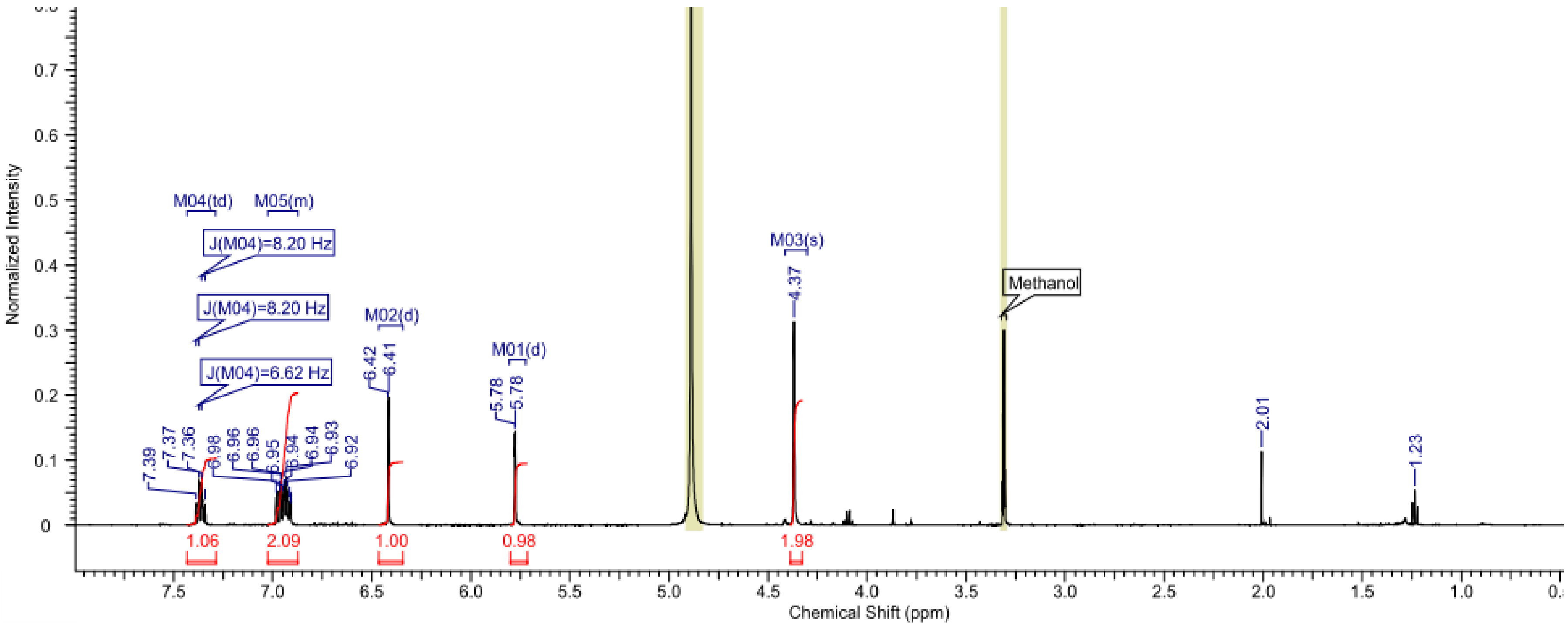

Compound 14

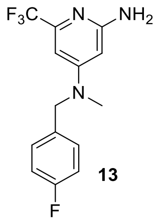

13

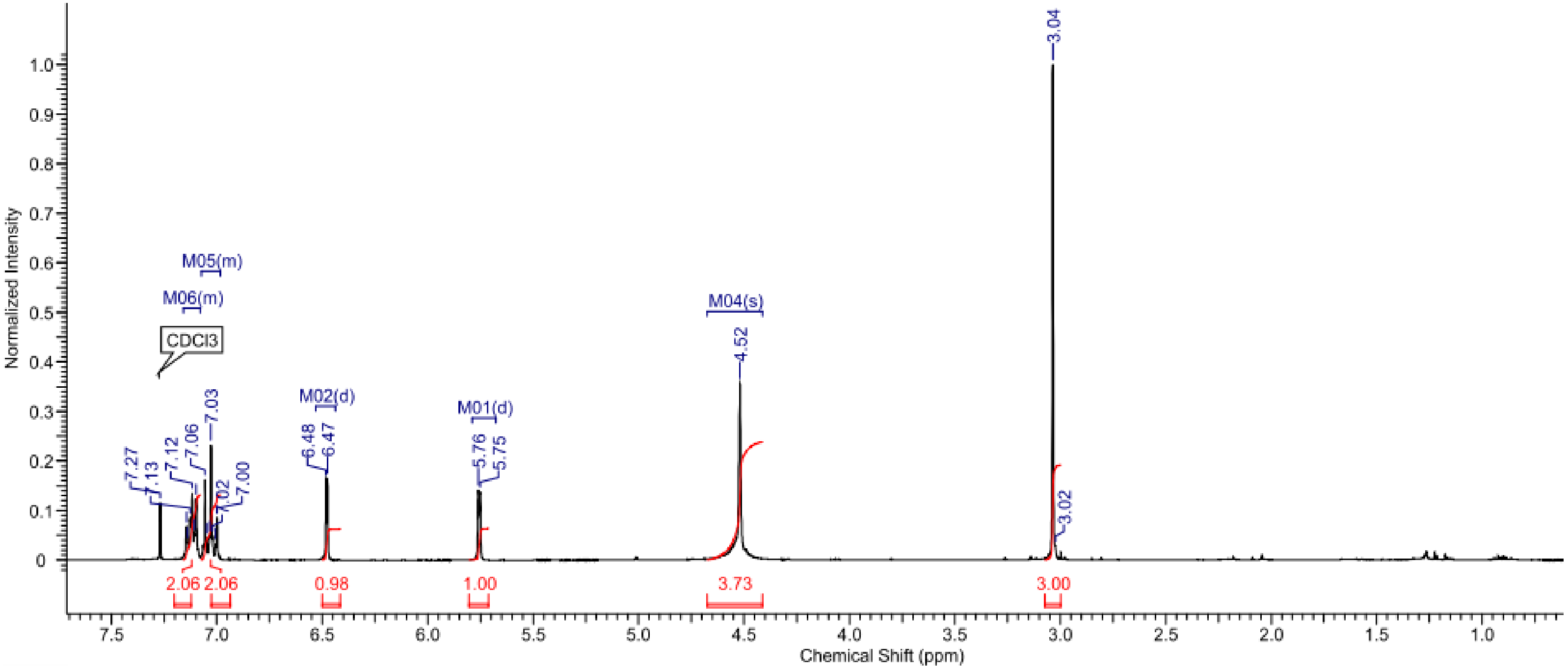

Compound 15

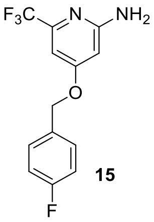


15

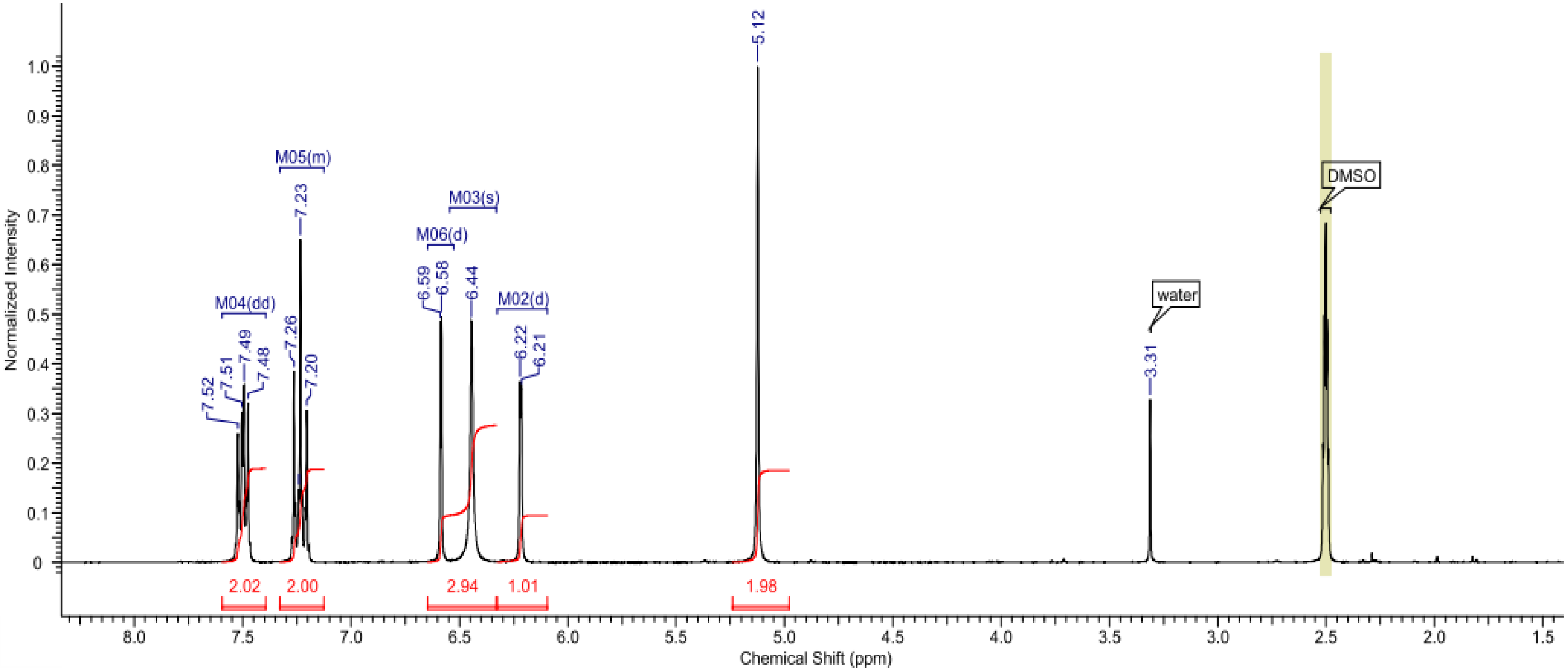

Compound 16

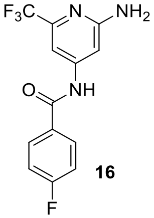


16

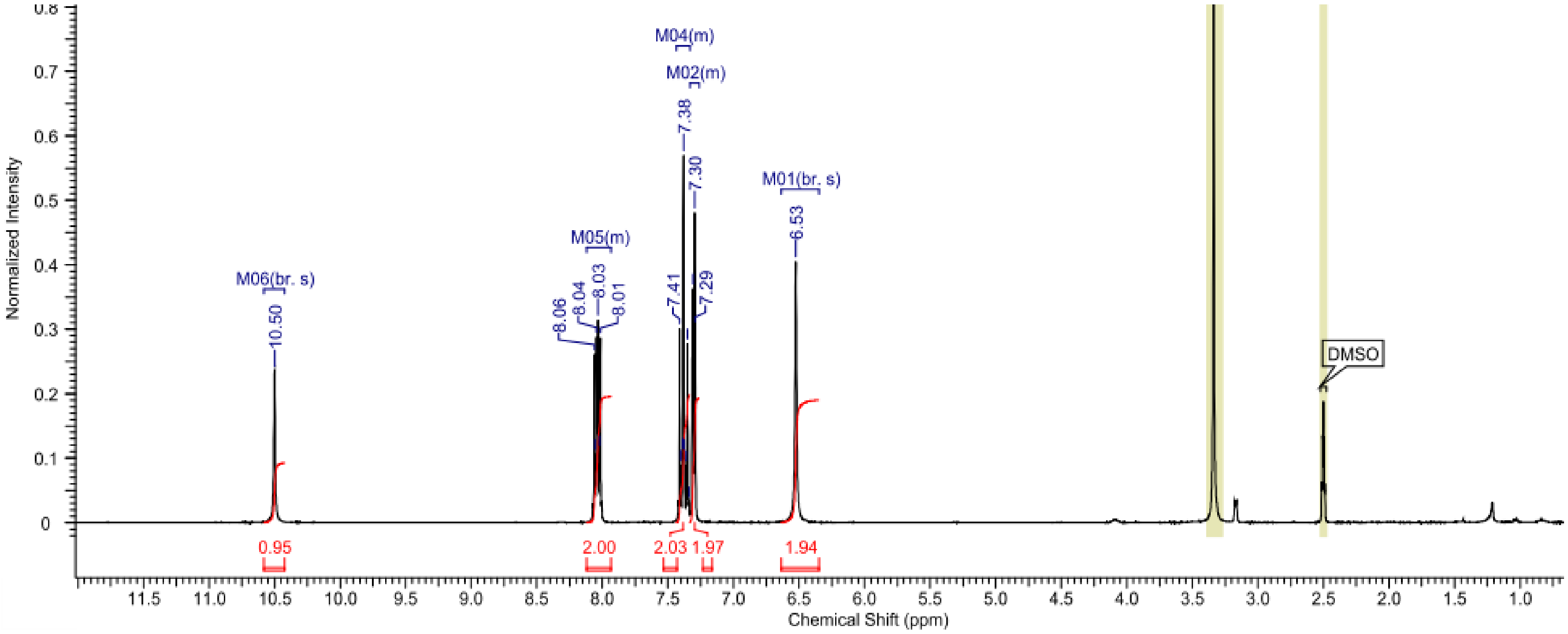

Compound 17

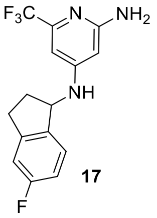


17

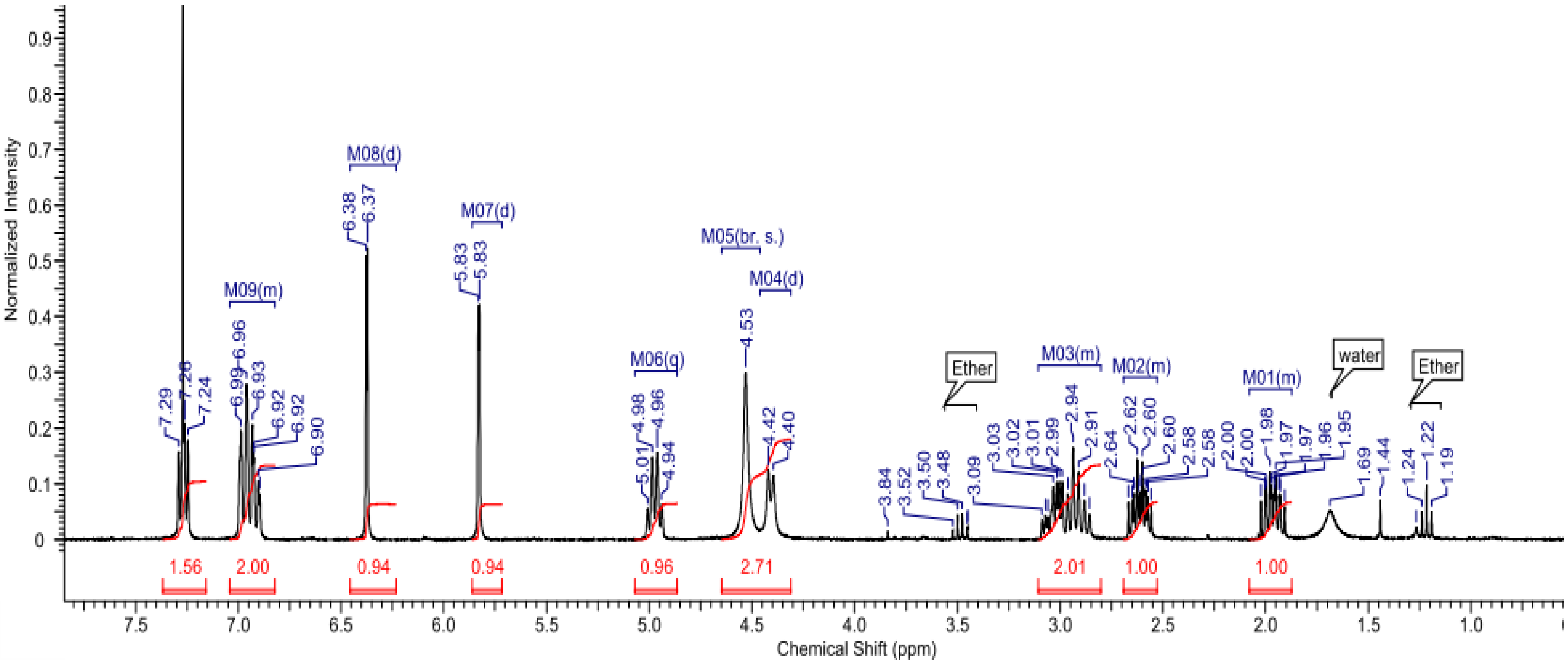

Compound 18

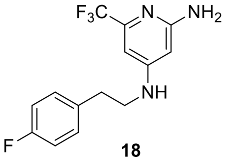

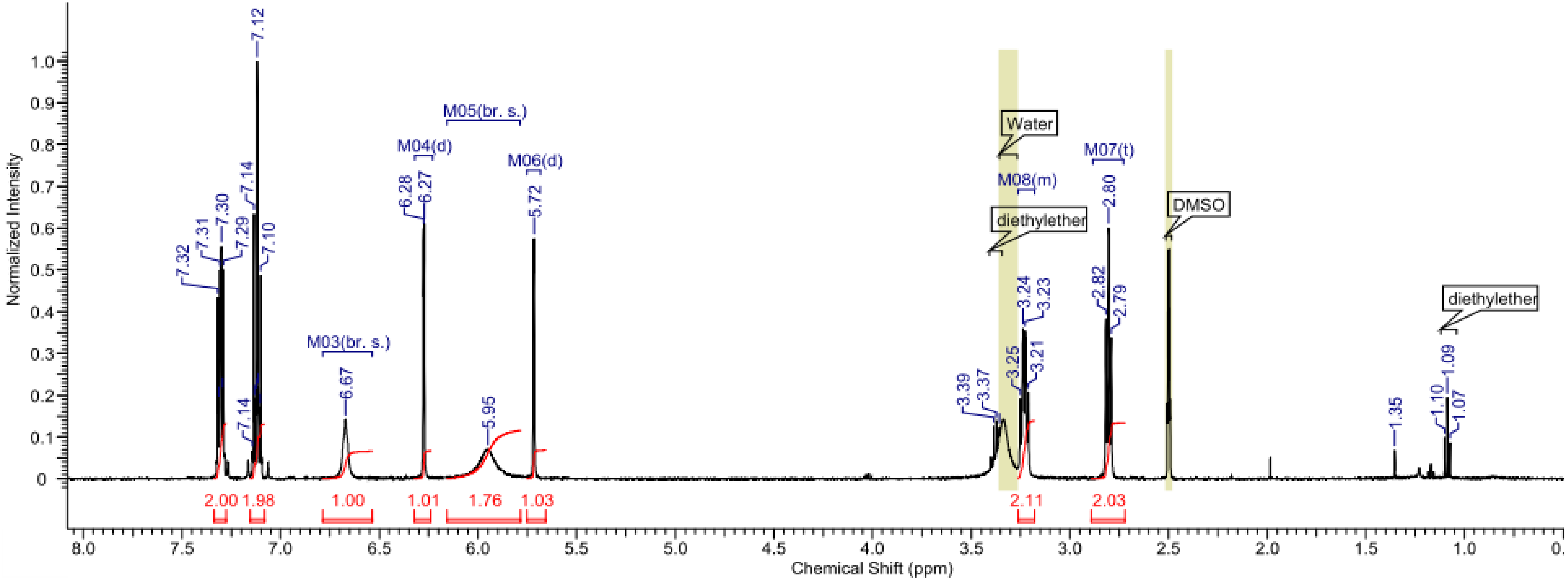

M03(br. s.)
M04(d)
M05(br. s.)
M06(d)
M07(t)
M08(m)
Water
diethylether
DMSO
diethylether
Normalized Intensity
Chemical Shift (ppm)

Compound 19

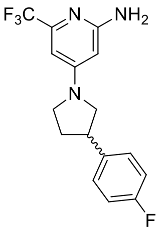


19

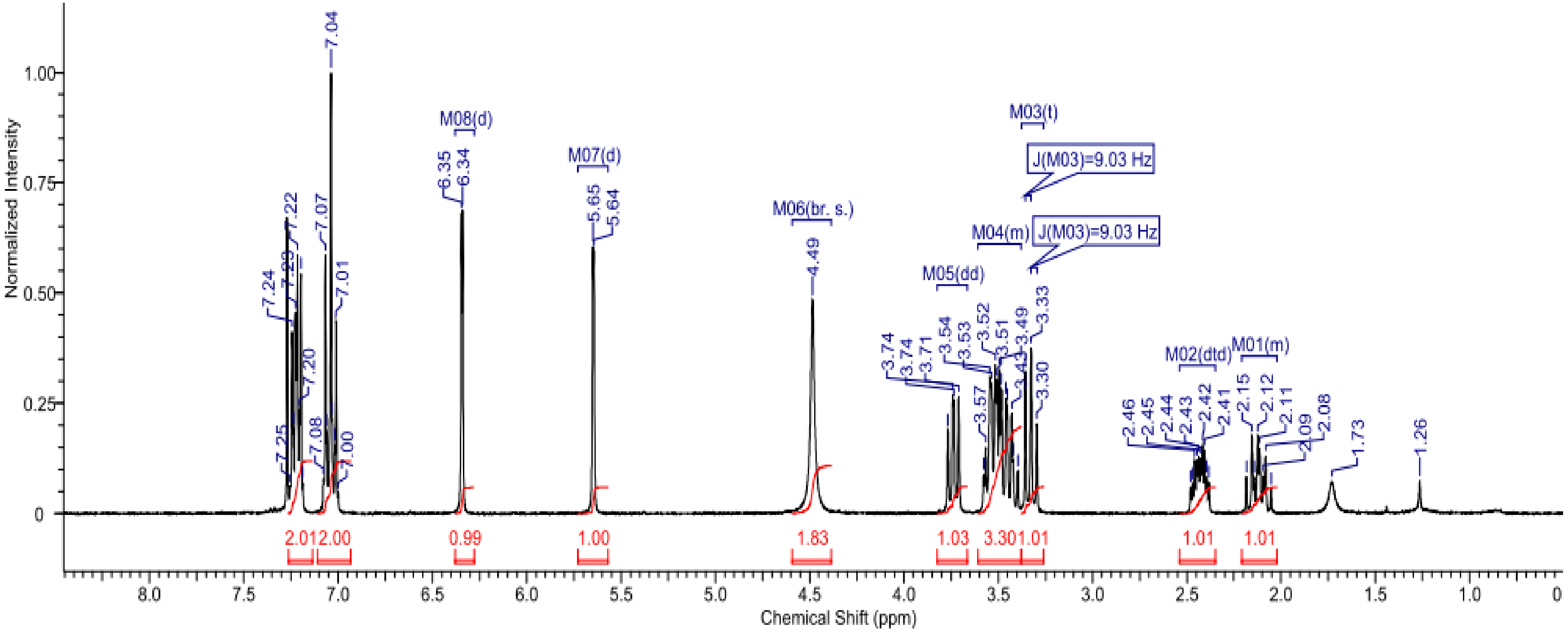

Compound 20

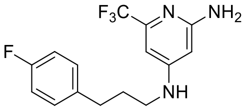


**20**

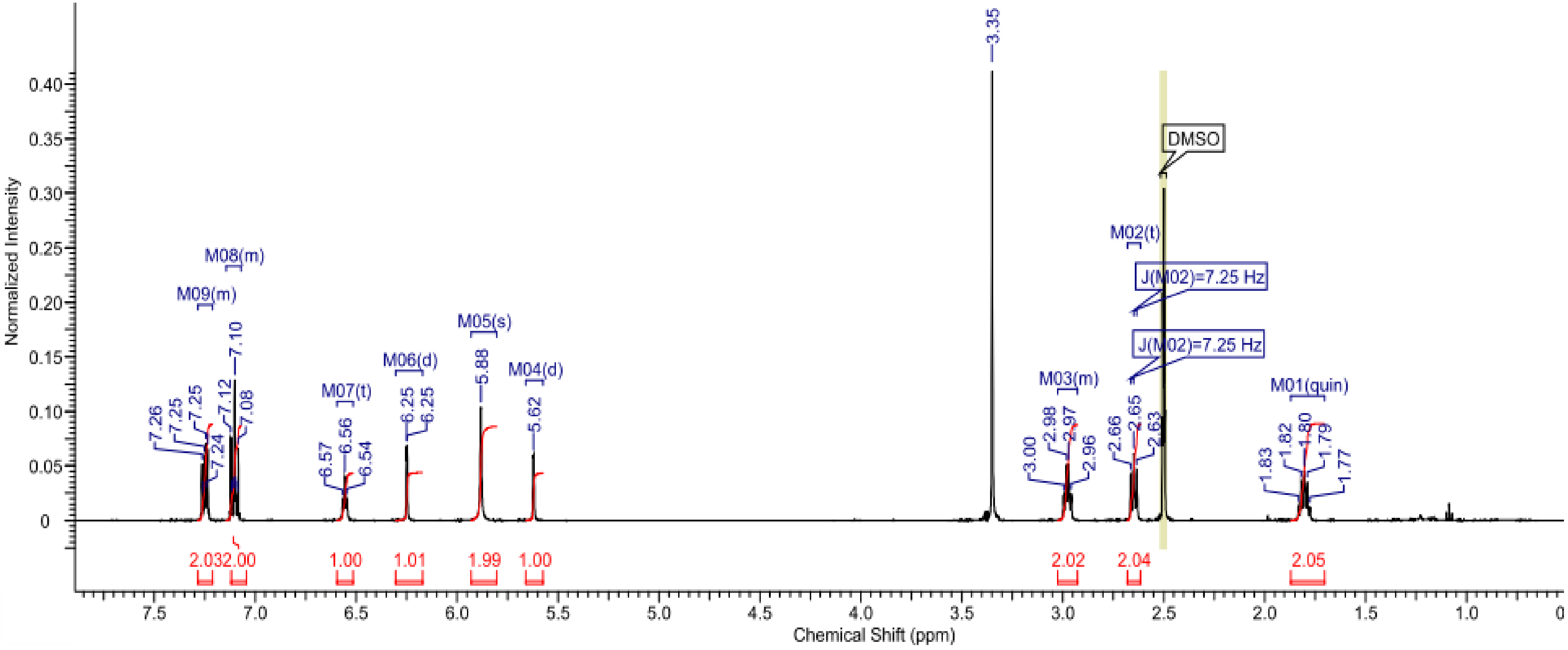

Compound 21

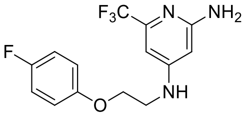

21

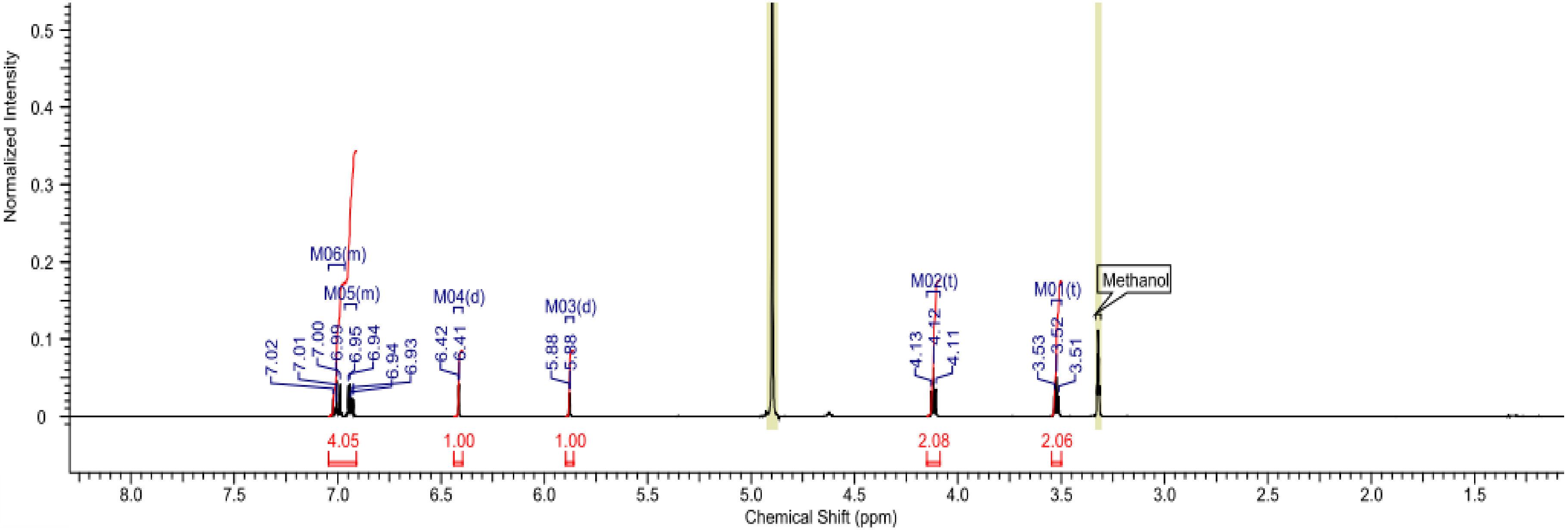

Compound 22

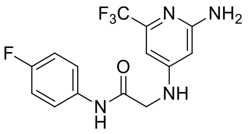


**21**

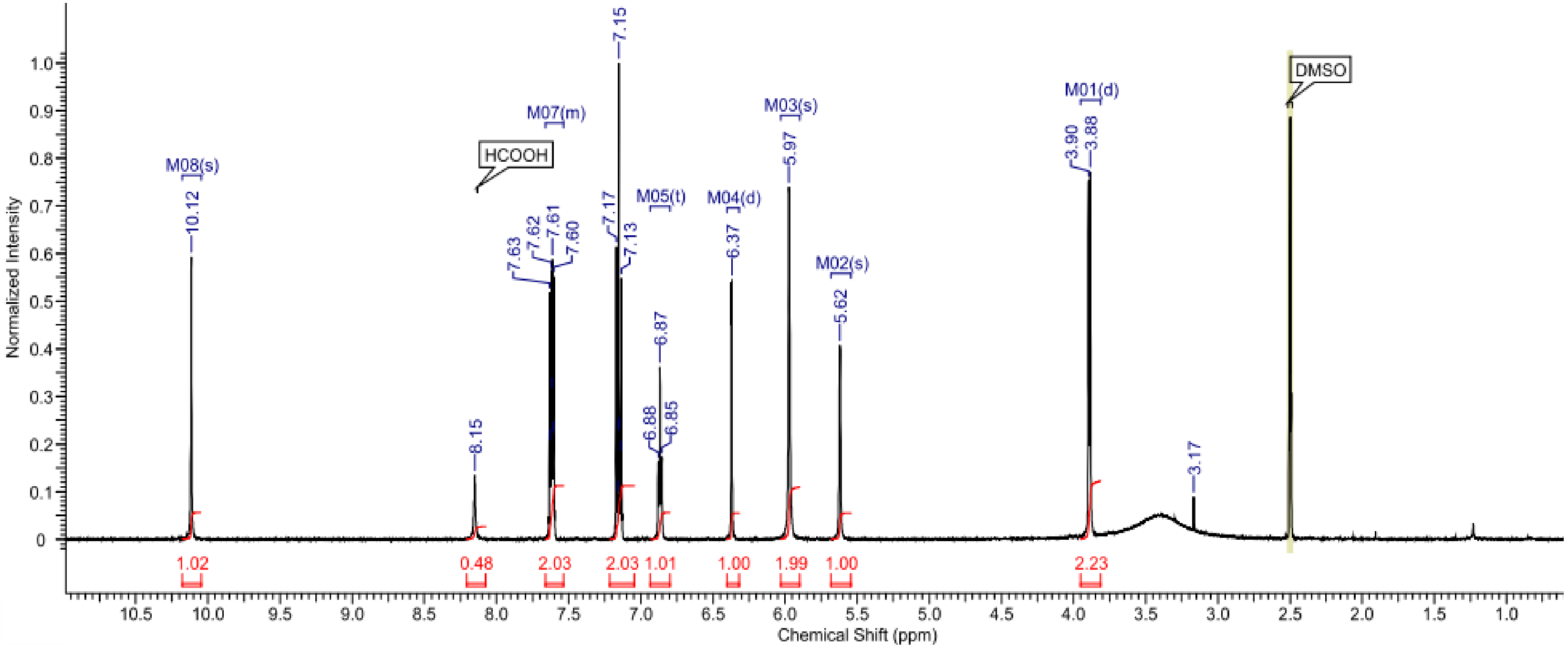

## 12. References


[1] F. M. Adam, G. Bish, F. Calo, C. L. Carr, N. Castro, D. Hay, P. B. Hodgson, P. Jones, C. J. Knight, M. Paradowski, G. C. Parsons, K. J. W. Proctor, D. C. Pryde, F. Rota, M. C. Smith, N. Smith, T.-D. Tran, J. Hitchin, R. Dixon, *Org. Process Res. Dev.* **2011**, *15*, 788–796.

[2] K. M. Maloney, E. Nwakpuda, J. T. Kuethe, J. Yin, *J Org Chem* **2009**, *74*, 5111–5114.

[3] Y. Liang, Y.-X. Xie, J.-H. Li, *J. Org. Chem.* **2006**, *71*, 379–381.

[4] N. Marion, O. Navarro, J. Mei, E. D. Stevens, N. M. Scott, S. P. Nolan, *J Am Chem Soc* **2006**, *128*, 4101–4111.

[5] Z. Lu, R. J. Twieg, *Tetrahedron Letters* **2005**, *46*, 2997–3001.

[6] J. E. Macor, B. L. Chenard, R. J. Post, *J. Org. Chem.* **1994**, *59*, 7496–7498.

[7] N. Marion, O. Navarro, J. Mei, E. D. Stevens, N. M. Scott, S. P. Nolan, *J. Am. Chem. Soc* **2006**, *128*, 4101–4111.

[8] T. Klein, C. Henn, J. C. de Jong, C. Zimmer, B. Kirsch, C. K. Maurer, D. Pistorius, R. Müller, A. Steinbach, R. W. Hartmann, *ACS Chem. Biol.* **2012**, *7*, 1496–1501.

[9] M. Zender, T. Klein, C. Henn, B. Kirsch, C. K. Maurer, D. Kail, C. Ritter, O. Dolezal, A. Steinbach, R. W. Hartmann, *J Med Chem* **2013**, *56*, 6761–6774.

[10] N. Xu, S. Yu, S. Moniot, M. Weyand, W. Blankenfeldt, *Acta Crystallogr Sect F Struct Biol Cryst Commun* **2012**, *68*, 1034–1039.

[11] W. Kabsch, *Acta Crystallogr D Biol Crystallogr* **2010**, *66*, 125–132.

[12] P. R. Evans, G. N. Murshudov, *Acta Crystallogr D Biol Crystallogr* **2013**, *69*, 1204–1214.

[13] M. D. Winn, C. C. Ballard, K. D. Cowtan, E. J. Dodson, P. Emsley, P. R. Evans, R. M. Keegan, E. B. Krissinel, A. G. W. Leslie, A. McCoy, S. J. McNicholas, G. N. Murshudov, N. S. Pannu, E. A. Potterton, H. R. Powell, R. J. Read, A. Vagin, K. S. Wilson, *Acta Crystallogr D Biol Crystallogr* **2011**, *67*, 235–242.

[14] I. J. Tickle, C. Flensburg, P. Keller, W. Paciorek, A. Sharff, C. Vonrhein, G. Bricogne, *STARANISO (http://staraniso.globalphasing.org/cgi-bin/staraniso.cgi)*, Cambridge, United Kingdom: Global Phasing Ltd., **2018**.

[15] A. J. McCoy, R. W. Grosse-Kunstleve, P. D. Adams, M. D. Winn, L. C. Storoni, R. J. Read, *J Appl Crystallogr* **2007**, *40*, 658–674.

[16] A. A. Vagin, R. A. Steiner, A. A. Lebedev, L. Potterton, S. McNicholas, F. Long, G. N. Murshudov, *Acta Crystallogr D Biol Crystallogr* **2004**, *60*, 2184–2195.

[17] A. Ilangovan, M. Fletcher, G. Rampioni, C. Pustelny, K. Rumbaugh, S. Heeb, M. Cámara, A. Truman, S. R. Chhabra, J. Emsley, P. Williams, *PLoS Pathog* **2013**, *9*, e1003508.

[18] P. Emsley, B. Lohkamp, W. G. Scott, K. Cowtan, *Acta Crystallogr D Biol Crystallogr* **2010**, *66*, 486–501.

[19] P. V. Afonine, R. W. Grosse-Kunstleve, N. Echols, J. J. Headd, N. W. Moriarty, M. Mustyakimov, T. C. Terwilliger, A. Urzhumtsev, P. H. Zwart, P. D. Adams, *Acta Crystallogr D Biol Crystallogr* **2012**, *68*, 352–367.

[20] P. D. Adams, P. V. Afonine, G. Bunkóczi, V. B. Chen, I. W. Davis, N. Echols, J. J. Headd, L.-W. Hung, G. J. Kapral, R. W. Grosse-Kunstleve, A. J. McCoy, N. W. Moriarty, R. Oeffner, R. J. Read, D. C. Richardson, J. S. Richardson, T. C. Terwilliger, P. H. Zwart, *Acta Crystallogr D Biol Crystallogr* **2010**, *66*, 213–221.

[21] M. S. Weiss, R. Hilgenfeld, *J Appl Crystallogr* **1997**, *30*, 203–205.

[22] K. Diederichs, P. A. Karplus, *Nat Struct Biol* **1997**, *4*, 269 EP -.

[23] M. S. Weiss, *J Appl Crystallogr* **2001**, *34*, 130–135.

[24] P. A. Karplus, K. Diederichs, *Science* **2012**, *336*, 1030–1033.

[25] V. B. Chen, W. B. Arendall, J. J. Headd, D. A. Keedy, R. M. Immormino, G. J. Kapral, L. W. Murray, J. S. Richardson, D. C. Richardson, *Acta Crystallogr D Biol Crystallogr* **2010**, *66*, 12–21.

[26] C. Lu, B. Kirsch, C. Zimmer, J. C. de Jong, C. Henn, C. K. Maurer, M. Müsken, S. Häussler, A. Steinbach, R. W. Hartmann, *Chem Biol* **2012**, *19*, 381–390.

[27] C. Cugini, M. W. Calfee, J. M. Farrow, D. K. Morales, E. C. Pesci, D. A. Hogan, *Mol Microbiol* **2007**, *65*, 896–906.

[28] D. W. Essar, L. Eberly, A. Hadero, I. P. Crawford, *J Bacteriol* **1990**, *172*, 884–900.

[29] C. Lu, C. K. Maurer, B. Kirsch, A. Steinbach, R. W. Hartmann, *Angew Chem Int Ed Engl* **2014**, *53*, 1109–1112.

[30] a) F. Lépine, S. Milot, E. Déziel, J. He, L. G. Rahme, *J Am Soc Mass Spectrom* **2004**, *15*, 862–869; b) F. Lépine, E. Déziel, S. Milot, L. G. Rahme, *Biochim Biophys Acta* **2003**, *1622*, 36–41.

[31] M. P. Storz, C. K. Maurer, C. Zimmer, N. Wagner, C. Brengel, J. C. de Jong, S. Lucas, M. Müsken, S. Häussler, A. Steinbach, R. W. Hartmann, *J Am Chem Soc* **2012**, *134*, 16143–16146.
